# A dynamic picture of the halolactonization reaction through a combination of *ab initio* metadynamics and experimental investigations†

**DOI:** 10.1039/d1sc01014j

**Published:** 2021-04-26

**Authors:** Ruben Van Lommel, Jonathan Bock, Constantin G. Daniliuc, Ulrich Hennecke, Frank De Proft

**Affiliations:** Eenheid Algemene Chemie (ALGC), Department of Chemistry, Vrije Universiteit Brussel (VUB) Pleinlaan 2 1050 Brussels Belgium fdeprof@vub.be; Molecular Design and Synthesis, Department of Chemistry, KU Leuven Celestijnenlaan 200F Leuven Chem&Tech, box 2404 3001 Leuven Belgium; Organic Chemistry Research Group (ORGC), Department of Chemistry, Department of Bioengineering Sciences, Vrije Universiteit Brussel (VUB) Pleinlaan 2 1050 Brussels Belgium Ulrich.Hennecke@vub.be; Institute for Organic Chemistry, University of Muenster Corrensstr. 40 48149 Münster Germany

## Abstract

The halolactonization reaction is one of the most common electrophilic addition reactions to alkenes. The mechanism is generally viewed as a two-step pathway, which involves the formation of an ionic intermediate, in most cases a haliranium ion. Recently, an alternative concerted mechanism was proposed, in which the nucleophile of the reaction played a key role in the rate determining step by forming a pre-polarized complex with the alkene. This pathway was coined the nucleophile-assisted alkene activation (NAAA) mechanism. Metadynamics simulations on a series of model halolactonization reactions were used to obtain the full dynamic trajectory from reactant to product and investigate the explicit role of the halogen source and solvent molecules in the mechanism. The results in this work ratify the occasional preference of a concerted mechanism over the classic two-step transformation under specific reaction conditions. Nevertheless, as the stability of both the generated substrate cation and counter-anion increase, a transition towards the classic two-step mechanism was observed. NCI analyses on the transition states revealed that the activating role of the nucleophile is independent of the formation and stability of the intermediate. Additionally, the dynamic insights obtained from the metadynamics simulations and NCI analyses employed in this work, unveiled the presence of *syn*-directing noncovalent interactions, such as hydrogen bonding, between the alkenoic acid and the halogen source, which rationalized the experimentally observed diastereoselectivities. Explicit noncovalent interactions between the reactants and a protic solvent or basic additive are able to disrupt these *syn*-directing noncovalent interactions, affecting the diastereoselective outcome of the reaction.

## Abstract 2

*Ab initio* dynamics of the halolactonization reaction provide insights into the diastereoselectivity of the reaction. Noncovalent interactions between the substrate, halogen source and solvent are revealed to direct the formation of the *syn*-product.

## Graphical abstract

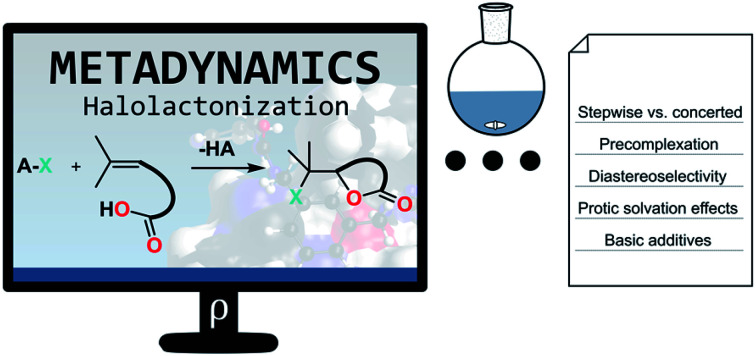

## Introduction

**Scheme 1:**
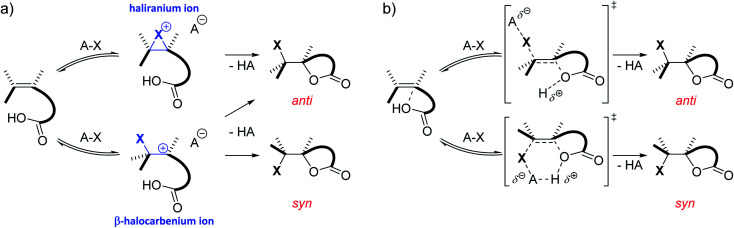
(a) Classic two-step mechanism for the electrophilic halogen addition to double bonds including the formation of an ionic intermediate phase. (b) Alternative concerted nucleophile-assisted alkene addition (NAAA) mechanism proposed by Jackson and Borhan. No cationic intermediate is formed and the nucleophile activates the double bond by forming a pre-polarized complex.

Besides substitution and elimination reactions, the addition of functional groups to unsaturated C–C bonds is one of the key chemical transformations.^1,2^ A well-known example of such a transformation is the electrophilic halogenation of alkenes. Classically, the mechanism of this reaction is supposed to proceed in two steps. In the first and rate determining step, a cationic intermediate is formed as the C=C double bond captures the electrophilic halogen atom. In the second step, a nucleophile attacks this positively charged intermediate, completing the addition reaction.^3,4^ This nucleophile can stem from either the halogen source, the solvent, an additive or the substrate itself. Depending on the type of substrate and reaction conditions, the cationic intermediate can be classified as the quintessential bridged haliranium or a β-halocarbenium ion. On the other hand, solvation determines whether these ions can be considered as tight ion pairs or solvent-separated ion pairs.^5^ Both the constitutional preference of the ion as well as its solvation determines the facial approach of the nucleophile with respect to the halogen, *i.e. syn-versus anti*-addition, and therefore the associated diastereomer of the product (Scheme 1a). If the alkene is prochiral and the halogen electrophile and nucleophilic species that add onto the double bond differ from one another, a total of up to eight isomeric products could be formed. Although the electrophilic halogen addition to alkenes has been studied for over 80 years, its regio- and stereochemical complexity and the control thereof make the transformation a challenging subject for synthetic chemists to this day.^6–11^

The halolactonization reaction is a class of electrophilic halogen additions which has attracted significant interest over the years.^12–16^ Herein, a carboxylic acid moiety of an alkenoic acid substrate acts as the nucleophile, resulting in an intramolecular cyclization forming a lactone. Recent research on the halolactonization reaction is particularly focussed on gaining stereoselective control by exploiting asymmetric catalysts.^14,17–24^ This field flourished greatly in the last decade with several types of highly selective chiral catalysts being developed by different research groups.^25–34^ Following these reports, enantioselective bromolactonization reactions of alkynes were developed by some of the authors of this work.^35,36^

Although numerous chiral catalysts have been developed for the enantioselective halolactonization reaction, their mode of action often remains elusive. Only very recently, the mechanistic aspects of the (DHQD)_2_PHAL catalysed asymmetric chlorolactonization reaction were unveiled by a joint effort by Jackson and Borhan.^37^ Kinetic studies were performed to establish that the reaction shows a first order dependence on both the catalyst and the halogen source. Furthermore, ROESY NMR spectroscopy and static DFT calculations provided a resting state complex and plausible transition states for the catalytic cycle. The foundation of this research is formed by other reports from both groups, investigating the reaction pathways of the non-catalysed halolactonization as well as other halocyclization reactions by means of a combination of experiments and static DFT calculations.^38^ In 2016, they proposed a concerted nucleophile-assisted alkene activation (NAAA) mechanism for a non-catalysed halolactonization reaction, supported by kinetic isotope effects and the nucleophile proximity observed during NMR experiments and static DFT calculations (Scheme 1b).^39^ Already more than 50 years ago, Shilov, among others, observed that the nucleophile could influence the rate and regioselectivity of electrophilic halogen additions, suggesting the classic mechanism was oversimplified in some cases.^40–42^ With the NAAA mechanism, a plausible alternative is introduced which can explain these early observations. Compared to the classic mechanism, the NAAA mechanism occurs concerted and no ionic intermediate is formed. Additionally, the nucleophile plays a vital role in the rate determining step of the reaction as it counter-intuitively forms a pre-polarized complex with the electron rich unsaturated bond. This complexation presumably activates the double bond by raising its HOMO energy level, enabling the electrophilic attack.

The elucidation of a reaction mechanism does not only improve the fundamental understanding of chemical transformations, it also aids in future catalyst design.^43,44^ State-of-the-art research, often uses an integrated computational and experimental approach to gain such knowledge, as exemplified by the collaborative works of Jackson and Borhan.^37–39^ On the computational side, static DFT calculations are routinely used, due to the availability of several user friendly software, limited hardware requirements and the extensive development of this approach.^45–47^ Most static DFT calculations simplify solvation effects by implicit solvation models, negating the possibility of observing explicit interactions between the solvent and reactants. In the past decade, researchers are paying more attention to previously overlooked solvent effects, by including explicit microsolvated clusters and developing new methods that maintain computational tractability at the DFT level of theory when adding explicit solvation molecules to the computed system.^48–50^ Notwithstanding that static DFT calculations provide valuable information concerning chemical reactions, they only provide knowledge limited to the stationary points of the potential energy surface. In this regard, *ab initio* molecular dynamics simulations has proven to be a valuable tool to study the full dynamical aspects of a chemical reaction, explicitly including all molecular entities that are present during the transformation.^51–54^ Recently, we have shown the usefulness of first-principles metadynamics (MtD) simulations to study the mechanisms of the electrophilic aromatic sulfonation and chlorination, with a focus on the formation of ionic intermediates during the reaction.^55,56^ In this work, a similar approach will be used to revisit the mechanism of the non-catalysed halolactonization. Emphasis will be put on the formation of a possible ionic intermediate, which differentiates the NAAA mechanism from the classic two-step mechanism. Furthermore, bond length and noncovalent interaction (NCI) analyses will reveal the specific role of the nucleophile and possible noncovalent interactions that might affect the diastereoselectivity of the reaction. Finally, the influence of the type of halogen source (chlorolactonization and bromolactonization), basic additives and the solvent will be investigated. The results of the MtD simulations will be compared to experimental observations made on the halolactonization reactions of the model substrates. In essence, we aim for this study to provide a better dynamic understanding of the prominent halolactonization reaction.

## Results and discussion

### Experimental results

**Scheme 2:**
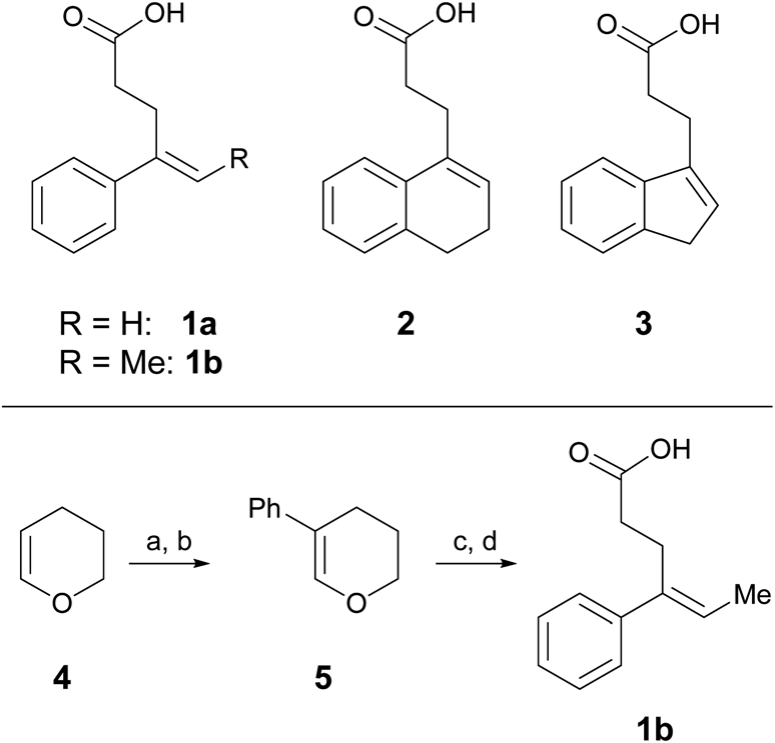
Alkenoic acids used as model starting materials. Synthesis of **1b**: (a) Br_2_, pyridine, 45%; (b) Pd_2_dba_3_, SPhos, PhB(OH)_2_, K_2_CO_3_, 70%; (c) NiCl_2_, IPr·HCl, MeMgBr, 86%; (d) TEMPO, PIDA, 60%.

One parameter in halolactonization reactions that should be significantly influenced by the mechanism is the diastereoselectivity of the addition. On the one hand, if a haliranium ion is formed, its opening by backside attack leads to exclusive formation of the *anti*-diastereomer. Formation of the *syn*-product, on the other hand, can be explained either by reaction *via* an open-chain β-halocarbenium ion or, according to the results of Jackson and Borhan, by a concerted reaction pathway involving NAAA. Therefore, we have chosen to investigate the diastereoselective outcome of the reaction, by carrying out an extensive set of selected halolactonization reactions (detailed results of the full experimental analysis can be found in the ESI†). The substrates of choice are derived from aryl-substituted pent-4-enoic acid, which undergo clean 5-exo cyclization (Scheme 2). 4-Phenyl-pent-4-enoic acid **1a** is a common substrate used in mechanistic studies and novel asymmetric halolactonization methods. To observe the diastereoselectivity of the reaction, a methyl group was introduced in the 5-position. This 4-phenyl-hex-4-enoic acid **1b** can be synthesized with exclusive (*E*)-geometry of the alkene following a novel, high yielding synthetic route (Scheme 1). Furthermore, cyclic alkenoic acids **2** and **3** were chosen as previous investigations have shown that these substrates provide unusually high amounts of *syn*-products in electrophilic halogenation reactions.^9^

Initial investigations quickly revealed that diastereoselectivity in the halolactonization of **1b**, **2** and **3** was indeed sensitive to reaction conditions and the halolactonization of **3** showed in general the highest amount of *syn*-products. Therefore, **3** was chosen as the initial model substrate.

**Fig. 1:**
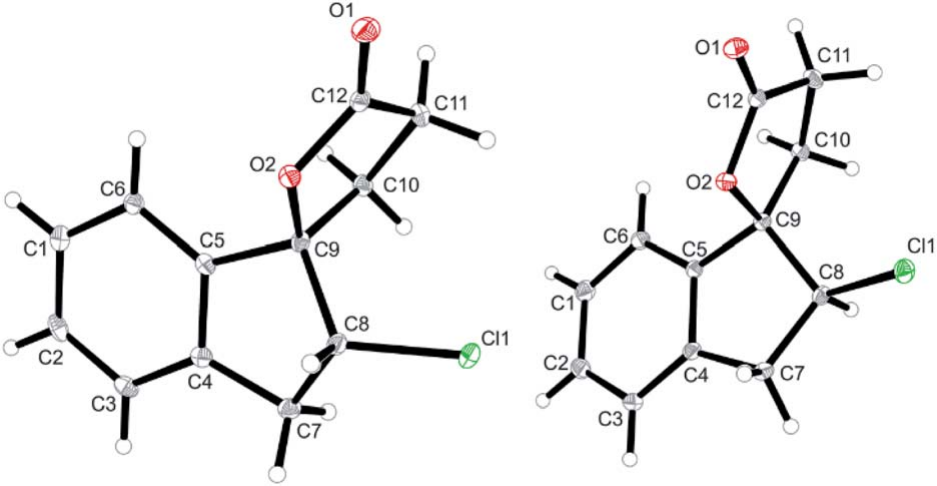
Solid state structures of *anti*-**6a** (left) and *syn*-**6a** (right) as obtained from X-ray crystallography (see ESI† for details, thermal ellipsoids are shown with 30% probability).

The halolactonization of **3** was studied at 0 °C in CH_2_Cl_2_ using different, electrophilic halogenating agents. 1,3-Dichloro-5,5-dimethylhydantoin (DCDMH) induced efficient 5-exo cyclization to provide chlorolactones *anti*-**6a** and *syn*-**6a** in good yield (69%) and with low diastereoselectivity (d.r. 60 : 40; Table 1, entry 2). The identity of the diastereomers was confirmed by X-ray structure analysis of the isolated products (Fig. 1).† Subsequent experiments showed that the diastereoselectivity was strongly dependent on the electrophilic halogenating agent and the halogen used. *N*-Bromosuccinimide (NBS) led to the bromolactones *anti*-**6b** and *syn*-**6b** in moderate yield and a slightly higher diastereoselectivity (71 : 29 d.r.; entry 3). Changing the relative amount of NBS with respect to the substrate **3** had a slight effect on the diastereoselectivity, with an increased formation of *anti*-**6b** when the relative amount of NBS increases (Table 1, entry 4 and 5). The more reactive 1,3-dibromo-5,5-dimethylhydantoin (DBDMH) provided a higher yield, but basically the same diastereoselectivity (d.r. 69 : 31, entry 6). A significant change in selectivity was observed when a reagent not based on a *N*-haloamide core was used. 2,4,4,6-Tetrabromo-2,5-cyclohexadienone (TBCO) induced bromolactonization in excellent yield (98%) and with very high diastereoselectivity (d.r. 97 : 3, entry 7). A very high diastereoselectivity was also observed when an electrophilic iodinating agent such as *N*-iodosuccinimide (NIS) was used, leading to the formation of almost pure *anti*-**6c** (95%, d.r. 97 : 3, entry 8). For comparison, also an electrophilic fluorinating agent, *N*-fluorobenzenesulfonimide (NFSI) was used. Fluorine cannot form fluoriranium ions, excluding a reaction path involving such an ion.^57–60^ NFSI proved to be much less reactive and the reaction could only be observed at RT in MeCN.^61,62^ Nevertheless, fluorolactonization proceed under these conditions in acceptable yield (62%) and with decent diastereoselectivity (d.r. 79 : 21, entry 1).

**Table 1:** Diastereomeric ratios (d.r.) and yields obtained for the halolactonization reactions of substrate **3**^a^

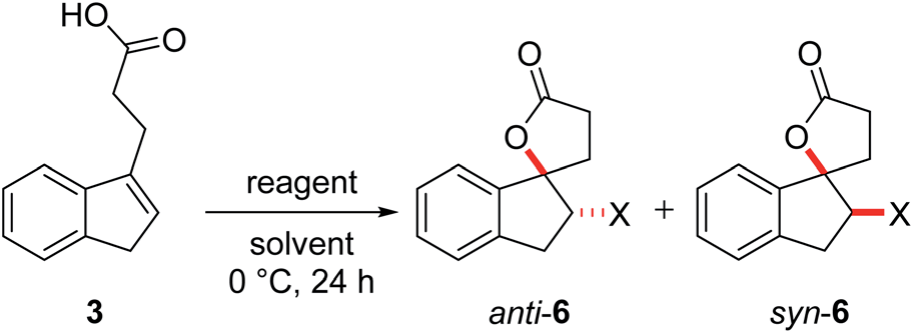				
Entry	Halogen source	Solvent	d.r.^b^ (*anti* : *syn*)	Yield^c^ (%)
**1** d	NFSI	MeCN	79 : 21	62
**2**	DCDMH	CH_2_Cl_2_	60 : 40	**6a**, 69
**3**	NBS	CH_2_Cl_2_	71 : 29	**6b**, 49
**4**	NBS (0.5)	CH_2_Cl_2_	66 : 34	**6b**, 40
**5**	NBS (2.0)	CH_2_Cl_2_	75 : 25	**6b**, 75
**6**	DBDMH	CH_2_Cl_2_	69 : 31	**6b**, 99
**7**	TBCO	CH_2_Cl_2_	97 : 3	**6b**, 98
**8**	NIS	CH_2_Cl_2_	97 : 3	**6c**, 96
**9**	NBS	MeOH	99 : 1	**6b**, 99
**10**	DCDMH	MeOH	99 : 1	**6a**, 99
**11** e	NBS	CH_2_Cl_2_	99 : 1	**6b**, 99
**12** e	DCDMH	CH_2_Cl_2_	99 : 1	**6a**, 99

a Reaction conditions: **3** (1.0 equiv.), halogen source (1.2 equiv.), in CH_2_Cl_2_ at 0 °C for 24 h.

b Determined by ^1^H-NMR of the crude reaction mixture.

c Determined by ^1^H-NMR of the crude reaction mixture using mesitylene as internal standard.

d Reaction at RT.

e Reaction with 1.0 equiv. quinuclidine added.

While halolactonization of **3** using electrophilic chlorinating as well as brominating agents in CH_2_Cl_2_ provided mixtures of product diastereomers only slightly favouring the *anti*-addition products, a remarkably different behaviour was observed in MeOH. For DCDMH as well as NBS, almost fully selective formation of the *anti*-addition products *anti*-**6a** or *anti*-**6b** in excellent yields was observed (entries 9 and 10). The *syn*-addition products were only formed in traces. A similar switch of selectivity could also be induced in CH_2_Cl_2_, if one equivalent of quinuclidine was added to the reaction (it is used as a model for cinchona alkaloid catalysts in asymmetric halolactonizations). This sped up the reactions and led to the almost exclusive formation of *anti*-addition products when using DCDMH or NBS as reagent, respectively.

**Scheme 3:**
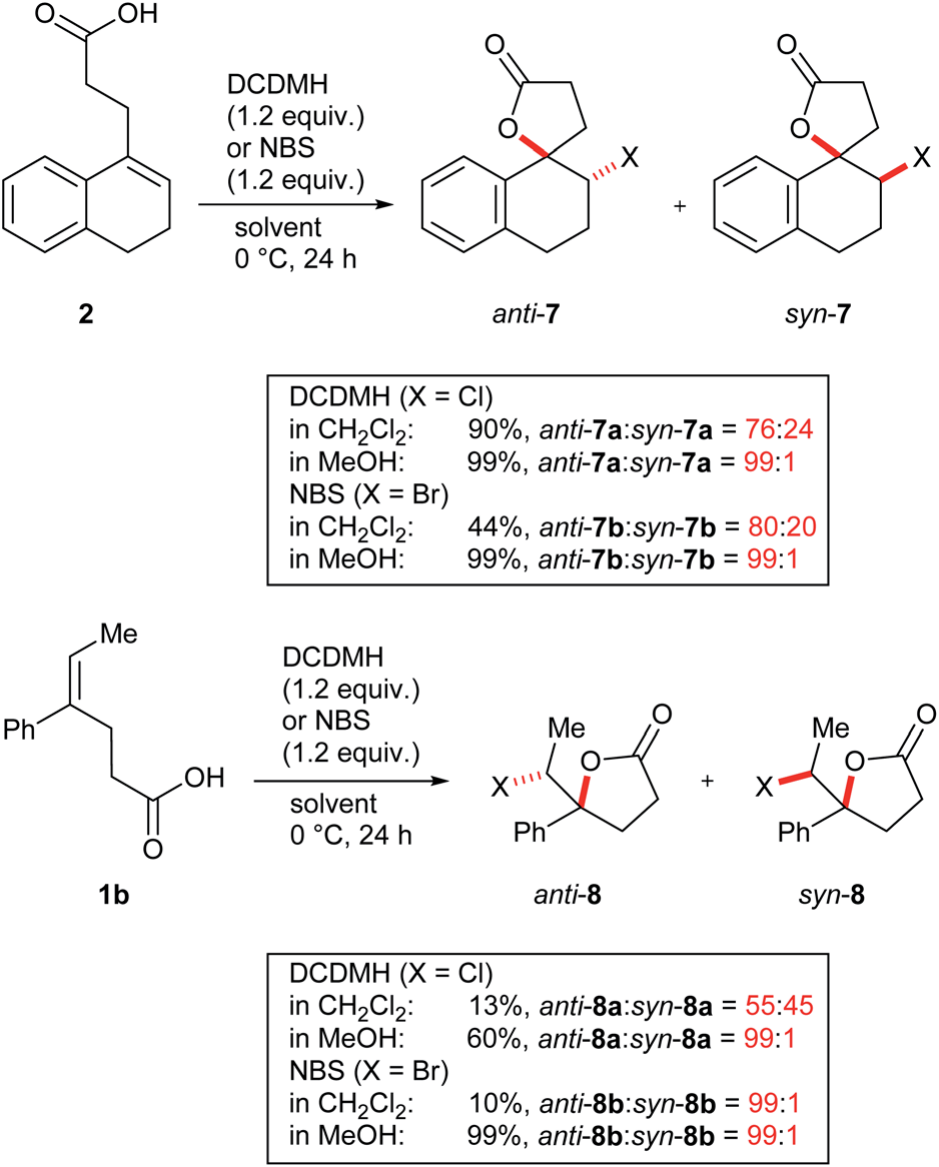
Chlorolactonization and bromolactonization of **1b** and **2**.

While the switch from a moderately diastereoselective halolactonization in CH_2_Cl_2_ to a fully selective reaction in MeOH was most pronounced for **3**, similar observations were also made for model compounds **1b** and **2** (Scheme 3 and ESI†). Cyclic alkenoic acid **2** underwent chlorolactonization using DCDMH in CH_2_Cl_2_ in good yield and with moderate diastereoselectivity (d.r. 76 : 24), while the reaction in MeOH was again highly selective towards *anti*-addition (d.r. 99 : 1). Compared to the cyclic alkenes **2** and **3**, open-chain alkenoic acid **1b** was less reactive and chlorolactonization using DCDMH in CH_2_Cl_2_ was slow and not diastereoselective (d.r. 55 : 45). In MeOH reactivity was much higher and the product *anti*-**8a** was obtained in 60% yield and excellent diastereoselectivity.

Overall, two clear trends could be observed: while reactions in CH_2_Cl_2_ showed only low to moderate diastereoselectivities, reactions in MeOH were in all cases highly selective for the *anti*-addition. A similar effect was observed when quinuclidine was added as a base to the reaction. Furthermore, diastereoselectivities increased when going from smaller chlorine to the larger iodine electrophiles in line with the observation that longer C–X bonds lead to more stable cyclic (“bridging”) haliranium ions.^63–65^

### Computational results: the halogen source matters

**Fig. 2:**
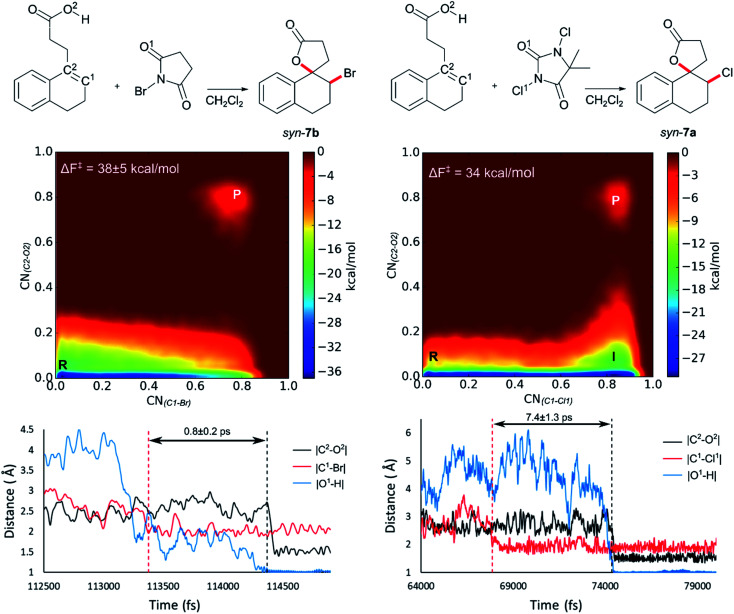
Analysis of the MtD simulations for the *syn*-mechanism of the NBS bromolactonization (left) and the DCDMH chlorolactonization of **2** (right) in CH_2_Cl_2_. At the top a schematic representation of both reactions is provided. The free energy surface of the corresponding reaction is given as an average over three and two independent MtD simulations respectively, together with the average Helmholtz free energy of activation Δ*F*^‡^, reactant phase (R), intermediate phase (I) and product phase (P). Bond length analysis of a single simulation is provided below. Here the dashed red line represents where the electrophilic addition takes place during the simulation, while the dashed black line delineates where the nucleophilic attack takes place causing ring closure. The time between these two events is defined as the lifetime of the intermediate phase. Average lifetime over the different simulations of the intermediate is provided between the two dashed lines.

Initially **2** was chosen as a model substrate for the calculations as the experimental results indicated an intermediate behaviour with respect to the diastereoselectivity. To obtain a plausible mechanistic pathway for this reaction, *ab initio* metadynamics simulations were performed with a simulation box including **2**, a halogenating agent, either NBS or DCDMH, and a number of CH_2_Cl_2_ molecules to match the experimental conditions and account for explicit solvation. The reaction coordinate was described by collective variables expressed as coordination numbers through which the reaction was driven. A more in-depth explanation of these collective variables can be found in the computational methods section. All simulations in this study were performed at the DFT level using the BLYP-D3 functional and the DZVP-MOLOPT-GTH plane wave basis set with the grid level cut-off set at 320 Ry (for detailed settings of the MtD simulations see ESI†).^66,67^ Surprisingly, the MtD simulations of **2** in CH_2_Cl_2_ with NBS as brominating source, always resulted in the formation of *syn*-**7b**, independent of the starting topology of the simulation. The full trajectory of the reaction shows that the *syn*-mechanism has an asynchronous concerted character (MV_S1). Before reaction takes place, an intermolecular hydrogen bond is present between the carboxylic acid moiety of **2** and an amide carbonyl group of NBS, which directs the *syn*-addition. Following this pre-reactive complexation, the electrophilic bromine is captured by the double bond. This event is rapidly followed by the simultaneous deprotonation of the carboxylic acid and intramolecular lactone formation. From the free energy surface (FES), only the reactant and product phase are observable minima, suggesting no intermediate is formed during the reaction (Fig. 2). Furthermore, through bond length analyses it is possible to determine the time between the initial electrophilic addition and nucleophilic ring closure, essentially quantifying the lifetime of the ionic intermediate phase. For the *syn*-selective reaction of **2** with NBS in CH_2_Cl_2_, a relative short average lifetime of 0.8 ± 0.2 ps is observed. Both the FES and bond length analyses indicate the concerted asynchronous character of the mechanism. Moreover, prior to bromine addition, the nucleophile (O^2^) and the double bond (C^2^–C^1^) are in proximity to each other as a distance of approximately 2.5 Å between them is observed. The latter could be interpreted as the nucleophile activating the double bond by forming a pre-polarized complex. Both the concerted asynchronous character of the reaction and the proximity of the nucleophile to the double bond, point towards the NAAA mechanism.

By investigating the corresponding DCDMH chlorolactonization of **2** in CH_2_Cl_2_, we aimed to elucidate the effect of the halogenating source on the reaction mechanism. Our experimental results indicated that the preference towards the *anti*-product increases together with the size of the halogen for the halolactonization reaction of **3**, following the trend Cl^+^ (DCDMH) < Br^+^ (NBS) < I^+^ (NIS) (Table 1). Notable, a similar trend was previously observed by Jackson and Borhan for the halolactonization of **1a**.^39^ Their initial reasoning for this trend, was that for larger halogen atoms, the mechanism giving rise to the *syn*-product would be characterized by an increased steric repulsion, favouring the *anti*-product. To investigate whether the enhanced preference for the *anti*-product could be attributed to the size of the halogen atom, noncovalent interaction (NCI) analyses on the transition states for the *syn*-halolactonization reaction of **2** with NBS and DCDMH were performed. Latter technique allows to identify the noncovalent interactions in a molecular system by means of the relationship between the reduced density gradient (*s*) and the product of the electron density (*ρ*) and the sign of the second eigenvalue of the electron density Hessian matrix (sign *λ*_2_).^68^ Peaks appearing in the corresponding 2D-plot at low absolute values of *ρ*sign(*λ*_2_) are caused by noncovalent interactions. If the peak occurs at positive values of *ρ*sign(*λ*_2_), the interaction is repulsive. If the peak occurs at negative values the interaction is attractive. Additionally, the interactions can be visualized by plotting an isosurface of *s*, with an RGB colour scale indicating if the interaction is repulsive (red), weakly attractive (green) or strongly attractive (blue).

**Fig. 3:**
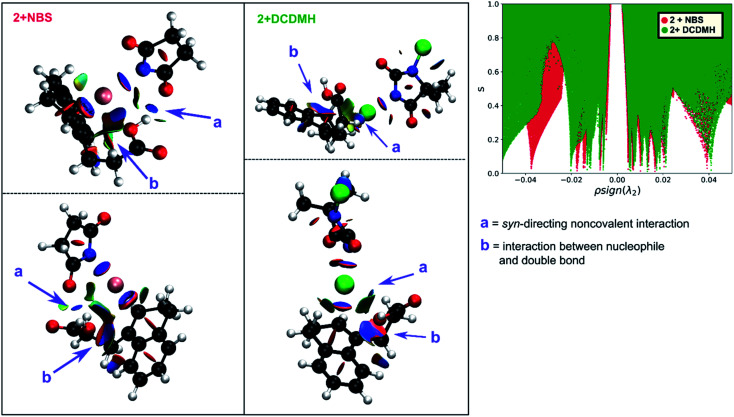
Two different perspectives of the 3D-NCI isosurfaces of the reduced density gradient *s* indicating the noncovalent interactions that are present in the transition state of the NBS *syn*-bromolactonization (left, red) and DCDMH *syn*-chlorolactonization (middle, green) of **2**. The surfaces (*s* = 0.5) are coloured by an RGB-scale according to *ρ*sign(*λ*_2_) ranging from −0.01*5* a.u. to 0.01*5* a.u. An intermolecular *syn*-directing hydrogen bond is present for the NBS bromolactonization, indicated by a purple arrow accompanied with the letter a. While a similar hydrogen bond is absent for the corresponding DCDMH chlorolactonization, an attractive interaction (blue) between Cl^+^ and the α-hydrogen is present in this case (purple arrow a). (b) A noncovalent interaction between the nucleophile and the double bond suggests the activating role of the nucleophile. At the right side an overlay plot of *s* against *ρ*sign(*λ*_2_) is provided for the transition state of the NBS (red) and DCDMH (green) *syn*-halolactonization of **2**.

The analysis reveals that both transition states are characterized by similar repulsive interactions as peaks in the positive region of the 2D-plot of *s* against *ρ*sign(*λ*_2_) overlap entirely (Fig. 3). This excludes steric repulsion induced by a larger halogen atom to be the reason behind the change in diastereoselectivity. However, when looking at the negative region of the 2D-plot, *i.e.* attractive interactions, significant differences are observable. Indeed, upon closer inspection of the 3D-NCI isosurfaces, it is clear that for the NBS *syn*-bromolactonization of **2**, a *syn*-directing hydrogen bond between both reactants is present (highlighted in Fig. 3 by the purple arrow “a”). In contrast, a similar hydrogen bond is absent for the DCDMH *syn*-chlorolactonization of **2**. In the latter case, however, an attractive noncovalent interaction between the α-hydrogen of the carboxylic acid and the incoming halogen is present, which is able to fulfil a similar role as the hydrogen bond in steering the *syn*-addition of the nucleophile (again this interaction is highlighted in Fig. 3 by the purple arrow “a”). These findings are a first indication that a fundamental change in the reaction mechanism occurs when altering the halogen source from NBS to DCDMH. Also, when comparing the free energy surfaces and bond length analyses of the above-mentioned reactions, clear differences are observable (Fig. 2). For the DCDMH chlorolactonization of **2**, the presence of an intermediate phase (I) characterized by a high value of CN_(C1–Cl1)_ and a low value of CN_(C2–O2)_ is observable. From these values it is discerned that this structure corresponds to a classic ionic intermediate. Indeed, also from bond length analyses one can imply the existence of a more stable intermediate, as the lifetime of this phase (7.4 ± 1.3 ps) has increased considerably with respect to the NBS *syn*-bromolactonization of **2** (0.8 ± 0.2 ps).‡‡During a metadynamics simulation, a chemical system is forced to move from stable states by means of added Gaussian potentials. As such, these lifetimes are artificially decreased and therefore cannot be directly compared to actual lifetimes. Nevertheless, trends concerning stability of stable states can still be retrieved when the shape and frequency of the added Gaussian potentials remains the same over the different simulations. We suspect this trend to be explained by the larger inherent stability of the dechlorinated counter-anion of DCDMH (MCDMH^−^) compared to the corresponding debrominated succinimide anion of NBS (succ^−^) (this statement is supported by static heterolytic BDE calculations, see ESI†). Due to the clear formation of an ionic intermediate during the reaction, it is concluded that the DCDMH *syn*-chlorolactonization of **2** follows a two-step reaction pathway. It must be mentioned, however, that bond length analyses indicated the nucleophile (O^2^) to be in proximity (∼3.0 Å) of the double bond before the halogen addition occurs. Also, from the 3D-NCI isosurface, a noncovalent attractive interacting area can be detected between the nucleophile (O^2^) and the double bond in the transition state (this interaction is highlighted in Fig. 3 by the purple arrow “b”). As such, the concerted nature of the NAAA reaction mechanism is not a prerequisite for the pre-activation of the double bond by the nucleophilic species.

The mechanistic disparities between the NBS and DCDMH *syn*-halolactonization of **2** showcase that the type of halogen source affects the mode of action. To determine whether the reaction pathway occurs through a two-step mechanism or the concerted NAAA mechanism, the stability of all ionic species that are generated during the course of the reaction need to be considered. This includes the cationic β-halocarbenium ion and the corresponding counter-anion generated from the halogen source. When the stability of both ionic species increases, a transition from the concerted NAAA mechanism towards a two-step mechanism occurs. On the other hand, *syn*-directing noncovalent interactions between the alkenoic acid and the halogen source rationalize the substantial formation of *syn*-product for the bromo- and chlorolactonization of **2**. In the former case, a *syn*-directing hydrogen bond is present between the carboxylic acid and amide group of NBS. One can deduct that if a halogenating source were to be used where such interaction would be missing, an increased diastereoselectivity towards the *anti*-product could be observed. And indeed, when TBCO (a brominating agent which has no *ortho* carbonyl group to facilitate hydrogen bond formation) was used during the halolactonization of **3** (Table 1, entry 7), the amount of *anti*-diastereomer that is formed increases significantly compared to other brominating sources with an *ortho*-oriented carbonyl group (NBS, DBDMH).

In addition to **2**, the bromo- and chlorolactonization reaction of **1a** was explored by MtD simulations in order to study possible effects of the substrate on the mechanism. Again, for the NBS bromolactonization, the *syn*-mechanism was readily observed, guided by an intermolecular hydrogen bond between NBS and the substrate. Further analysis of the trajectory, bond lengths and free energy surface reveals other similar characteristics compared to the NBS *syn*-bromolactonization reaction of **2** (see ESI†). Indeed, no distinct intermediate phase is observed from the free energy surface and the corresponding average lifetime thereof is relatively small, being 0.9 ± 0.3 ps. Moreover, the carboxylic acid oxygen (O^2^), which acts as the nucleophile in the reaction, is near the alkene (∼3.0 Å) before electrophilic addition of the halogen to the double bond takes place. This indicates the activation of the double bond through complexation. With these features in mind, the concerted NAAA mechanism is suggested as the reaction pathway for the NBS *syn*-bromolactonization reaction of **1a** in CH_2_Cl_2_. When the corresponding DCDMH chlorolactonization of **1a** is studied, a similar pathway is obtained (see ESI†). An inter-molecular hydrogen bond between the halogen source and the substrate directs the *syn*-addition, the nucleophile is in proximity of the double bond and thus is able to activate the unsaturated bond by forming a pre-polarized complex. And finally, no distinct ionic intermediates are observed in the free energy surface and its limited lifetime (1.0 ± 0.5 ps). These observations indicate the DCDMH *syn*-chlorolactonization of **1a** to follow the concerted NAAA mechanism. Notably, the same reaction with **2** occurs through a two-step mechanism. This highlights that both the stability of the cation generated from the substrate and the counter-anion from the halogen source need to be considered when investigating the mechanism of the halolactonization reaction.

### Basic additives enable full *anti*-diastereoselectivity

In previous sections only the *syn*-halolactonization mechanism was discussed as the *anti*-mechanism could not be retrieved from the MtD simulations. Not even by setting various geometrical constraints on the reactive system during the MtD simulation. Nevertheless, the experimental results showed a clear preference towards formation of the *anti*-diastereoisomer for all reactions, independent of the halogen source and substrate. Moreover, this effect is increased when quinuclidine is added to the reaction mixture as a base. Besides the diastereoselectivity towards the *anti*-product, the halolactonization shows a significant increase in yield when quinuclidine is added (Table 1, entry 12).

**Fig. 4:**
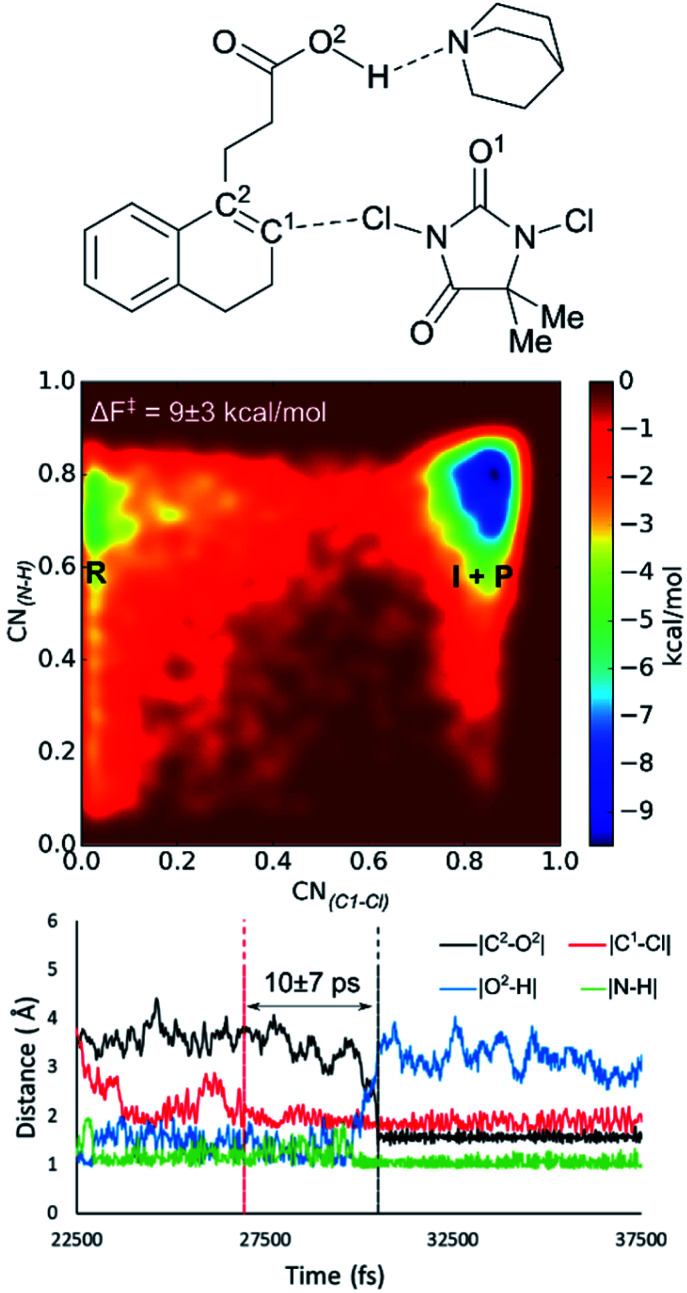
When quinuclidine is added in the MtD simulations, the *anti*-mechanism is retrieved exclusively for the DCDMH chlorolactonization of **2**. The free energy surface averaged over three MtD simulations is provided. Note that the intermediate phase (I) and the product phase (P) overlap due to the choice of the collective variables. From bond length analyses, the lifetime of the halonium intermediate is determined and the hydrogen bonded halonium–quinuclidine complex is observed.

When including a quinuclidine molecule in the system for the DCDMH halolactonization of **2** in CH_2_Cl_2_, the *anti*-mechanism was exclusively observed from the MtD simulations, coinciding with the experimental observed increased *anti*-diastereoselectivity (Fig. 4). Furthermore, the activation barrier is reduced significantly when quinuclidine is included in the simulation box (Δ*F*^‡^ = 9 kcal mol^−1^). The latter is consistent with the experimentally observed increased yield. Before the reaction takes place, deprotonation of the carboxylic acid by the quinuclidine base occurs. The resulting protonated quinuclidine remains connected to the carboxylate by means of hydrogen bonding in a pre-reactive complex, preventing the formation of any *syn*-directing interaction with the halogen source. This explains the diastereoselectivity of the reaction towards the *anti*-product. In the next step, halogen addition occurs without pre-polarization of the double bond by the nucleophile as the distance between the nucleophile and the double bond prior to halogen addition is relatively large (∼4 Å). Furthermore, a long-lived intermediate phase is formed (10 ± 7 ps), likely due to the inherent stability of MCDMH^−^ and the cationic substrate-quinuclidine complex. In the final step, lactonization occurs which is accompanied by the simultaneous breaking of the substrate-quinuclidine hydrogen bond. These results suggest that when quinuclidine is added as a base to the DCDMH halolactonization reaction of **2**, a two-step mechanism is followed, which results exclusively in the *anti*-product. The experimentally observed diastereoselectivity is explained by the absence of a *syn*-directing noncovalent interaction between the nucleophile and the halogen source. Summarized, molecules which are able to interact with the carboxylic acid group of the alkenoic acid through hydrogen bonding, facilitate formation of the *anti*-product by competing with *syn*-directing noncovalent interactions.

With these new insights, we again set out to retrieve a plausible mechanism for the non-catalysed *anti*-halolactonization. Inspired by Jackson and Borhan, who could only localize such a transition state by explicitly adding a second halogen source molecule to their static calculations, we were also able to suggest a plausible transition state for the *anti*-halolactonization of **2** with NBS and DCDMH using static DFT calculations (see ESI†). As such we thought it might be necessary to include two pairs of reactants in the simulation box to observe the dynamics of the *anti*-mechanism. Unfortunately, this approach did not yield the *anti*-addition, not when a second halogen source molecule was added to the system nor a second substrate molecule. Notably, in the latter case dimerization of the two substrate molecules occurred instantaneous through hydrogen bonding of their carboxylic acid groups. When a succinimide anion was included in the simulation as a base for the NBS bromolactonization of **2**, a mechanism resulting in the *anti*-product was observable, where the succinimide base fulfils a similar role as the quinuclidine in the *anti*-halolactonization mechanism described above (see ESI†). Seemingly basic molecules that can disrupt the *syn*-directing interactions and deprotonate the substrate are necessary to retrieve the *anti*-addition from the MtD simulations. However, it remains ambiguous to us which molecule fulfils this role in the non-catalysed *anti*-halolactonization. It might be possible that there are molecules missing from the system which are crucial for the *anti*-mechanism and thus prevent us from observing it.

### Protic solvation effects

Reflecting on the afore mentioned findings, we conclude that noncovalent interactions between the reactants play an important role during the halolactonization reaction. For example, hydrogen bonding between the nucleophile and the halogen source directs the *syn*-addition. If the reaction is performed in an aprotic medium such as CH_2_Cl_2_, the solvent will most likely not significantly influence these noncovalent interactions. Indeed, in all MtD simulations where CH_2_Cl_2_ was included as a solvent, no explicit interactions between the solvent and reactants were observed. Results also indicate that the diastereoselectivity towards the *anti*-product increases when these *syn*-directing noncovalent interactions are disrupted. Naturally, the question arises whether a solvent can be used to accomplish this. As the experimental diastereoselectivities of the model reactions in MeOH all showed almost exclusive *anti*-product formation as well as an increase in yield compared to when the reaction is performed in CH_2_Cl_2_, one would assume that this were to be the case. Therefore, in this paragraph, MtD simulations will be used to investigate how a protic solvent (MeOH) affects the diastereoselective outcome and mode of action of the halolactonization reaction.

**Fig. 5:**
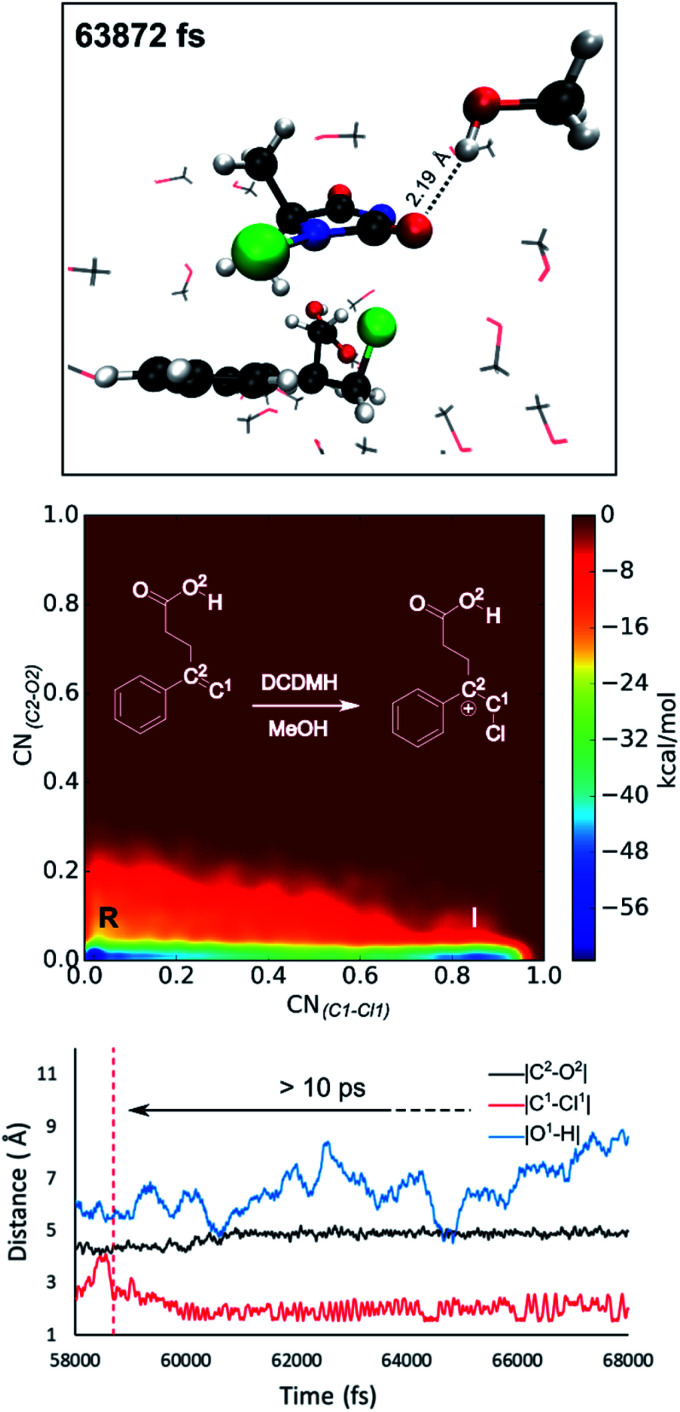
DCDMH halonium formation of **1a** in MeOH. The intermediate phase is stabilized due to the formation of a hydrogen bond between a MeOH molecule and the anionic dechlorinated MCDMH^−^. This hydrogen bond is visualized for a snapshot of the MtD simulation. No *syn*-directing interactions are observed between the reactants due to explicit competing interactions with the solvent.

From an MtD simulation of the DCDMH chlorolactonization of **1a** in MeOH, the clear formation of a halonium intermediate is observed. This conclusion is based on the free energy surface as well as the extended lifetime of the intermediate phase that can be determined from bond length analyses (>10 ps) (Fig. 5). Notably, the nucleophile is not in proximity of the double bond before halogen addition takes place (∼4.5 Å) and no *syn*-directing intermolecular interactions between the nucleophile and halogen source are observed during the transition towards the halonium intermediate. Therefore, it is concluded that the DCDMH chlorolactonization of **1a** in MeOH follows the classic mechanism without pre-activation of the double bond by the nucleophile or any *syn*-directing interactions. Note that when the simulations were carried out in CH_2_Cl_2_, the concerted NAAA mechanism was favoured. When scrutinizing the explicit role of the protic solvent on the reaction mechanism, hydrogen bonds between MeOH and the acid functionality of the substrate are observable. Seemingly the solvent competes with and disrupts any *syn*-directing interactions such as hydrogen bonding between the substrate and the halogen source. Similar to when the reaction is performed in presence of a quinuclidine base, the absence of *syn*-directing interactions can rationalize the experimental diastereoselectivity towards the *anti*-product. Moreover, after halogen addition has taken place, a stable hydrogen bond is observed between a MeOH molecule and the dechlorinated MCDMH^−^ molecule, which sustains the anionic species (Fig. 5). This stabilizing effect explains the considerable lifetime of the ionic intermediate phase and the preference towards the two-step mechanism.

## Conclusion

In this work, an elaborate experimental and computational investigation was performed on the halolactonization of alkenoic acids. By carrying out a set of halolactonization reactions and performing the corresponding *ab initio* metadynamics simulations, we aimed to elucidate the diastereoselectivity and mechanistic aspects of this chemical transformation. Focus was put on two viable reaction pathways, being the textbook two-step mechanism and a recently proposed concerted nucleophile-assisted alkene addition mechanism. Both mechanisms differ from one another by the respective presence or absence of an ionic intermediate phase. Our theoretical results reveal that a concerted mechanism is preferred in some cases. However as the stability of both the generated β-halocarbenium intermediate and halogen source counter-anion increase, a transition towards the classic two-step mechanism is observed. Notably, *via* NCI analysis it was revealed that the nucleophile can activate the double bond by forming a pre-polarized complex during the concerted as well as the classic two-step mechanism, meaning that the activating role of the nucleophile is not determined by the probability that an intermediate phase is formed. Reactions carried out in this study, resulted in the formation of the *syn*- and *anti*-diastereomer product in varying ratios. By scrutinizing the results obtained from the *ab initio* simulations, *syn*-directing noncovalent interactions such as hydrogen bonding between the nucleophile and the halogen source are revealed, which control the formation of the *syn*-product. As this mechanism is in competition with a pathway that generates the *anti*-product, disruption of these *syn*-directing interactions by a basic additive or a protic solvent such as MeOH, results in a strongly increased diastereoselectivity towards the *anti*-product. With that, this study showcases, that through careful selection of reagents and/or reaction conditions, the diastereoselectivity of the halolactonization reaction can be regulated. Moreover, when the *syn*-directing interactions are negated, and additionally the ionic intermediate phase has a significant lifetime, the activating role of the nucleophile gets diminished. This study affirms the importance of taking dynamic solvent effects explicitly into account when studying a mechanism where ionic intermediates are conceivably formed. We aim for these new insights to assist the field in developing new strategies to control the stereoselective outcome of the halolactonization reaction.

## Computational methods

### Metadynamics

To obtain the dynamic trajectories of the chemical reactions studied in this work, metadynamics (MtD) simulations were performed with the CP2K code (version 6.1) using the Quickstep implementation.^69,70^ This method improves the sampling efficiency of conventional *ab initio* molecular dynamics (AIMD) by artificially adding Gaussian potentials along a set of carefully selected collective variables (CVs) that adequately describe the reaction coordinate. In this manner, the potential energy surface is flattened and uniform sampling of the rare event, *i.e.* chemical reaction, is achieved.^71,72^

All AIMD and MtD simulations were performed at the DFT level using the BLYP functional and the DZVP-MOLOPT-GTH plane wave basis set with the grid level cut-off set at 320 Ry.^66,67^ For bromine atoms the short range version of the basis set (DZVP-MOLOPT-SR-GTH) was used. Additionally, Grimme's D3 dispersion corrections were used to take into account long range dispersion interactions.^73^ The dispersion-corrected GGA exchange correlation functional BLYP-D3 has previously shown to adequately describe the dynamical aspects of both concerted and multistep elementary organic reactions.^55,74^ Simulations were performed in the NVT ensemble where the temperature was set by the CSVR thermostat at 273 K and a timeconstant of 1 fs, matching the experimental reaction conditions. The reactants and solvent molecules were placed in a periodic cubic box with a box edge size of 15 Å and sufficient solvent molecules to match the experimental density of the solvent for CH_2_Cl_2_ (1.33 g cm^−3^) and MeOH (0.792 g cm^−3^) using the Packmol software.^75^ Specifications concerning the simulation box can be found in the ESI.†

Each system underwent an equilibration phase before initializing the MtD simulation by running a short AIMD simulation (5000 steps, with a timestep of 1 fs rendering a total simulation time of 5 ps), allowing the system to relax. Following this, Gaussian potentials with a height of 2.0 kJ mol^−1^ and a width of 0.02 were added every 25 steps along the specified collective variables for the first 20 000 steps of the simulation. After this, the hill height was reduced to 1.0 kJ mol^−1^ to avoid oversampling. If during the simulation the chemical reaction takes place in the first 20 000 steps, the simulation was repeated with the hill height immediately set to 1.0 kJ mol^−1^. In all cases the collective variables were defined as coordination numbers (CNs) for which the expression is provided in eqn (1). In this expression *r*_*ij*_ defines the distance between atom *i* and *j* and *r*_0_ is a predefined value.1
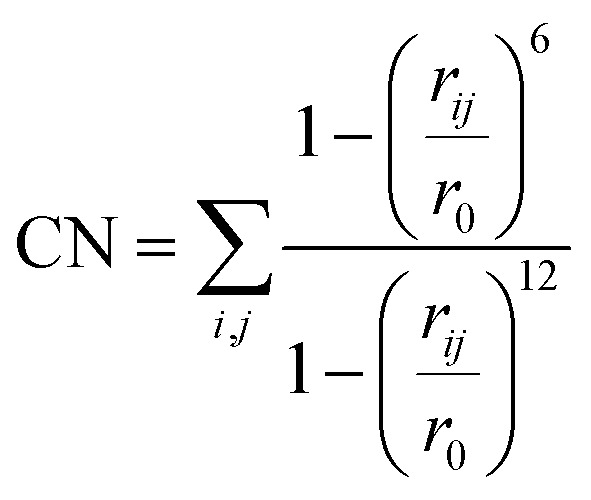


To limit the sampling region, half harmonic biased potentials were added for some simulations and the mass of all protons was set to the mass of tritium to reduce hydrogen vibration frequencies that cause the simulation to be unstable at the 1 fs time step. Detailed information concerning the collective variables and bias potentials of each reactive system and an example input file is provided in the ESI.† In general, MtD simulations sample many forward and reverse rare events to ensure both reactant and product wells are filled and convergence of the simulation is reached. For chemical reactions, however, the reverse step is often unobtainable. For this reason systems for which the activation barrier was determined underwent multiple MtD simulations of the single forward reaction step and results were averaged over these simulations to ensure statistical relevancy. Each simulation ran an excess of 5 ps after the chemical reaction has taken place from which free energy surfaces were constructed.

### Analysis of simulations

From the canonical ensemble partition function, the Helmholtz free energy of activation, Δ*F*^‡^ was computed by eqn (2). Here, *k*_b_ denotes the Boltzmann constant, *T* the temperature, *P*_TS_ the population at the transition state and *P*_sum_ the total probability of the reactant state.2
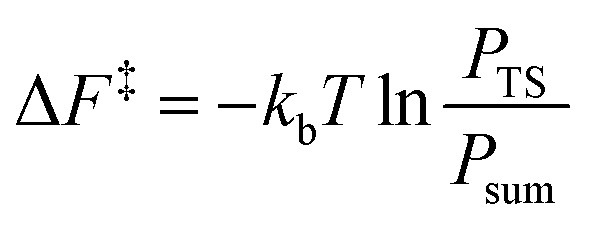


The most accurate transition state was determined as the structure right before a large jump of the collective variable which describes the addition of the electrophilic halogen to the double bond is observed. To determine the lifetime of the charged ionic intermediate, the simulation time between the formation of the carbon–halogen bond and nucleophilic attack was established by tracking the appropriate bond lengths of the reactive system.

### Noncovalent interaction indices

When plotting the reduced density gradient (*s*) against the electron density *ρ*, noncovalent interaction peaks appear in the plot at low density values (eqn (3)). Furthermore, attractive and repulsive interactions can be discerned from one another by the second eigenvalue of the electron density Hessian matrix (*λ*_2_). These aspects form the basis of a noncovalent interaction indices (NCI) analysis.^68^3
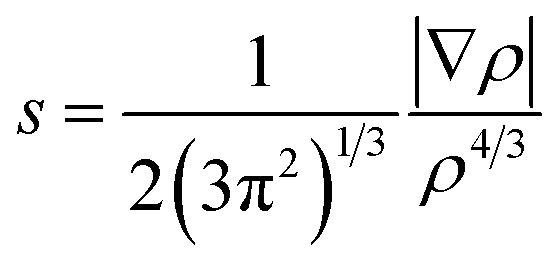


NCI analyses were performed on the transition state structures obtained from the MtD simulations as previously explained. These analyses allowed to elucidate the role of various noncovalent interactions on the mechanistic preference of the reaction. Electron densities were obtained by performing single point energy calculations on the MtD transition state structure at the DFT level of theory using the B3LYP-D3 functional and the double-ζ 6-31G(d) basis set with the Gaussian quantum chemistry package version 16 revision B.01.^76^ One of the advantages of using the NCI analysis is the exceptional robustness of the technique with respect to the method and basis set used to generate the electron density.^66,77,78^ During the analysis, explicit solvent molecules which are present during the MtD simulation, were left out to focus on the noncovalent interactions between the reactants and simplify the interpretation of the results. The NCIPLOT program version 3.2.3 was used to perform the analyses and visualization of isosurfaces of the reduced density gradient *s* was accomplished by the VMD software version 1.9.3.^79,80^

## Author contributions

R. V. L., J. B., U. H. and F. D. P. helped conceptualize and write this research. R. V. L. planned, carried out and analysed the theoretical simulations. J. B. planned, carried out and analysed the experiments in this work. C. G. D. provided the necessary resources and carried out X-ray experiments and analysis. U. H. and F. D. P. supervised the project.

## Conflicts of interest

There are no conflicts to declare.

## Supplementary Material

SC-012-D1SC01014J-s001

SC-012-D1SC01014J-s002

SC-012-D1SC01014J-s003
