# Supplementary material for: A dynamic picture of the halolactonization reaction through a combination of ab initio metadynamics and experimental investigations
Source: Chem Sci. 2021 Apr 26;12(22):7746–57. doi: 10.1039/d1sc01014j (PMC8188468; doi:10.1039/d1sc01014j)
Supplement: SC-012-D1SC01014J-s001 [file SC-012-D1SC01014J-s001.pdf]

Electronic Supplementary Material (ESI) for Chemical Science.
This journal is © The Royal Society of Chemistry 2021

Electronic Supplementary Information for:

A Dynamic Picture of the Halolactonization Reaction through a Combination of ab-initio Metadynamics and Experimental Investigations

Ruben Van Lommel[a,b], Jonathan Bock[c], Constantin G. Daniliuc[d], Ulrich Hennecke[c], and Frank De Proft[a]

a. General Chemistry - Eenheid Algemene Chemie (ALGC), Department of Chemistry, Vrije Universiteit Brussel (VUB), Pleinlaan 2, 1050 Brussels, Belgium

b. Molecular Design and Synthesis, Department of Chemistry, KU Leuven, Leuven Chem&Tech, Celestijnenlaan 200F, 3001 Leuven, Belgium

c. Organic Chemistry Research Group (ORGC), Departments of Chemistry and Bioengineering Sciences, Vrije Universiteit Brussel (VUB), Pleinlaan 2, 1050 Brussels, Belgium

d. Institute of Organic Chemistry, University of Münster, Corrensstr. 40, 48149 Münster, Germany

Correspondence: ulrich.hennecke@vub.be; fdeprof@vub.be

Electronic Supporting Information

- Table of contents -

1. Computational studies **3**

1.1. Ionic stabilities of the halogen sources 3

1.2. Metadynamics simulations 4

1.3. Comparing the NBS syn-halolactonization of 1a and 2 24

1.4. Static transition states for the anti-halolactonization of 2 26

2. Synthetic procedures and characterization **29**

2.1. General information 29

2.2. Synthesis of starting materials 30

2.3. Synthesis and characterization of halolactonization products 33

2.3.1. Halolactonization of 3-(1H-inden-3-yl)propanoic acid (3) 33

2.3.2. Halolactonization of 3-(3,4-dihydronaphthalen-1-yl)propanoic acid (2) 35

2.3.3. Halolactonization of (E)-4-phenylhex-4-enoic acid (1b) 37

2.4. Effects of substrates and reaction conditions on diastereoselectivity 38

2.5. Crystallographic data 40

2.6. ^{1}H*- and* $^{13}C\{^{1}H\}$*-NMR-spectra 47*

3. References **62**

1. Computational studies

1.1. Ionic stabilities of the halogen sources

Metadynamics simulations revealed that the ionic intermediate phase formed during the halolactonization reaction had an increased lifetime when DCDMH was used as a halogen source instead of NBS. To investigate the inherent ionic stability of these halogen sources, heterolytic bond dissociation energies were computed for the following reactions:

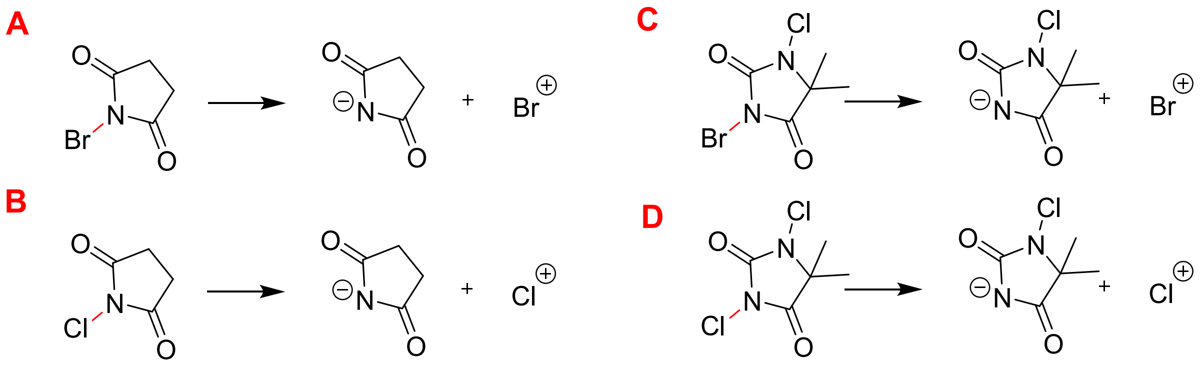

All structures were optimized at the DFT-level of theory, with vibrational analysis to confirm they represent actual minima on the potential energy surface, *i.e.* no imaginary frequencies were observed. Optimization and frequency calculations were performed with the 6-31+G(d) basis set and the B3LYP[1], M06-2X[2] or ωB97X-D[3] hybrid exchange-correlation functionals. Subsequently, to obtain more accurate electronic energies, single point calculations were performed on the structures with the 6-311++G(d,p) basis set and the B3LYP, M06-2X or ωB97X-D functional, respectively. For every calculation CH_2Cl_2 was taken into consideration by the SMD implicit solvent model.[4] All calculations were performed with the Gaussian software version 16 revision B.01.[5]

Table S1: Computed heterolytic bond dissociation energies (BDE) in kcal/mol at the DFT level of theory using different exchange-correlation functionals and the 6-31+G(d) basis set for geometry optimizations and frequency analysis and the 6-311++G(d,p) basis set to obtain more accurate electronic energies. CH_2Cl_2 was implicitly taken into consideration by the SMD solvent model.

reaction	BDE (kcal/mol) [B3LYP/6-311++G(d,p) //B3LYP/6-31+G(d)]	BDE (kcal/mol) [M06-2X/6-311++G(d,p) //M06-2X/6-31+G(d)]	BDE (kcal/mol) [ωB97X-D /6-311++G(d,p) // ωB97X-D /6-31+G(d)]
A	209	210	213
B	218	220	222
C	185	180	187
D	194	195	195

From Table S1 it can be observed that the computed BDE associated with hydantoin **C** and **D** are smaller than their respective succinimide derivative **A** and **B**. This indicates the anion $MCDMH^-$ to be inherently more stable relative to its neutral brominated or chlorinated species compared to the succinimide anion. As such, the increased ionic stability of $MCDMH^-$ compared to $succ^-$, might be a possible explanation for the halonium intermediate phase observed during MtD simulations of the NBS and DCDMH halolactonization reactions.

1.2. Metadynamics simulations

Table S2: Summary of results of the MtD simulations including the average free Helmholtz energy of activation ΔF‡ (in kcal/mol) and the average lifetime of the halonium intermediate phase (in ps). [a] *As the standard deviation over the different simulations is below the chemical accuracy of 1 kcal/mol, it is not physically relevant to mention.*

Entry	Substrate	Halogen source	additives	medium	ΔF‡ (kcal/mol)	Lifetime intermediate phase (ps)	Type of reaction
1	2	NBS	None	CH_2Cl_2	38 ± 5	0.8 ± 0.2	*syn*-addition
2	2	DCDMH	none	CH_2Cl_2	34[a]	7.4 ± 1.3	*syn*-addition
3	1a	NBS	None	CH_2Cl_2	30 ± 8	0.9 ± 0.3	*syn*-addition
4	1a	DCDMH	None	CH_2Cl_2	26 ± 5	1.0 ± 0.5	*syn*-addition
5	2	DCDMH	Quinuclidine	CH_2Cl_2	9 ± 3	10 ± 7	*anti*-addition
6	2	NBS	Succinimide$^-$	CH_2Cl_2	13 ± 1	0	*anti*-addition
7	1a	DCDMH	None	MeOH	N.A.	> 10	Halonium formation

Below technical details and results of the separate runs can be found of the metadynamics simulations that were performed in this study.

Entry 1: NBS *syn*-halolactonization in CH_2Cl_2

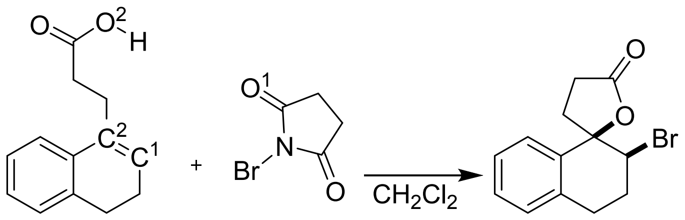

Table S3: Settings for the metadynamics simulation for the abovementioned reaction. [a] A half harmonic bias potential was added to CN1 at a value of 0.015 and force constant K = 300 a.u. to reduce the sampling region to a meaningful chemical space.

reactants	Box edge (Å)	CN1 / r0 [a]	CN2 / r0
1 x alkenoic acid<br>1 x NBS<br>29 x CH_2Cl_2	15.0	C^1-Br / 2.5 Å	C^2-O^2 / 1.9 Å

Input example

```
&FORCE_EVAL
 METHOD QS
 &DFT
  &POISSON
   PERIODIC XYZ
   POISSON_SOLVER PERIODIC
  &END POISSON
  BASIS_SET_FILE_NAME BASIS_MOLOPT
  POTENTIAL_FILE_NAME GTH_POTENTIALS
  CHARGE 0
  &QS
  &END QS
  &XC
   &XC_FUNCTIONAL BLYP
   &END XC_FUNCTIONAL
   &VDW_POTENTIAL
    POTENTIAL_TYPE PAIR_POTENTIAL
    &PAIR_POTENTIAL
     TYPE DFTD3
     PARAMETER_FILE_NAME dftd3.dat
     REFERENCE_FUNCTIONAL BLYP
    &END PAIR_POTENTIAL
   &END VDW_POTENTIAL
  &END XC
  &SCF
   EPS_SCF 1.0E-5
   SCF_GUESS RESTART
   &OT
    MINIMIZER DIIS
    PRECONDITIONER FULL_SINGLE_INVERSE
   &END OT
  &END SCF
  &MGRID
    CUTOFF 320
    COMMENSURATE TRUE
  &END MGRID
 &END DFT
 &SUBSYS
  &COORD
C      4.838114      6.849784      3.326318
C      6.429801      7.331582      1.657330
N      5.180381      6.741426      1.987742
O      3.835318      6.613213      3.955374
O      6.846998      7.461228      0.503401
C      6.059942      7.455349      4.040401
H      5.868559      8.291880      4.682805
H      6.352453      6.611009      4.702356
```

C	7.113361	7.778123	2.975936
H	7.334237	8.837458	2.891800
H	8.033955	7.269962	3.136714
C	5.828216	-1.055906	2.577314
C	4.652757	-0.731019	3.229762
C	3.972931	0.441721	2.827121
C	4.456534	1.147176	1.709940
C	5.534323	0.655776	0.942865
C	6.205537	-0.490784	1.351949
H	6.441404	-1.869984	2.879205
H	4.399042	-1.329091	4.108054
C	3.020877	1.113146	3.680611
H	5.940806	1.226284	0.130918
H	7.032715	-0.807871	0.748513
C	2.719093	2.447749	3.495027
H	2.016081	2.928382	4.156676
Br	4.127094	6.133360	0.403371
C	3.806597	2.394495	1.204535
H	4.492109	2.860049	0.522692
H	2.959367	2.018350	0.603868
C	3.265493	3.288421	2.338674
H	2.460840	3.908782	1.936694
H	4.023488	3.993574	2.673860
C	2.297355	0.333256	4.806619
H	1.397071	0.922701	5.085384
H	1.969424	-0.637512	4.488445
C	3.120030	0.124893	6.210581
H	4.112824	-0.160281	5.986249
H	3.076767	1.131636	6.731849
C	2.610614	-0.951006	7.177342
O	2.029346	-0.867331	8.244718
O	2.912210	-2.225754	6.717279
H	2.843270	-2.982255	7.326291
C	9.523774	11.005958	15.893499
H	9.738909	10.582371	16.862543
H	9.867119	10.315236	15.134385
Cl	10.453536	12.570619	15.784652
Cl	7.712593	11.105169	15.786025
C	7.799463	18.378210	2.066791
H	7.544874	17.320387	2.001460
H	8.845282	18.525229	2.343933
Cl	6.861324	18.996405	3.508912
Cl	7.384301	19.150904	0.531532
C	12.714472	12.619846	10.849446
H	13.256837	13.027907	11.689892
H	12.025579	11.835930	11.165651
Cl	13.877178	11.983190	9.700497
Cl	11.813651	13.993917	10.124018

C	12.847427	3.453314	9.359944
H	12.353053	3.119151	8.462396
H	12.913206	2.647925	10.081202
Cl	11.967731	4.871071	10.047639
Cl	14.516862	3.942819	8.879947
C	4.040282	3.850007	7.415512
H	3.277408	4.359575	6.798590
H	4.603074	3.123993	6.865340
Cl	3.101584	3.010742	8.811930
Cl	5.139250	5.060515	8.112773
C	10.034111	9.144401	10.796816
H	10.819495	9.469442	10.141384
H	9.043207	9.246570	10.363782
Cl	10.106265	10.392284	12.139953
Cl	10.311867	7.453098	11.303292
C	15.946344	1.400978	11.159465
H	16.443359	0.872999	10.337765
H	15.875811	2.466272	11.008709
Cl	16.922251	1.151582	12.661951
Cl	14.262259	0.779038	11.219245
C	3.170470	-2.719686	13.773540
H	2.247531	-2.626185	14.359041
H	3.732100	-1.796378	13.770863
Cl	2.729717	-3.098630	12.070352
Cl	4.180169	-4.039082	14.473455
C	7.096974	11.398760	10.507352
H	7.493927	10.378794	10.625906
H	6.763128	11.817360	11.456656
Cl	5.676782	11.248116	9.446013
Cl	8.425808	12.436571	9.831285
C	7.677307	8.209777	7.505361
H	8.127238	8.019833	6.517852
H	8.116596	9.076271	8.024903
Cl	7.949410	6.740536	8.504937
Cl	5.936723	8.590041	7.266852
C	12.226093	12.720559	5.691219
H	11.542796	12.908727	6.494808
H	12.354126	11.677622	5.477891
Cl	13.845757	13.361769	6.226501
Cl	11.516580	13.471969	4.209361
C	13.092255	1.887309	4.834118
H	12.104309	2.072100	5.204010
H	13.203129	0.831649	4.500023
Cl	14.291760	2.241973	6.094365
Cl	13.383192	3.029255	3.450089
C	6.552401	7.526224	12.451666
H	5.870075	7.618728	13.274786
H	7.453693	8.086922	12.689023

Cl	5.780865	8.170462	10.951550
Cl	6.984269	5.761546	12.506995
C	4.559723	-0.405122	-4.450524
H	3.926666	-1.253738	-4.686770
H	3.964401	0.453705	-4.118496
Cl	5.499048	0.003397	-5.925290
Cl	5.599965	-0.859462	-3.014329
C	16.856207	8.514284	6.635624
H	16.169153	7.709389	6.481896
H	17.560677	8.642423	5.815372
Cl	17.753935	8.124578	8.146252
Cl	15.768465	9.992736	6.557328
C	3.618553	10.322738	2.650515
H	3.579896	10.643346	1.621669
H	3.356340	9.279788	2.734588
Cl	2.493723	11.273046	3.696942
Cl	5.403882	10.417480	3.028117
C	8.278879	-2.769963	5.375618
H	8.989748	-2.698715	6.188056
H	8.371973	-2.012034	4.573454
Cl	6.627245	-2.615495	6.070301
Cl	8.411623	-4.400132	4.596615
C	16.102915	5.709886	3.273644
H	17.173479	5.693817	3.358204
H	15.636762	4.743330	3.097268
Cl	15.755126	6.727693	1.791317
Cl	15.407964	6.381366	4.737836
C	8.793729	3.202250	9.895439
H	8.275528	3.238116	8.932934
H	9.296820	4.165428	10.061618
Cl	10.087461	1.908960	9.721539
Cl	7.602823	2.912455	11.226585
C	2.576757	5.404263	11.238306
H	3.384690	5.406686	10.505499
H	1.710521	4.814136	10.905916
Cl	2.040304	7.137090	11.401657
Cl	3.267793	4.653842	12.732072
C	10.486900	6.481908	-0.538943
H	11.441171	6.820677	-0.936850
H	9.623336	7.042299	-0.920925
Cl	10.525974	6.645964	1.271943
Cl	10.261443	4.707278	-0.838500
C	10.641695	0.112152	13.646553
H	10.778876	0.222653	14.720682
H	11.177347	-0.726497	13.200446
Cl	11.391562	1.637576	12.966175
Cl	8.868342	-0.127641	13.367776
C	-1.267990	3.464659	13.924078

```
H       -1.399737       2.470324       13.505143
H       -2.199569       3.976706       14.159065
Cl      -0.450263       4.399123       12.619987
Cl      -0.261455       3.314945       15.416118
C       -2.635037       9.900282       7.419341
H       -2.823951       9.378396       6.458629
H       -1.961741       10.739405      7.318411
Cl      -1.978428       8.763581       8.637645
Cl      -4.296723       10.582952      7.875012
C       12.545206       9.467820       2.555253
H       12.533798       8.499783       2.072972
H       12.345024       10.342292      1.929448
Cl      14.136685       9.735041       3.318803
Cl      11.128187       9.442457       3.716669
C       8.275598        15.589074      7.048544
H       8.243999        15.825401      8.112866
H       7.840342        14.612764      6.817240
Cl      10.065825       15.397125      6.587838
Cl      7.382383        16.857458      6.137672
C       11.414692       5.334611       6.351935
H       12.038004       5.031889       5.513674
H       11.903802       5.610489       7.298985
Cl      10.235029       4.003622       6.676638
Cl      10.513349       6.808880       5.733418
C       -0.499953       12.938456      1.907416
H       -1.404224       13.317301      2.377425
H       0.008805        12.173651      2.501158
Cl      0.664939        14.267584      1.644802
Cl      -0.876698       12.301741      0.266027
C       -0.354292       8.837976       -1.448378
H       -1.069176       9.440248       -0.873038
H       -0.613713       7.783806       -1.351689
Cl      1.272463        9.086796       -0.628162
Cl      -0.542111       9.332324       -3.179132
  &END COORD
  &CELL
   ABC 15 15 15
   PERIODIC XYZ
  &END CELL
  #C-X
  &COLVAR
   &COORDINATION
    ATOMS_FROM 25
    ATOMS_TO 23
    R0 [angstrom] 2.5
   &END COORDINATION
  &END COLVAR
  #C-O
```

```
    &COLVAR
      &COORDINATION
        ATOMS_FROM 40
        ATOMS_TO 20
        R0 [angstrom] 1.9
      &END COORDINATION
    &END COLVAR
    &TOPOLOGY
    &END TOPOLOGY
    &KIND C
      BASIS_SET DZVP-MOLOPT-GTH
      POTENTIAL GTH-BLYP-q4
    &END KIND
    &KIND H
      MASS 3
      BASIS_SET DZVP-MOLOPT-GTH
      POTENTIAL GTH-BLYP-q1
    &END KIND
    &KIND Cl
      BASIS_SET DZVP-MOLOPT-GTH
      POTENTIAL GTH-BLYP-q7
    &END KIND
    &KIND O
      BASIS_SET DZVP-MOLOPT-GTH
      POTENTIAL GTH-BLYP-q6
    &END KIND
    &KIND Br
      BASIS_SET DZVP-MOLOPT-SR-GTH
      POTENTIAL GTH-BLYP-q7
    &END KIND
    &KIND N
      BASIS_SET DZVP-MOLOPT-GTH
      POTENTIAL GTH-BLYP-q5
    &END KIND
  &END SUBSYS
&END FORCE_EVAL
&GLOBAL
  PRINT_LEVEL LOW
  PROJECT md
  RUN_TYPE MD
&END GLOBAL
&MOTION
  &PRINT
    &RESTART_HISTORY
      &EACH
        MD 2000
      &END EACH
    &END RESTART_HISTORY
```

```
  &TRAJECTORY
   &EACH
    MD 2
   &END EACH
   FORMAT DCD
  &END TRAJECTORY
 &END PRINT
 &MD
  ENSEMBLE NVT
  STEPS 10000
  TIMESTEP 1.0
  TEMPERATURE 273
  &THERMOSTAT
   TYPE CSVR
   &CSVR
    TIMECON 1
   &END CSVR
  &END THERMOSTAT
 &END MD
 &FREE_ENERGY
  &METADYN
   DO_HILLS T
   NT_HILLS 25
   WW [kjmol] 2.0
#THE ABOVE MENTIONED VALUE IS CHANGED TO 1.0 AFTER THE FIRST 20000 STEPS
   &METAVAR
   COLVAR 1
   SCALE 0.02
    &WALL
     TYPE QUADRATIC
     POSITION 0.015
     &QUADRATIC
      DIRECTION WALL_MINUS
      K 300.0
     &END QUADRATIC
    &END WALL
   &END METAVAR
   &METAVAR 2
   COLVAR 2
   SCALE 0.02
   &END METAVAR
   &PRINT
    &COLVAR
     COMMON_ITERATION_LEVELS 2
     &EACH
      MD 1
     &END
     FILENAME=colvar
```

```
      &END COLVAR
      &HILLS
        COMMON_ITERATION_LEVELS 2
        &EACH
          METADYNAMICS 1
        &END
        FILENAME=hills
      &END HILLS
    &END PRINT
  &END METADYN
 &END FREE_ENERGY
&END MOTION
```

Topology of transition state excluding solvent molecules

C	11.604025	22.251074	16.620943
C	10.715115	20.136637	16.480591
N	10.482812	21.489279	16.325882
O	11.612569	23.489079	16.548344
O	9.828062	19.277048	16.253330
C	12.771374	21.242630	16.913122
H	13.587726	21.246223	16.180212
H	13.268368	21.637072	17.817030
C	12.138899	19.859222	16.875696
H	12.618002	19.159197	16.177937
H	11.999473	19.396502	17.927929
C	5.311684	18.585590	11.466502
C	5.331314	19.312786	12.610317
C	5.958196	20.588655	12.857016
C	6.674187	21.102703	11.706112
C	6.684909	20.338453	10.538164
C	6.076901	19.079502	10.405871
H	4.744869	17.668264	11.424134
H	4.719610	18.984484	13.428138
C	5.758946	21.291422	14.080765
H	7.396240	20.754776	9.804934
H	6.090851	18.496634	9.568016
C	6.347799	22.612534	14.241278
H	5.875778	23.236412	14.970949
Br	8.232181	22.155119	15.272311
C	7.340229	22.487490	11.869251
H	8.369924	22.248842	12.217394
H	7.510005	22.906181	10.859870
C	6.644282	23.349491	12.892442
H	5.787216	23.867884	12.572070
H	7.291136	24.181192	13.077649
C	4.774813	20.820019	15.102249
H	4.018838	21.570530	15.211550

H 4.239591 19.862921 14.952074

C 5.375153 20.550293 16.549868

H 5.861098 21.356447 17.006855

H 4.585859 20.242493 17.223026

C 6.471231 19.494291 16.537340

O 6.809676 18.790415 17.479797

O 7.005839 19.359051 15.335077

H 7.812448 18.820229 15.473013

Table S4: Helmholtz free energy of activation (in kcal/mol) and the lifetime of the halonium intermediate determined based on bond length analysis for the separate runs.

Run	ΔF‡ (kcal/mol)	Lifetime halonium intermediate (fs)	Total simulation time before TS (fs)
1	44	994	114406
2	35	568	99920
3	35	844	95487

Entry 2: DCDMH *syn*-halolactonization in CH_2Cl_2

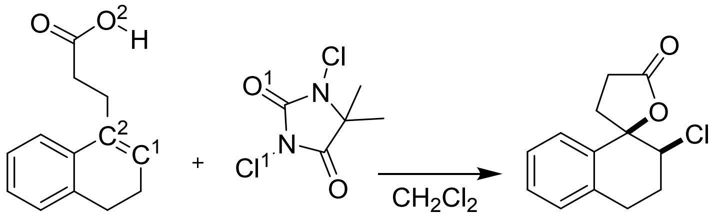

Table S5: Settings for the metadynamics simulation for the abovementioned reaction. [a] A half harmonic bias potential was added to CN1 at a value of 0.015 and force constant K = 300 a.u. to reduce the sampling region to a meaningful chemical space.

reactants	Box edge (Å)	CN1 / r0 [a]	CN2 / r0
1 x alkenoic acid<br>1 x DCDMH<br>28 x CH_2Cl_2	15.0	C^1-Cl^1 / 2.5 Å	C^2-O^2 / 1.9 Å

Topology of transition state excluding solvent molecules

N 15.63643 -2.70016 2.014617

C 16.420616 -3.472024 0.980034

C 17.144089 -2.223583 0.304982

C 15.500712 -1.378983 1.687194

N 16.394455 -1.12887 0.593928

Cl 17.701818 0.690777 0.658275

O 18.175911 -2.231641 -0.307999

O 14.898301 -0.498946 2.320794

C	17.564882	-4.405681	1.541097
H	17.132242	-5.341853	1.885089
H	18.05715	-3.876095	2.351033
H	18.270123	-4.544726	0.774944
C	15.486877	-4.18997	-0.005428
H	14.840153	-4.910665	0.521704
H	16.127729	-4.736235	-0.708965
H	14.903968	-3.459964	-0.532992
Cl	14.199246	-3.496349	2.681452
C	19.231838	2.490539	0.475697
C	18.529745	3.725775	0.433157
C	18.627003	4.592516	1.601098
C	19.173119	4.047956	2.835701
H	17.818546	6.387729	0.744773
H	19.443138	2.033416	-0.48179
C	18.192038	5.893891	1.62289
C	19.263531	4.948932	3.885437
C	18.796631	6.227782	3.884819
C	18.259729	6.749667	2.696484
H	19.647806	4.581854	4.818094
H	19.007292	6.902314	4.732405
H	17.919672	7.806559	2.629148
C	19.596798	2.633606	2.939218
H	20.223553	2.41475	3.85761
H	18.597301	2.061966	3.035008
C	20.190664	2.210868	1.600905
H	20.262415	1.118374	1.716282
H	21.139757	2.763832	1.471517
C	17.826956	4.18186	-0.870287
H	17.600248	5.237702	-0.892438
H	18.598969	3.930634	-1.637529
C	16.643036	3.250111	-1.207189
H	16.791246	2.214848	-1.279496
H	16.182789	3.713537	-2.123136
C	15.53686	3.398358	-0.170173
O	14.327329	3.393518	-0.340212
O	16.065516	3.640478	1.079792
H	15.400348	3.737115	1.807679

Table S6: Helmholtz free energy of activation (in kcal/mol) and the lifetime of the halonium intermediate determined based on bond length analysis for the separate runs.

Run	ΔF‡ (kcal/mol)	Lifetime halonium intermediate (fs)	Total simulation time before TS (fs)
1	34	6486	74422
2	34	8294	78348

Entry 3: NBS *syn*-halolactonization in CH_2Cl_2

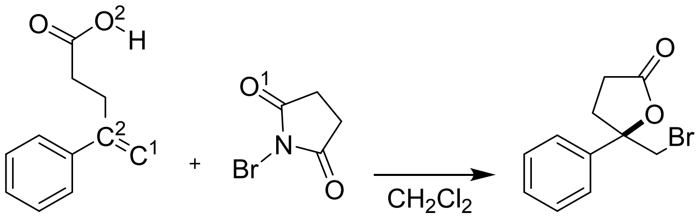

Table S7: Settings for the metadynamics simulation for the abovementioned reaction. [a] A half harmonic bias potential was added to CN1 at a value of 0.015 and force constant K = 300 a.u. to reduce the sampling region to a meaningful chemical space.

reactants	Box edge (Å)	CN1 / r0 [a]	CN2 / r0
1 x alkenoic acid<br>1 x NBS<br>29 x CH_2Cl_2	15.0	C^1-Br / 2.5 Å	C^2-O^2 / 1.9 Å

Topology of transition state excluding solvent molecules

C	10.010629	0.921513	14.076972
C	9.945552	2.923174	12.903248
N	10.382158	1.614217	12.946031
O	10.419157	-0.252956	14.309949
O	10.208119	3.747956	12.053883
H	14.377793	1.424997	11.568297
C	15.347843	0.935595	11.516869
C	17.819338	-0.454203	11.104383
C	15.407129	-0.423391	11.191663
C	16.576902	1.579417	11.607794
C	17.747114	0.841880	11.569094
C	16.626783	-1.100964	10.967141
H	16.540201	2.656729	11.611234
H	18.599678	1.425102	11.864114
H	16.558746	-2.109410	10.614239
H	18.699375	-1.080817	11.050633
C	14.104692	-1.331213	11.054960
C	13.124727	-0.821502	10.286318
H	12.251283	-1.416406	10.212560
C	14.153594	-2.656552	11.770816
H	13.243818	-3.145060	11.578763
H	14.938771	-3.272741	11.294820
C	14.424327	-2.634080	13.263906
H	15.245993	-2.008772	13.611380
H	14.658270	-3.676096	13.683686
C	13.247099	-2.069678	13.992228
O	13.253254	-1.726053	15.143333

O	12.231215	-1.823319	13.160327
H	11.484710	-1.220579	13.572032
H	13.469724	-0.130740	9.486257
Br	11.659394	0.733200	11.587771
C	9.056952	1.780074	14.844459
H	8.136647	1.255845	14.803576
H	9.387035	1.795273	15.887655
C	8.996372	3.113264	14.105320
H	7.992686	3.353096	13.727674
H	9.289962	4.037139	14.707422

Table S8: Helmholtz free energy of activation (in kcal/mol) and the lifetime of the halonium intermediate determined based on bond length analysis for the separate runs.

Run	ΔF‡ (kcal/mol)	Lifetime halonium intermediate (fs)	Total simulation time before TS (fs)
1	39	922	174905
2	26	688	52390
3	25	1219	73460

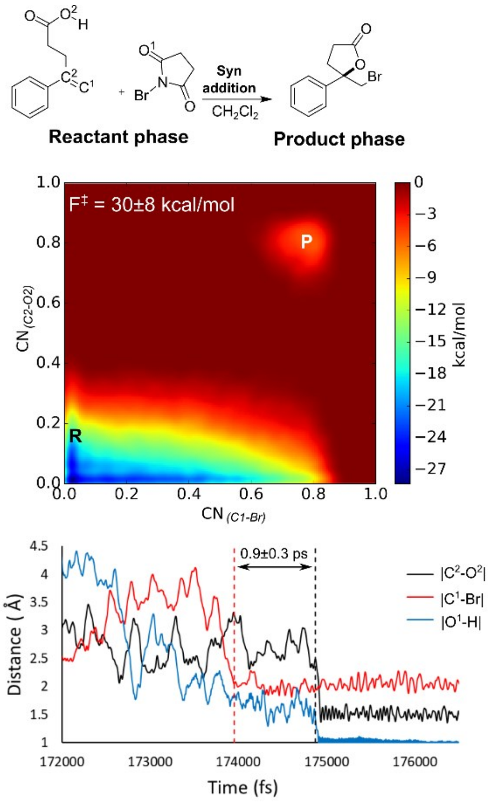

Figure S1. Analysis of the MtD simulations for the syn-mechanism of the NBS bromolactonization mentioned above. At the top a schematic representation of the reaction is provided. The free energy surface of the corresponding reaction is given as an average over three independent MtD simulations, together with the average Helmholtz free energy of activation ΔF‡, reactant phase (R) and product phase (P). Bond length analysis of a single simulation is provided below.

Entry 4: DCDMH *syn*-halolactonization in CH_2Cl_2

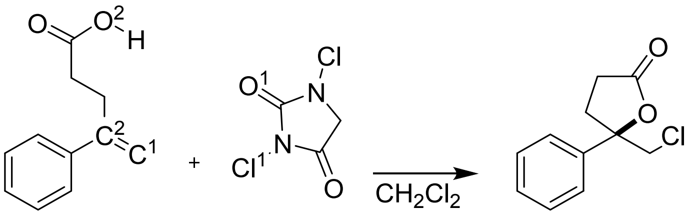

Table S9: Settings for the metadynamics simulation for the abovementioned reaction. [a] A half harmonic bias potential was added to CN1 at a value of 0.015 and force constant K = 300 a.u. to reduce the sampling region to a meaningful chemical space.

reactants	Box edge (Å)	CN1 / r0 [a]	CN2 / r0
1 x alkenoic acid<br>1 x DCDMH<br>29 x CH_2Cl_2	15.0	C^1-Cl^1 / 2.5 Å	C^2-O^2 / 1.9 Å

Table S10: Helmholtz free energy of activation (in kcal/mol) and the lifetime of the halonium intermediate determined based on bond length analysis for the separate runs.

Run	ΔF‡ (kcal/mol)	Lifetime halonium intermediate (fs)	Total simulation time before TS (fs)
1	20	604	28592
2	29	1618	35840
3	29	186	44335

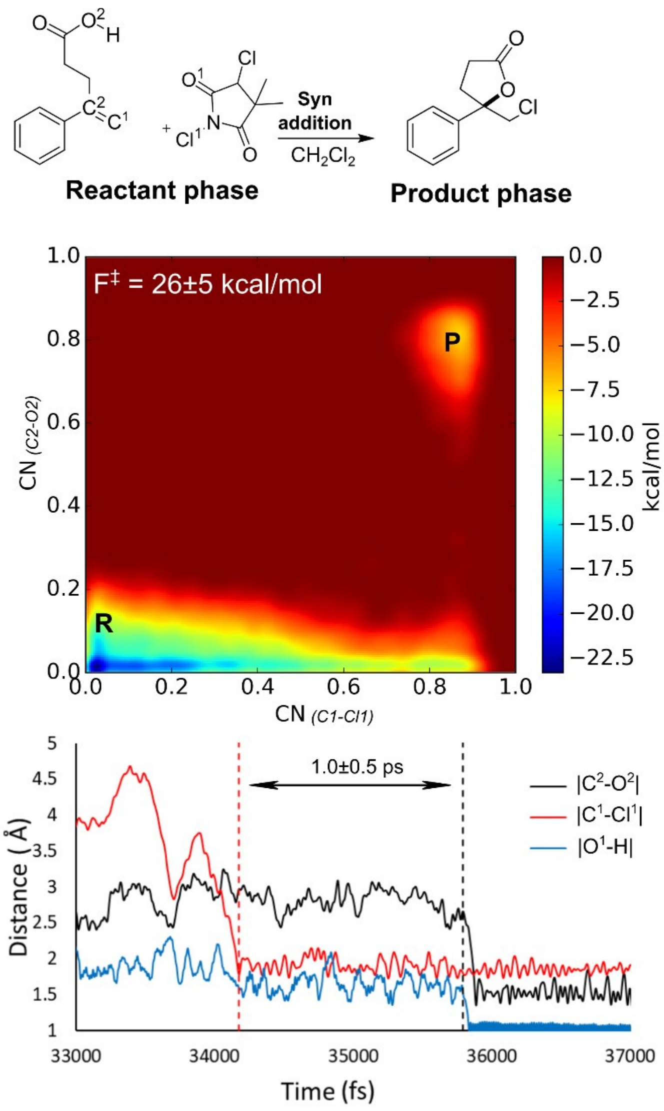

Figure S2. Analysis of the MtD simulations for the syn-mechanism of the DCDMH chlorolactonization mentioned above. At the top a schematic representation the reaction is provided. The free energy surface of the corresponding reaction is given as an average over three independent MtD simulations, together with the average Helmholtz free energy of activation ΔF‡, reactant phase (R) and product phase (P). Bond length analysis of a single simulation is provided below.

Entry 5: DCDMH *anti*-halolactonization in CH_2Cl_2 with quinuclidine as a base

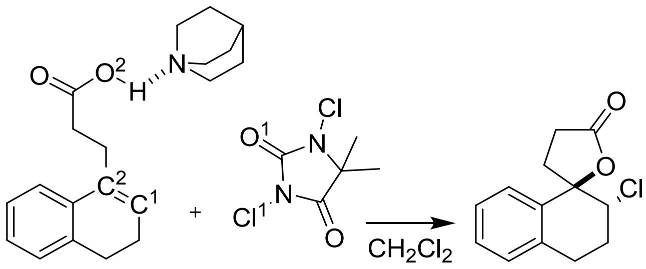

Table S11: Settings for the metadynamics simulation for the abovementioned reaction. [a] A half harmonic bias potential was added to CN1 and CN2 both at a value of 0.015 and force constant K = 300 a.u. to reduce the sampling region to a meaningful chemical space.

reactants	Box edge (Å)	CN1 / r0 [a]	CN2 / r0 [a]
1 x alkenoic acid<br>1 x DCDMH<br>1 x quinuclidine<br>27 x CH_2Cl_2	15.0	C^1-Cl^1 / 2.5 Å	N-H / 1.3 Å

Topology of transition state excluding solvent molecules

C	3.654769	-4.206612	-2.977678
C	3.471807	-2.948360	-2.298817
C	2.347045	-2.077580	-2.608652
C	1.615221	-2.369763	-3.747221
C	2.061376	-3.457607	-4.548721
C	2.991861	-4.424707	-4.170815
H	4.311522	-4.988236	-2.568965
H	3.987900	-2.987180	-1.367916
C	1.931058	-1.031251	-1.683252
H	1.519380	-3.678937	-5.522803
H	2.938917	-5.416522	-4.655275
C	0.697906	-0.322316	-1.878165
H	0.276311	0.134340	-1.024847
C	0.532927	-1.406319	-4.214441
H	1.043070	-0.605537	-4.793241
H	-0.243632	-1.899924	-4.801260
C	-0.213294	-0.794340	-2.955753
H	-0.807232	-1.589746	-2.513313
H	-0.906551	0.020541	-3.098688
C	2.829310	-0.725930	-0.489513
H	2.740986	-1.688598	0.116014
H	3.874882	-0.745349	-0.760940
C	2.476933	0.491990	0.384894
H	3.434990	0.903511	0.685732
H	2.010994	1.256034	-0.177815
C	1.683717	0.048282	1.639848
O	0.847592	0.892232	2.127681
O	1.867891	-1.190881	2.027394
H	0.782801	-1.730714	3.035926
C	0.877464	-2.002233	5.092211
C	-1.504911	-2.633909	5.762494
C	-0.114639	-2.174356	6.340036
H	1.634816	-2.789321	5.073552
H	1.540989	-1.121802	5.040223
H	0.197915	-3.087153	6.877389

H	-0.147660	-1.342929	7.017748
C	-0.394062	-3.417294	3.563013
H	-1.043807	-3.409367	2.655339
H	0.556487	-3.983328	3.412774
C	-1.303228	-3.885814	4.758528
H	-2.356232	-4.147245	4.392168
H	-0.820264	-4.638427	5.367035
H	-2.094750	-3.022116	6.539454
C	-2.137386	-1.453118	5.005647
H	-3.052038	-1.719379	4.537249
H	-2.253719	-0.559002	5.709911
C	-1.075268	-0.940522	3.892537
H	-1.505738	-0.921460	2.878400
H	-0.754579	0.055564	4.137429
N	0.083137	-1.967505	3.849924
N	2.245697	5.603293	-3.205774
C	2.104701	5.592777	-4.711610
C	2.212537	4.056863	-4.823082
C	2.698647	4.387703	-2.720113
N	2.483137	3.425868	-3.630496
Cl	1.911100	1.381754	-2.599787
O	1.863478	3.492133	-5.834777
O	3.230954	4.153215	-1.621429
C	0.644502	5.916056	-5.098836
H	-0.120107	5.379818	-4.471786
H	0.517780	5.571593	-6.170966
H	0.303053	6.997401	-4.963525
C	3.229442	6.470603	-5.451049
H	3.061090	6.333365	-6.493556
H	4.192118	6.019900	-5.189322
H	3.102924	7.514843	-5.168199
Cl	2.516484	7.140370	-2.346156

Table S12: Helmholtz free energy of activation (in kcal/mol) and the lifetime of the halonium intermediate determined based on bond length analysis for the separate runs.

Run	ΔF‡ (kcal/mol)	Lifetime halonium intermediate (fs)	Total simulation time before TS (fs)
1	9	3560	26950
2	6	10074	2800
3	11	17740	28308

Entry 6: NBS *anti*-halolactonization in CH_2Cl_2

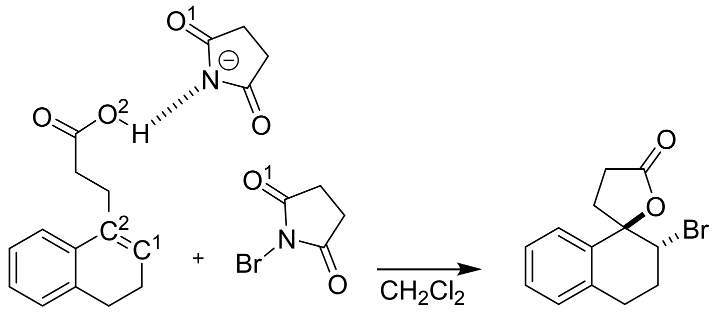

Table S13: Settings for the metadynamics simulation for the abovementioned reaction. [a] A half harmonic bias potential was added to CN1 and CN2 both at a value of 0.015 and force constant K = 300 a.u. to reduce the sampling region to a meaningful chemical space.

reactants	Box edge (Å)	CN1 / r0 [a]	CN2 / r0 [a]
1 x alkenoic acid<br>1 x NBS<br>1 x succ$^-$<br>28 x CH_2Cl_2	15.0	C^1-Br / 2.5 Å	N^--H / 1.3 Å

Topology of transition state excluding solvent molecules

C	-5.673811	2.964004	2.662465
C	-5.664358	1.373987	4.368021
N	-5.41294	2.677505	4.041007
O	-5.547505	4.104407	2.172837
O	-5.580177	0.855704	5.480292
C	-6.227406	1.628083	1.978458
H	-7.248556	1.781327	1.644698
H	-5.636866	1.481375	1.061062
C	-6.203501	0.599522	3.082952
H	-7.20508	0.26891	3.405696
H	-5.464505	-0.228425	2.964341
C	1.730032	3.68297	8.40674
C	0.588914	4.329679	7.905181
C	0.171073	4.174509	6.542701
C	0.906038	3.29967	5.640495
C	1.951714	2.641148	6.223519
C	2.421039	2.887055	7.52564
H	1.987324	3.69875	9.424129
H	-0.099584	4.875386	8.554328
C	-0.844724	5.128875	5.972532
H	2.433746	1.897007	5.635263
H	3.204561	2.171056	7.883925
C	-0.953277	5.166577	4.575128
H	-1.414188	6.033881	4.078849

Br	-3.070658	3.851625	4.363538
C	0.335535	3.057533	4.27943
H	1.003463	2.443584	3.626791
H	-0.515693	2.31423	4.373672
C	-0.052175	4.397228	3.645909
H	-0.398852	4.393626	2.646242
H	0.858697	5.068982	3.53406
C	-1.759242	5.989871	6.900066
H	-2.679024	5.366989	7.035144
H	-1.427644	6.054601	7.964224
C	-1.965218	7.412976	6.46552
H	-2.747698	7.504699	5.684209
H	-2.391549	8.025912	7.270466
C	-0.716224	8.175595	5.910124
O	0.347561	7.527646	5.893062
O	-0.741859	9.369723	5.514297
N	1.829252	10.981244	6.771888
C	2.234643	10.650183	8.080121
C	2.759377	11.734329	6.193703
O	1.808831	9.665268	8.707493
C	3.299368	11.689102	8.523812
O	2.85783	11.752344	4.968824
C	3.656462	12.337101	7.137987
H	2.744286	12.420192	9.243505
H	4.074053	11.237782	9.028433
H	3.357847	13.338786	7.216801
H	4.773241	12.300814	6.947462
H	1.014421	10.411419	6.286237

Table S14: Helmholtz free energy of activation (in kcal/mol) and the lifetime of the halonium intermediate determined based on bond length analysis for the separate runs.

Run	ΔF‡ (kcal/mol)	Lifetime halonium intermediate (fs)	Total simulation time before TS (fs)
1	12	0	16780
2	14	0	40536
3	14	0	41340

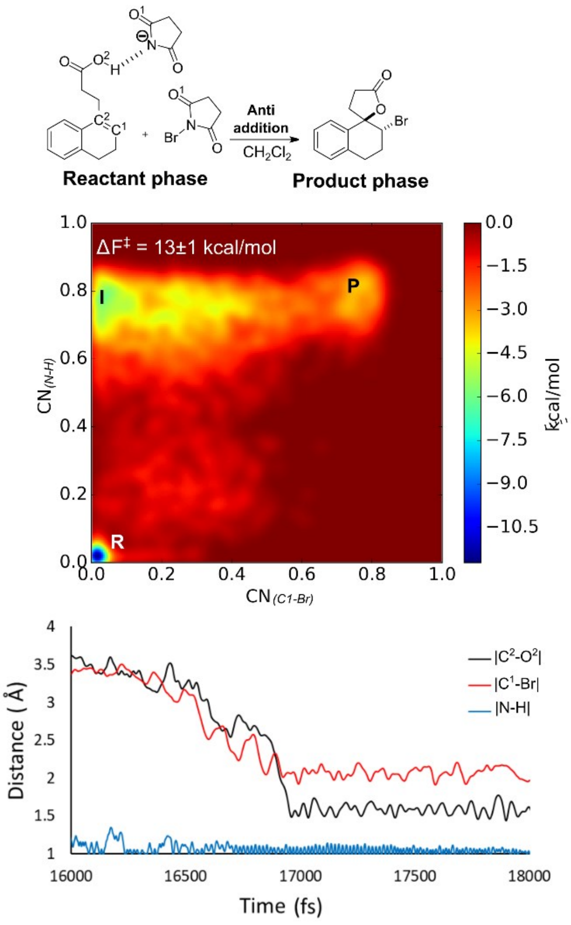

Figure S3: Analysis of the MtD simulations for the anti-mechanism of the NBS bromolactonization mentioned above. At the top a schematic representation of the reaction is provided. The free energy surface of the corresponding reaction is given as an average over three independent MtD simulations, together with the average Helmholtz free energy of activation ΔF‡, reactant phase (R), intermediate phase (I) and product phase (P). Bond length analysis of a single simulation is provided below. Note that the intermediate phase here corresponds to the deprotonation of the alkenoic acid by the succinimide anion.

Entry 7: β-halocarbenium formation during the DCDMH halolactonization in MeOH

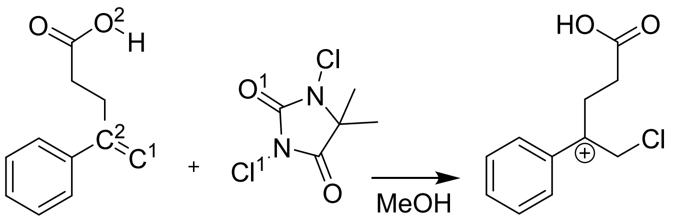

Table S15: Settings for the metadynamics simulation for the abovementioned reaction. [a] A half harmonic bias potential was added to CN1 at a value of 0.015 and force constant K = 300 a.u. to reduce the sampling region to a meaningful chemical space.

reactants	Box edge (Å)	CN1 / r0 [a]	CN2 / r0
1 x alkenoic acid	15.0	C^1-Cl^1 / 2.5 Å	C^2-O^2 / 1.9 Å
1 x DCDMH			
45 x MeOH			

1.3. Comparing the NBS *syn*-halolactonization of **1a** and **2**

Having established the *syn*-halolactonization pathways for **1a** and **2** in CH_2Cl_2, NCI analyses were performed on the transition state of the NBS bromolactonization reactions to further elucidate the similarities and differences in the mechanism caused by the substrate (Figure S4). As mentioned in the main article, an intermolecular hydrogen bond between NBS and the alkenoic acid directs the *syn*-addition in both cases. This hydrogen bond is visualized as a blue (attractive) isosurface between both reactants and marked by a purple arrow "a". Moreover, the nucleophilic oxygen of the carboxyl group (O^2) displays a noncovalent interaction with both the electrophilic bromine (attractive) and the carbons involved in the double bond (repulsive) for both reaction of **1a** and **2**. For this reason, it can again be concluded that the nucleophile forms a pre-polarized complex with the double bond. Although the mechanism of the *syn*-bromolactonization of **1a** and **2** in CH_2Cl_2 show significant similarities, there are some differences that need to be acknowledged. The 2D-plots of the reduced density gradient (*s*) show clear disparities between the number of peaks and their position. This indicates the transition states to contain different noncovalent interactions with different strengths depending on the substrate. As a particular example, the transition state for the NBS bromolactonization of **2** contains a 1,3-diaxial steric repulsion between the axial positioned hydrogen and the electrophilic bromine which is indicated by the purple arrow "b". For the open-chain substrate **1a**, this interaction is absent.

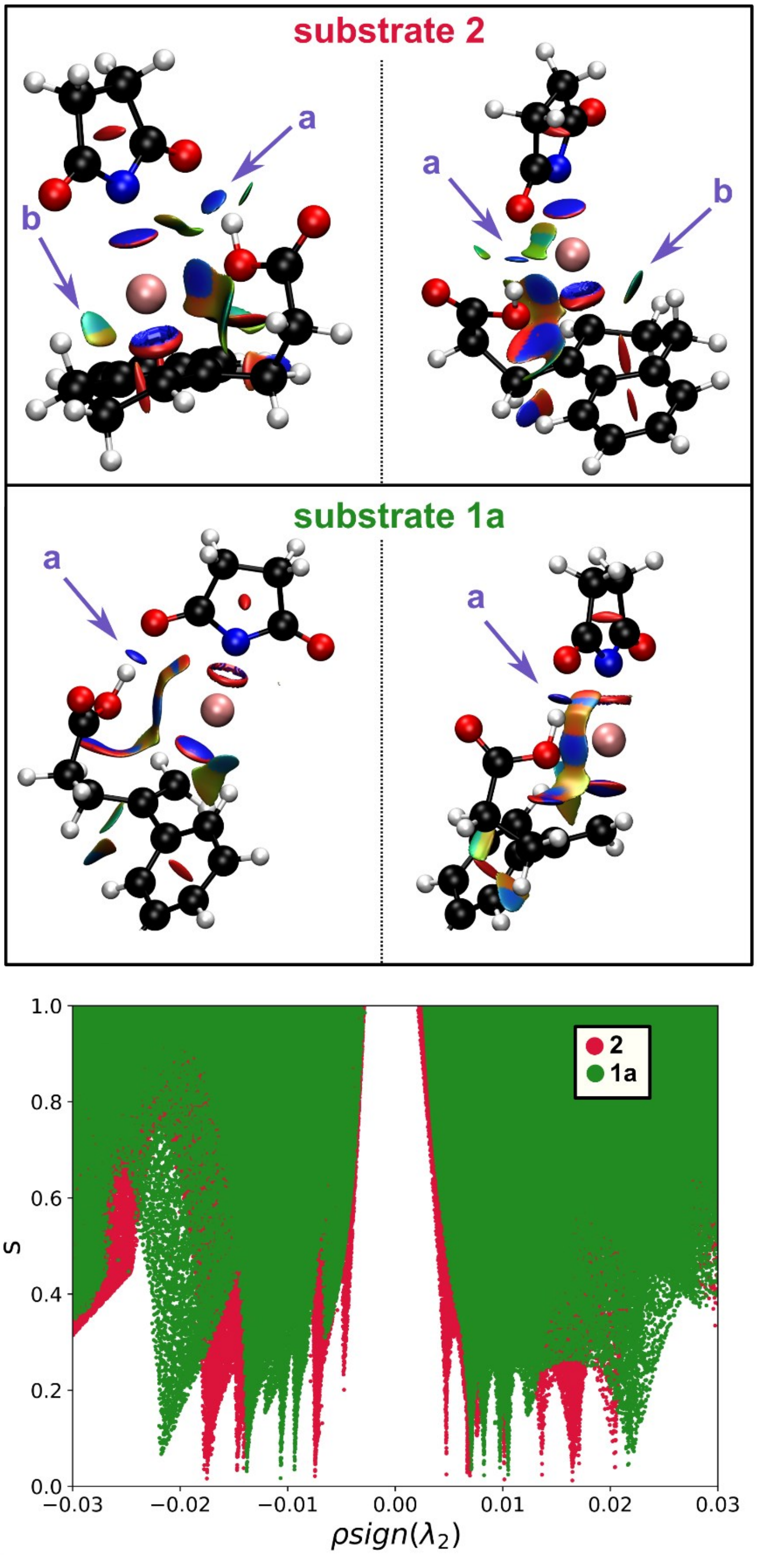

*Figure S4. 3D-NCI isosurfaces of the reduced density gradient (s = 0.5), indicating the noncovalent interactions present in the transition state of the NBS syn-halolactonization reaction of **1a** (green) and **2** (red) in CH_2Cl_2. The solvent is left out in the analysis for clarity. Isosurfaces are coloured with an RGB scale according to psign(λ_2) ranging from -0.015 a.u. to 0.015 a.u.*

1.4. Static transition states for the *anti*-halolactonization of 2

By including a second halogen source (DCDMH or NBS) to the structure, a plausible transition state for the *anti*-halolactonization of **2** is proposed. Frequency calculations using the 6-31G(d) basis set and the B3LYP[1] functional including Grimme's D3 dispersion correction[6] and the SMD implicit solvation model[4] to include dichloromethane as a solvent, validated the plausibility of the transition states as a single imaginary frequency was observed in both cases. Furthermore, IRC analysis showed that these transition states adequately connected the reactant to the product phase. Below, structures of these transition states are provided together with the value of their imaginary frequency and coordinates.

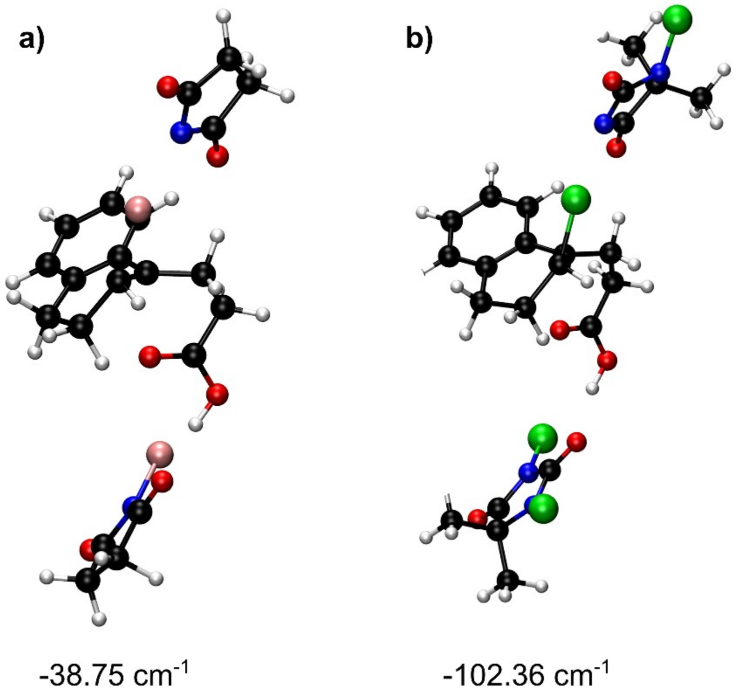

*Figure S5 Static DFT calculations were performed to suggest plausible transition states for the NBS (left) and DCDMH (right) anti-halolactonization of **2**. Both structures are characterized by a single imaginary frequency (provided at the bottom) connecting the reactant and product phase.*

*Coordinates for the transition state for the NBS anti-halolactonization of **2***

C	7.009000	-0.702000	-0.214000
C	5.399000	0.551000	-1.166000
N	5.648000	-0.364000	-0.201000
O	7.578000	-1.560000	0.488000
O	4.310000	1.067000	-1.449000
C	0.314000	1.015000	1.978000
C	1.517000	3.327000	0.919000
C	1.122000	0.925000	0.833000
C	0.049000	2.278000	2.557000
C	0.668000	3.417000	2.042000
C	1.744000	2.111000	0.337000

H	*-0.606000*	*2.364000*	*3.425000*
H	*0.500000*	*4.402000*	*2.508000*
H	*2.466000*	*1.994000*	*-0.486000*
H	*2.022000*	*4.223000*	*0.534000*
C	*1.376000*	*-0.386000*	*0.225000*
C	*1.640000*	*-1.532000*	*1.090000*
H	*1.517000*	*-2.526000*	*0.618000*
C	*1.522000*	*-0.503000*	*-1.296000*
H	*2.439000*	*0.038000*	*-1.599000*
H	*1.664000*	*-1.557000*	*-1.528000*
C	*0.292000*	*0.075000*	*-2.019000*
H	*0.303000*	*-0.196000*	*-3.080000*
H	*0.253000*	*1.175000*	*-1.950000*
C	*-0.957000*	*-0.445000*	*-1.347000*
O	*-0.949000*	*-0.767000*	*-0.126000*
O	*-2.057000*	*-0.490000*	*-2.117000*
H	*-2.808000*	*-0.883000*	*-1.592000*
C	*-7.379000*	*-1.590000*	*0.827000*
C	*-5.235000*	*-1.527000*	*-0.330000*
C	*-7.040000*	*-0.155000*	*0.342000*
N	*-5.781000*	*-0.220000*	*-0.318000*
O	*-4.125000*	*-1.822000*	*-0.778000*
O	*-7.695000*	*0.860000*	*0.540000*
C	*0.222000*	*-1.603000*	*1.961000*
H	*-0.693000*	*-1.881000*	*1.283000*
H	*0.254000*	*-2.387000*	*2.865000*
C	*-0.256000*	*-0.242000*	*2.688000*
H	*0.299000*	*-0.319000*	*3.691000*
H	*-1.379000*	*-0.165000*	*2.904000*
Br	*3.588000*	*-1.065000*	*0.765000*
Br	*-4.867000*	*1.258000*	*-1.023000*
C	*7.737000*	*0.107000*	*-1.273000*
H	*7.879000*	*-0.783000*	*-2.004000*
C	*6.684000*	*0.918000*	*-1.996000*
H	*8.767000*	*0.581000*	*-1.023000*
H	*6.811000*	*2.033000*	*-1.979000*
H	*6.540000*	*0.556000*	*-3.033000*
C	*-6.224000*	*-2.423000*	*0.246000*
H	*-6.719000*	*-2.498000*	*-0.885000*
H	*-5.805000*	*-3.371000*	*0.772000*
H	*-7.391000*	*-1.566000*	*1.939000*
H	*-8.352000*	*-1.921000*	*0.423000*

Coordinates for the transition state for the DCDMH anti-halolactonization of ***2***

C	*6.737000*	*0.341000*	*-1.205000*
N	*7.374000*	*-0.864000*	*-0.647000*
C	*6.707000*	*-1.277000*	*0.557000*
C	*5.369000*	*0.236000*	*-0.455000*
N	*5.452000*	*-0.650000*	*0.566000*
Cl	*3.147000*	*-1.309000*	*1.085000*
O	*7.160000*	*-2.062000*	*1.370000*
O	*4.395000*	*0.918000*	*-0.790000*
C	*0.022000*	*1.053000*	*2.220000*
C	*1.632000*	*3.293000*	*1.661000*
C	*0.980000*	*0.995000*	*1.192000*
C	*-0.163000*	*2.249000*	*2.934000*
C	*0.638000*	*3.364000*	*2.649000*
C	*1.795000*	*2.131000*	*0.928000*
H	*-0.917000*	*2.317000*	*3.715000*
H	*0.522000*	*4.283000*	*3.227000*
H	*2.598000*	*2.032000*	*0.194000*
H	*2.276000*	*4.150000*	*1.473000*

C	1.129000	-0.217000	0.428000
C	1.144000	-1.523000	1.113000
H	0.967000	-2.396000	0.482000
C	1.422000	-0.161000	-1.069000
H	2.413000	0.292000	-1.222000
H	1.449000	-1.173000	-1.454000
C	0.337000	0.666000	-1.800000
H	0.424000	0.535000	-2.889000
H	0.441000	1.740000	-1.592000
C	-1.042000	0.264000	-1.320000
O	-1.215000	-0.160000	-0.154000
C	7.399000	1.679000	-0.792000
H	6.757000	2.509000	-1.101000
H	8.382000	1.785000	-1.285000
H	7.538000	1.732000	0.299000
C	6.558000	0.239000	-2.729000
H	6.107000	-0.711000	-3.001000
H	7.530000	0.309000	-3.236000
H	5.908000	1.057000	-3.072000
Cl	9.115000	-0.950000	-0.670000
O	-2.027000	0.404000	-2.227000
H	-2.872000	0.055000	-1.842000
C	-7.566000	-0.569000	0.186000
N	-6.428000	-1.329000	-0.403000
C	-5.454000	-0.543000	-0.946000
C	-7.258000	0.860000	-0.329000
N	-5.984000	0.779000	-0.922000
O	-4.367000	-0.871000	-1.393000
O	-7.946000	1.854000	-0.231000
C	-8.927000	-1.077000	-0.375000
H	-9.713000	-0.375000	-0.075000
H	-9.156000	-2.059000	0.042000
H	-8.899000	-1.134000	-1.479000
C	-7.535000	-0.566000	1.737000
H	-6.567000	-0.174000	2.112000
H	-7.667000	-1.564000	2.109000
H	-8.347000	0.070000	2.104000
Cl	-6.064000	-2.963000	0.051000
Cl	-5.182000	2.086000	-1.683000
C	-0.372000	-1.519000	1.820000
H	-1.218000	-1.615000	0.997000
H	-0.532000	-2.386000	2.612000
C	-0.774000	-0.205000	2.653000
H	-0.371000	-0.468000	3.699000
H	-1.897000	-0.003000	2.748000

2. Synthetic procedures and characterization

2.1. General information

All reactions involving air- or moisture-sensitive reagents or intermediates were carried out in dried glassware under an argon or nitrogen atmosphere using standard *Schlenk* techniques. Diethyl ether and petroleum ether for flash chromatography were distilled before use. Dichloromethane and methanol for reactions were degassed, dried using activated alumina and stored over molecular sieves (3 Å) under argon. Toluene (99.85%, Extra Dry over Molecular Sieve, *AcroSeal™*) was purchased from *Acros Organics* and used as received. Unless otherwise stated, all commercially available chemicals were used without further purification. NBS was purified by crystallization from hot water, filtered and dried in a desiccator. Flash chromatography was carried out using *Acros* silica gel (0.035 - 0.070 mm; 60 Å) with pressure of about 1.1-1.5 bar. Thin layer chromatography (TLC) was carried out on *Merck* silica gel 60 F254 plates; compounds were visualized with UV light or by staining with a solution of $KMnO_4$ (13.1 g K_2CO_3, 0.20 g KOH, 2.00 g $KMnO_4$, 200 mL H_2O) followed by heating. Melting points were determined by using a *Büchi B-540* melting point apparatus. IR spectra were recorded on a *Thermo Scientific Nicolet 6700 FTIR*. IR signals are reported as *w* (weak), *m* (middle), *s* (strong) or *br* (broad) in cm^{-1}. NMR spectra were recorded on a *Bruker Avance 250* (250 MHz) or *Bruker Avance II 500* (500 MHz). ^{1}H-NMR and ^{13}C-NMR chemical shifts (δ) are reported in *ppm* relative to TMS and referenced to the residual solvent signal ($CDCl_3$: 7.26 ppm/77.0 ppm).[7] Mass spectrometry data of volatile compounds were recorded on a *Shimadzu GCMS-QP5050A* with electron ionization (EI). High-resolution mass spectrometry (HRMS) data were recorded on a *Micromass QTOF-micro* with electrospray ionization (ESI) and calibrated with Reserpin (2.10 – 3.00 mg/mL in 1:1 water/acetonitrile). Diastereomeric ratios of **6a** were determined with an *Agilent 1100 series* HPLC with UV detector (λ = 214 nm) using a chiral stationary phase (*Daicel CHIRALPAK IA* column, 0.46 cm × 25 cm). Heptane and EtOH (HPLC grade) were used as eluents with a flowrate of 1 mL/min.

2.2. Synthesis of starting materials

3-(3,4-dihydronaphthalen-1-yl) propanoic acid (**2**)[8] and 3-(1H-inden-3-yl) propanoic acid (**3**)[9] were prepared according to literature procedures.

Analytical data for 3-(3,4-dihydronaphthalen-1-yl) propanoic acid (2)[8]: **^{1}H-NMR** (250 MHz, $CDCl_3$, 298 K): δ (ppm) = 11.09 (*br* s, 1H), 7.28 – 7.09 (m, 4H, ArH), 5.91 (t, $^3J_{H,H}$ = 4.6 Hz, 1H, C=CH), 2.86 – 2.69 (m, 4H), 2.61 (dd, $^3J_{H,H}$ = 9.0 Hz, $^3J_{H,H}$ = 5.8 Hz, 2H), 2.32 – 2.17 (m, 2H). **^{13}C{^{1}H}-NMR** (63 MHz, $CDCl_3$, 298 K): δ (ppm) = 180.0 (COOH), 136.9 (C_q), 134.8 (C_q), 134.3 (C_q), 127.9 (CH), 127.0 (CH), 126.6 (CH), 125.7 (CH), 122.4 (CH), 33.3 (CH_2), 28.4 (CH_2), 27.6 (CH_2), 23.2 (CH_2).

Analytical data for 3-(1H-inden-3-yl)propanoic acid (3)[9]: **^{1}H-NMR** (250 MHz, $CDCl_3$, 299 K): δ (ppm) = 11.14 (*br* s, 1H, COOH), 7.44 (d, $^3J_{H,H}$ = 7.2 Hz, 1H, ArH), 7.38 – 7.25 (m, 2H, ArH), 7.24 – 7.16 (m, 1H, ArH), 6.22 (s, 1H, C=CH), 3.31 (s, 2H, CH_2), 2.95 – 2.82 (m, 2H, CH_2), 2.81 – 2.71 (m, 2H, CH_2). **^{13}C{^{1}H}-NMR** (63 MHz, $CDCl_3$, 299 K): δ (ppm) = 179.8 (COOH), 144.9 (C_q), 144.5 (C_q), 142.6 (C_q), 128.3 (CH), 126.3 (CH), 125.0 (CH), 124.0 (CH), 118.9 (CH), 37.9 (CH_2), 32.6 (CH_2), 22.8 (CH_2).

Synthesis of (*E*)-4-phenylhex-4-enoic acid (1b)

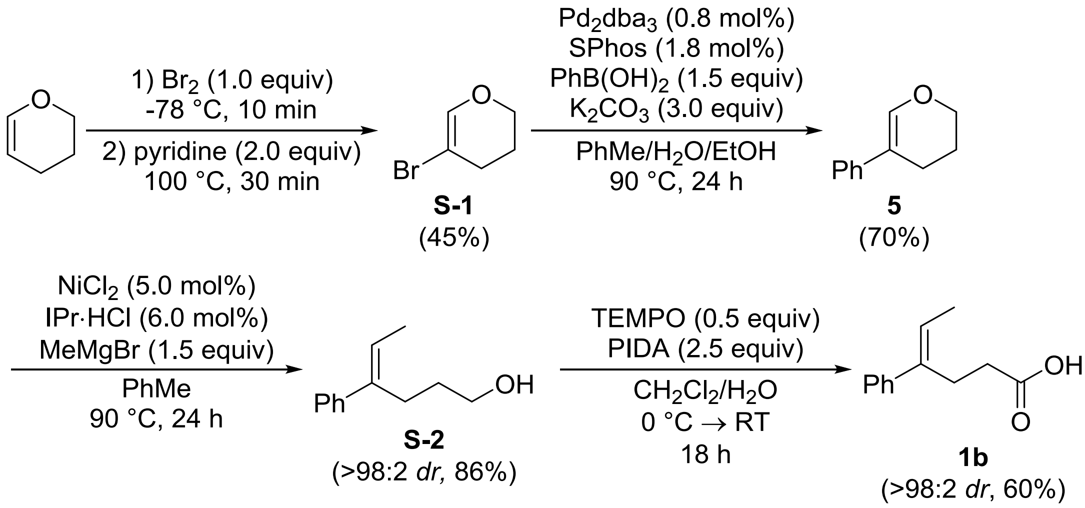

5-bromo-3,4-dihydro-2H-pyran (S-1):

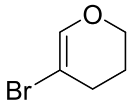

Based on a literature procedure[10], an oven dried 100 mL *Schlenk* round bottom flask with magnetic stir bar was charged with 3,4-dihydropyrane (4.6 mL, 51 mmol, 1.0 equiv). Bromine (2.6 mL, 51 mmol, 1.0 equiv) was added dropwise at -78 °C. Pyridine (8.2 mL, 102 mmol, 2.0 equiv) was added carefully and the reaction mixture was gradually heated to 100 °C over 30 minutes. After cooling to RT, aqueous hydrochloric acid (1 M, 125 mL) was added and the resulting mixture was extracted with pentane (3 x 100 mL). The combined organic phases were washed

with saturated, aqueous NaCl solution, dried with $MgSO_4$ and filtered. The solvents were removed *in vacuo* and the resulting crude was distilled (15 mbar, 60 °C). Product **S-1** was isolated as colourless liquid (3.7 g, 23 mmol, 45%).

^{1}H-NMR (500 MHz, $CDCl_3$, 300 K): δ (ppm) = 6.65 – 6.63 (m, 1H, C=CH), 3.98 – 3.93 (m, 2H, CH_2), 2.42 – 2.37 (m, 2H, CH_2), 2.03 – 1.95 (m, 2H, CH_2). **^{13}C{^{1}H}-NMR** (126 MHz, $CDCl_3$, 300 K): δ (ppm) = 142.9 (CH), 98.6 (CBr), 64.6 (CH_2), 29.0 (CH_2), 23.5 (CH_2). **GC-MS**: m/z = 162.0 calcd. for C_5H_7BrO [$M^\bullet$]; found: 161.9. Consistent with published data.[10]

5-phenyl-3,4-dihydro-2H-pyran (5):

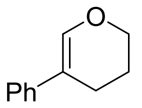

A 100 mL *Schlenk* tube with magnetic stir bar was charged with **S-1** (1.4 mL, 13 mmol, 1.0 equiv), phenyl boronic acid (2.4 g, 20 mmol, 1.5 equiv), K_2CO_3 (5.4 g, 40 mmol, 3.0 equiv), $Pd_2dba{\cdot}CHCl_3$ (103 mg, 0.10 mmol, 0.8 mol%) and SPhos (98 mg, 0.24 mmol, 1.8 mol%), evacuated and backfilled with argon. Toluene (18 mL), water (4 mL) and EtOH (4 mL) were added via a septum, which was then replaced by a screw cap with PTFE liner. The reddish reaction mixture was heated to 90 °C for 24 h and cooled to RT. Solids were removed by filtering through a sintered glass funnel containing silica gel which was washed thoroughly with EtOAc. The solvents were removed *in vacuo* and the resulting crude was purified by flash column chromatography (SiO_2, Et_2O in pentane: 0% → 2%). Product **5** was isolated as colourless oil (1.47 g, 9.19 mmol, 70%).

^{1}H-NMR (500 MHz, $CDCl_3$, 300 K): δ (ppm) = 7.34 – 7.26 (m, 4H, ArH), 7.22 – 7.14 (m, 1H, ArH), 6.96 – 6.92 (m, 1H, C=CH), 4.07 – 4.01 (m, 2H, CH_2), 2.46 – 2.40 (m, 2H, CH_2), 2.05 – 2.00 (m, 2H, CH_2). **^{13}C{^{1}H}-NMR** (126 MHz, $CDCl_3$, 300 K): δ (ppm) = 142.3 (C=CH), 139.8 (C_q), 128.5 (ArH), 125.9 (ArH), 124.2 (ArH), 113.1 (C_q), 65.7 (CH_2), 22.6 (CH_2), 22.5 (CH_2). **GC-MS**: m/z = 160.1 calcd. for $C_{11}H_{12}O$ [$M^\bullet$]; found: 160.1.

Consistent with published data.[11]

(*E*)-4-phenylhex-4-en-1-ol (S-2):

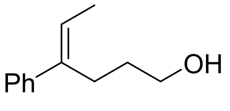

Based on a literature procedure[12], an oven dried 100 mL *Schlenk* tube was charged with $NiCl_2$ (59 mg, 0.49 mmol, 5.0 mol%) and 1,3-bis-(2,6-diisopropylphenyl) imidazolinium chloride (IPr·HCl; 234 mg, 0.55 mmol, 6.0 mol%), evacuated and backfilled with argon. Dry Toluene (2 mL) and methylmagnesium bromide (3 M in Et_2O; 1.53 mL, 4.59 mmol, 0.5 equiv) were added through a septum and the resulting mixture was stirred at RT for 30 min. Additional toluene (26 mL), methylmagnesium bromide (3.06 mL, 9.19 mmol, 1.0 equiv) and **1b''** (1.47 g, 9.19 mmol, 1.0 equiv) were added. The septum was replaced by a screw cap with PTFE liner and the reaction mixture was heated to 90 °C for 24 h. After cooling to RT, saturated, aqueous ammonium chloride solution (30 mL) was added and extracted with Et_2O (3 x 50 mL). The combined

organic phases were washed with saturated, aqueous NaCl solution, dried with $MgSO_4$ and filtered. The solvents were removed *in vacuo* and the resulting crude was purified by flash column chromatography (SiO_2, 10% EtOAc in PE). Product **S-2** was isolated as colourless oil (1.38 g, 7.87 mmol, 86%, brsm: 94%).

IR (neat): 3323*br*, 2932*br*, 1597*w*, 1441*m*, 1057*s*, 987*m*, 831*m*, 753*s*, 695*s*. **^{1}H-NMR** (500 MHz, $CDCl_3$, 300 K): δ (ppm) = 7.36 – 7.33 (m, 2H, ArH), 7.30 (dd, $^3J_{H,H}$ = 8.4 Hz, $^3J_{H,H}$ = 6.8 Hz, 2H, ArH), 7.24 – 7.20 (m, 1H, ArH), 5.79 (q, $^3J_{H,H}$ = 6.9 Hz, 1H, C=CH), 3.61 (t, $^3J_{H,H}$ = 6.5 Hz, 2H, CH_2), 2.61 (dd, $^3J_{H,H}$ = 8.5 Hz, $^3J_{H,H}$ = 6.7 Hz, 2H, CH_2), 1.82 (d, $^3J_{H,H}$ = 6.9 Hz, 3H, CH_3), 1.66 – 1.60 (m, 2H, CH_2), 1.40 (*br* s, 1H, OH). **^{13}C{^{1}H}-NMR** (126 MHz, $CDCl_3$, 300 K): δ (ppm) = 143.2 (C_q), 140.3 (C_q), 128.4 (CH), 126.7 (CH), 126.4 (CH), 123.5 (C=CH), 62.7 (CH_2), 31.4 (CH_2), 25.6 (CH_2), 13.8 (CH_3). **GC-MS**: *m/z* = 176.1 calcd. for $C_{12}H_{16}O$ [$M^•$]; found: 176.2.

Consistent with published data.[13]

(*E*)-4-phenylhex-4-enoic acid (1b):

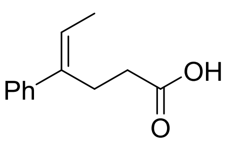

Based on a literature procedure[14], to a solution of **S-2** (1.00 g, 5.68 mmol, 1.0 equiv) in CH_2Cl_2 (12 mL) and water (6 mL), TEMPO (443 mg, 2.84 mmol, 0.5 equiv) and PIDA (4.57 g, 14.2 mmol, 2.5 equiv) were added at 0 °C. The reaction mixture was allowed to reach RT and stirred for 18 h. The reaction was stopped by adding saturated, aqueous $Na_2S_2O_3$ (10 mL) solution and extracted with CH_2Cl_2 (3 x 15 mL). The combined organic phases were dried with $MgSO_4$ and filtered. The solvents were removed *in vacuo* and the resulting crude was purified by flash column chromatography (SiO_2, CH_2Cl_2) followed by recrystallization from hot petroleum ether. Product **1b** was isolated as colourless solid (649 mg, 3.42 mmol, 60%).

MP: 65 °C. **IR** (neat): 3053*br*, 1703*s*, 1410*m*, 1284*m*, 1211*m*, 757*m*, 697*m*. **^{1}H-NMR** (500 MHz, $CDCl_3$, 298 K): δ (ppm) = 7.34 – 7.28 (m, 4H, ArH), 7.25 – 7.22 (m, 1H, ArH), 5.81 (q, $^3J_{H,H}$ = 6.9 Hz, 1H, C=CH), 2.90 – 2.85 (m, 2H, CH_2), 2.41 – 2.36 (m, 2H, CH_2), 1.84 (d, $^3J_{H,H}$ = 6.9 Hz, 3H, CH_3). **^{13}C{^{1}H}-NMR** (126 MHz, $CDCl_3$, 299 K): δ (ppm) = 179.9 (COOH), 142.2 (C_q), 138.8 (C_q), 128.5 (ArH), 127.0 (ArH), 126.4 (ArH), 124.5 (C=CH), 33.0 (CH_2), 24.7 (CH_2), 14.2 (CH_3). **HRMS**: *m/z* = 213.0886 calcd. for $C_{12}H_{14}NaO_2^+$ [$M+Na^+$]; found: 213.0892.

Consistent with published data.[13]

2.3. Synthesis and characterization of halolactonization products

General procedure: An oven dried 15 mL *Schlenk* tube with a magnetic stir bar was charged with the respective starting material (0.10 mmol, 1.0 equiv) and additive (0.10 mmol, 1.0 equiv), evacuated and backfilled with argon. Dry solvent (2 mL, 0.05 M) was added and the solution was cooled to 0 °C with an immersion cooler using a *i*-PrOH bath. The respective halogenating agent (0.12 mmol, 1.2 equiv) was added and the tube was sealed with a screw cap with PTFE liner. After 24 h the reaction was stopped by adding saturated, aqueous $Na_2S_2O_3$ solution (2 mL) and the mixture was extracted with CH_2Cl_2 (3 x 10 mL). The combined organic phases were dried with $MgSO_4$ and filtered. The solvents were removed *in vacuo* and to the resulting crude mesitylene (15 µL, 0.11 mmol, 0.91 equiv) was added, redissolved in $CDCl_3$ (600 µL) and measured via ^{1}H-NMR. Products were subsequently purified by column chromatography.

Effects of substrates and reaction conditions on diastereoselectivity. All reactions were carried out following the general procedure using solvents and halogen sources given in the table.

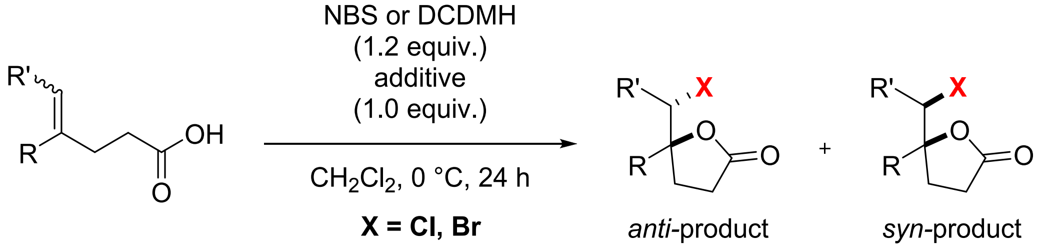

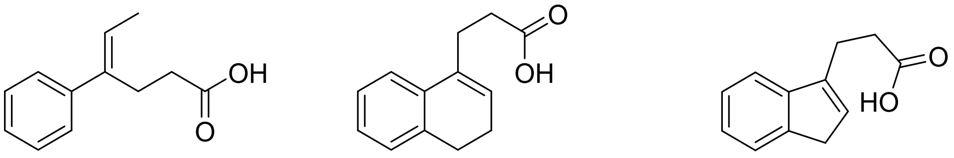

Table S16: Experimental results relating to the influence of substrates. [a] determined from crude mixture using NMR; [b] determined by NMR using mesitylene as internal standard; [c] determined by HPLC.

Entry	Substrate	Solvent	Halogen source	Additive (1.0 equiv)	*d.r.* [a] (*anti:syn*)	Yield [b]
**1**	**3**	CH_2Cl_2	NBS	-	71:29	**6b**, 49%
**2**	**3**	MeOH	NBS	-	99:1	**6b**, 99%
**3**	**3**	CH_2Cl_2	DCDMH	-	60:40	**6a**, 69%
**4**	**3**	MeOH	DCDMH	-	99:1	**6a**, 99%
**5**	**2**	CH_2Cl_2	NBS	-	80:20	**7b**, 44%
**6**	**2**	MeOH	NBS	-	99:1	**7b**, 99%
**7**	**2**	CH_2Cl_2	DCDMH	-	76:24	**7a**, 90%
**8**	**2**	MeOH	DCDMH	-	99:1	**7a**, 99%
**9**	**1b**	CH_2Cl_2	NBS	-	99:1	**8b**, 10%
**10**	**1b**	MeOH	NBS	-	99:1	**8b**, 99%
**11**	**1b**	CH_2Cl_2	DCDMH	-	57:43 [c]	**8a**, 13%
**12**	**1b**	MeOH	DCDMH	-	99:1	**8a**, 60%
**13**	**3**	CH_2Cl_2	NBS	Quinuclidine	99:1	**6b**, 99%
**14**	**3**	CH_2Cl_2	DCDMH	Quinuclidine	93:7	**6a**, 92%
**15**	**2**	CH_2Cl_2	NBS	Quinuclidine	99:1	**7b**, 99%
**16**	**2**	CH_2Cl_2	DCDMH	Quinuclidine	99:1	**7a**, 99%
**17**	**1b**	CH_2Cl_2	NBS	Quinuclidine	99:1	**8b**, 99%
**18**	**1b**	CH_2Cl_2	DCDMH	Quinuclidine	99:1	**8a**, 99%

Effects of substrates and reaction conditions on diastereoselectivity. All reactions were carried out following the general procedure using solvents and halogen sources given in the table.

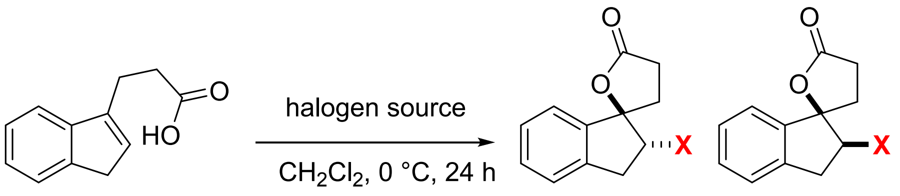

3
X = F, Cl, Br, I

anti-**6** *anti*-product

syn-**6** *syn*-product

Table S17: Experimental results relating to the influence of quantity and type of halogen source. [a] determined from crude mixture using NMR; [b] determined by NMR using mesitylene as internal standard.

**Entry**	**Halogen source**	**Equivalents**	***d.r.*[a] (*anti:syn*)**	**Yield [b]**
**1**	NBS	10	78:22	**6b**, 93%
**2**	NBS	5.0	75:25	**6b**, 89%
**3**	NBS	2.0	75:25	**6b**, 75%
**4**	NBS	1.2	71:29	**6b**, 49%
**5**	NBS	1.0	67:33	**6b**, 36%
**6**	NBS	0.75	65:35	**6b**, 31%
**7**	NBS	0.50	66:34	**6b**, 40%
**8**	DCDMH	10	63:37	**6a**, 83%
**9**	DCDMH	5.0	62:38	**6a**, 69%
**10**	DCDMH	2.0	61:39	**6a**, 77%
**11**	DCDMH	1.2	60:40	**6a**, 69%
**12**	DCDMH	1.0	60:40	**6a**, 57%
**13**	DCDMH	0.75	61:39	**6a**, 67%
**14**	DCDMH	0.50	59:41	**6a**, 60%
**15**	NIS	1.2	97:3	**6c**, 96%
**16**	DBDMH	1.2	69:31	**6b**, 99%
**17**	TBCO	1.2	97:3	**6b**, 98%
**18**	NCS	1.2	60:40	**6a**, <10%
**19**	*t*-BuOCl	1.2	76:24	**6a**, 61%
**20**	Selectfluor	1.2	-	0%
**21**	NFSI	1.2	-	0%

Analytical data for isolated reaction products of the halolactonisations of 1b, 2 and 3 using DCDMH, NBS or NIS, respectively.

***rac-anti*-6a:**

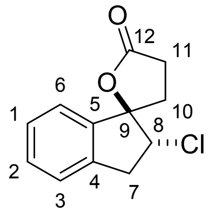

MP: 108 °C. **IR** (neat): 2952*br*, 1771*s*, 1462*w*, 1238*m*, 1145*m*, 1050*m*, 900*m*, 839*m*, 758*m*. **^{1}H-NMR** (500 MHz, $CDCl_3$, 299 K): δ (ppm) = 7.37 – 7.29 (m, 3H, H-1/H-2/H-6), 7.23 (d, $^3J_{H,H}$ = 6.5 Hz, 1H, H-3), 4.65 (dd, $^3J_{H,H}$ = 8.3 Hz, $^3J_{H,H}$ = 7.3 Hz, 1H), 3.48 (dd, $^2J_{H,H}$ = 15.5 Hz, $^3J_{H,H}$ = 7.3 Hz, 1H, H-7a), 3.09 (dd, $^2J_{H,H}$ = 15.7 Hz, $^3J_{H,H}$ = 8.3 Hz, 1H, H-7b), 2.99 – 2.91 (m, 1H, H-11a), 2.91 – 2.84 (m, 1H, H-10a), 2.76 – 2.69 (m, 1H, H-11b), 2.24 (ddd, $^2J_{H,H}$ = 13.2 Hz, $^3J_{H,H}$ = 10.0 Hz, $^3J_{H,H}$ = 8.3 Hz, 1H, H-10b). **^{13}C{^{1}H}-NMR** (126 MHz, $CDCl_3$, 300 K): δ (ppm) = 176.2 (C-12), 141.6 (C-5), 138.1 (C-4), 129.9 (C-2), 128.3 (C-1), 124.9 (C-3), 123.0 (C-6), 94.5 (C-9), 65.2 (C-8), 39.2 (C-7), 29.6 (C-10), 29.2 (C-11). **HRMS (ESI)**: *m/z* = 245.0340 calcd. for $C_{12}H_{11}ClNaO_2^+$ [M+Na$^+$]; found: 245.0431. **Crystal structure**: see page S41.

***rac-syn*-6a:**

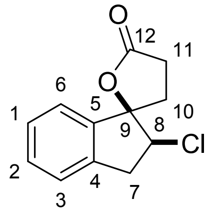

MP: 136 °C. **IR** (neat): 2955*br*, 1774*s*, 1462*w* 1265*m*, 1159*m*, 959*m*, 842*m*. **^{1}H-NMR** (500 MHz, $CDCl_3$, 299 K): δ (ppm) = 7.41 – 7.37 (m, 1H, H-2), 7.36 (d, $^3J_{H,H}$ = 8.0 Hz, 1H, H-6), 7.35 – 7.31 (m, 1H, H-1), 7.28 (d, $^3J_{H,H}$ = 7.4 Hz, 1H, H-3), 4.34 (dd, $^3J_{H,H}$ = 8.9 Hz, $^3J_{H,H}$ = 7.6 Hz, 1H, H-8), 3.37 – 3.28 (m, 2H, H-7), 3.02 – 2.91 (m, 1H, H-11a), 2.84 – 2.79 (m, 1H, H-11b), 2.78 – 2.74 (m, 1H, H-10a), 2.53 – 2.45 (m, 1H, H-10b). **^{13}C{^{1}H}-NMR** (126 MHz, $CDCl_3$, 300 K): δ (ppm) = 175.8 (C-12), 141.1 (C-4), 139.6 (C-5), 130.7 (C-2), 128.1 (C-1), 125.1 (C-3), 123.5 (C-6), 91.4 (C-9), 64.6 (C-8), 39.4 (C-7), 29.7 (C-11), 29.1 (C-10). **HRMS (ESI)**: *m/z* = 245.0340 calcd. for $C_{12}H_{11}ClNaO_2^+$ [M+Na$^+$]; found: 245.0383. **Crystal structure**: see page S42.

***rac-anti*-6b:**

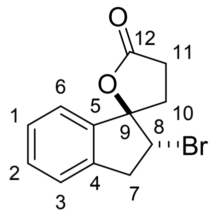

MP: 97 °C. **IR** (neat): 2952*br*, 1770*s*, 1460*w*, 1232*m*, 1142*m*, 1046*m*, 897*m*, 754*m*, 639*w*. **^{1}H-NMR** (500 MHz, $CDCl_3$, 299 K): δ (ppm) = 7.36 – 7.29 (m, 3H, H-1/H-2/H-6), 7.25 – 7.22 (m, 1H, H-3), 4.68 (dd, $^3J_{H,H}$ = 8.2 Hz, $^3J_{H,H}$ = 7.1 Hz, 1H, H-8), 3.54 (ddd, $^2J_{H,H}$ = 15.9 Hz, $^3J_{H,H}$ = 7.2 Hz, $^4J_{H,H}$ = 0.8 Hz, 1H, H-7a), 3.20 (dd, $^2J_{H,H}$ = 15.8 Hz, $^3J_{H,H}$ = 8.2 Hz, 1H, H-7b), 2.99 (ddd, $^2J_{H,H}$ = 17.7 Hz, $^3J_{H,H}$ = 10.1 Hz, $^3J_{H,H}$ = 8.7 Hz, 1H, H-11a), 2.88 (ddd, $^2J_{H,H}$ = 13.5 Hz, $^3J_{H,H}$ = 10.1 Hz, $^3J_{H,H}$ = 4.2 Hz, 1H, H-10a), 2.73 (ddd, $^2J_{H,H}$ = 17.7 Hz, $^3J_{H,H}$ = 10.1 Hz, $^3J_{H,H}$ = 4.2 Hz, 1H, H-10b), 2.32 (ddd, $^2J_{H,H}$ = 13.5 Hz, $^3J_{H,H}$ = 10.1 Hz, $^3J_{H,H}$ = 8.7 Hz, 1H, H-11b). **^{13}C{^{1}H}-NMR** (126 MHz, $CDCl_3$, 300 K): δ (ppm) = 176.2 (C-12), 141.7 (C-5), 139.1 (C-4), 129.9 (C-2/6), 128.3 (C-1), 124.8 (C-3), 123.0 (C-2/6), 94.3 (C-9), 56.0 (C-8), 40.1 (C-7), 31.2 (C-10), 29.3

(C-11). **HRMS (ESI)**: *m/z* = 288.9835 calcd. for $C_{12}H_{11}BrNaO_2^+$ [M+Na$^+$]; found: 288.9840. **Crystal structure**: see page S43. Consistent with published data.[15]

***rac-syn*-6b:**

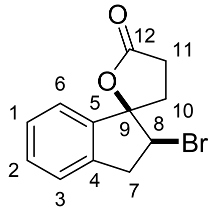

MP: 116 °C. **IR** (neat): 2954*br*, 1771*s*, 1461*w*, 1255*m*, 1168*m*, 1147*m*, 1055*w*, 955*m*, 729*w*. **^{1}H-NMR** (50 MHz, $CDCl_3$, 299 K): *δ* (ppm) = 7.41 – 7.35 (m, 2H, H-2/H-6), 7.36 – 7.29 (m, 1H, H-1), 7.28 (d, $^3J_{H,H}$ = 7.4 Hz, 1H), 4.37 (dd, $^3J_{H,H}$ = 9.1 Hz, $^3J_{H,H}$ = 7.6 Hz, 1H, H-8), 3.44 – 3.34 (m, 2H, H-7), 3.03 – 2.92 (m, 1H, H-11a), 2.88 – 2.80 (m, 1H, H-11b), 2.80 – 2.74 (m, 1H, H-10a), 2.52 – 2.43 (m, 1H, H-10b). **^{13}C{^{1}H}-NMR** (126 MHz, $CDCl_3$, 300 K): *δ* (ppm) = 175.6 (C-12), 142.0 (C-4), 139.8 (C-5), 130.7 (C-2), 128.1 (C-1), 124.9 (C-3), 123.4 (C-6), 91.4 (C-9), 55.3 (C-8), 40.2 (C-7), 29.9 (C-11), 29.2 (C-10). **HRMS (ESI)**: *m/z* = 288.9835 calcd. for $C_{12}H_{11}BrNaO_2^+$ [M+Na$^+$]; found: 288.9840. **Crystal structure**: see page S44.

***rac-anti*-6c:**

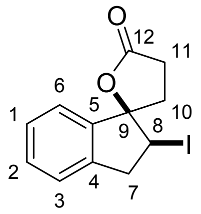

IR (neat): 2948*br*, 1770*s*, 1450*m*, 1228*s*, 1260*s*, 1042*m*, 1025*m*, 898*m*, 754*m*. **^{1}H-NMR** (500 MHz, $CDCl_3$, 298 K): *δ* (ppm) = 7.35 – 7.31 (m, 2H, H-6; H-2), 7.31 – 7.28 (m, 1H, H-1), 7.26 – 7.21 (m, 1H, H-3), 4.71 (dd, $^3J_{H,H}$ = 8.3 Hz, $^3J_{H,H}$ = 7.1 Hz, 1H, H-8), 3.58 (dd, $^2J_{H,H}$ = 16.0 Hz, $^3J_{H,H}$ = 7.1 Hz, 1H, H-7a), 3.28 (dd, $^2J_{H,H}$ = 16.0 Hz, $^3J_{H,H}$ = 8.3 Hz, 1H, H-7b), 3.10 – 3.00 (m, 1H, H-11a), 2.83 – 2.77 (m, 1H, H-10a), 2.77 – 2.70 (m, 1H, H-11b), 2.45 – 2.36 (m, 1H, H-10b). **^{13}C{^{1}H}-NMR** (126 MHz, $CDCl_3$, 298 K): *δ* (ppm) = 176.1 (C-12), 141.4 (C-5), 140.7 (C-4), 129.8 (C-2), 128.2 (C-1), 124.7 (C-3), 122.8 (C-6), 94.5 (C-9), 42.2 (C-7), 34.0 (C-10), 33.0 (C-8), 29.4 (C-11). **HRMS (ESI)**: *m/z* = 336.9696 calcd. for $C_{12}H_{11}INaO_2^+$ [M+Na$^+$]; found: 337.0950.

***rac-anti*-7a:**

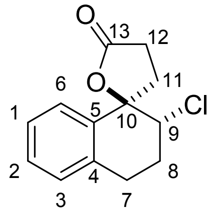

MP: 150 °C. **IR** (neat): 2947*br*, 1774*s*, 1455*w*, 1237*m*, 1197*m*, 1159*m*, 997*w*, 911*w*, 760*w*. **^{1}H-NMR** (500 MHz, $CDCl_3$, 299 K): *δ* (ppm) = 7.31 – 7.27 (m, 1H, H-6), 7.28 – 7.26 (m, 1H, H-1), 7.26 – 7.23 (m, 1H, H-2), 7.13 – 7.09 (m, 1H, H-3), 4.50 (dd, $^3J_{H,H}$ =12.2 Hz, $^3J_{H,H}$ = 3.5 Hz, 1H, H-9), 3.07 – 2.94 (m, 2H, H-7), 2.93 – 2.84 (m, 1H, H-12a), 2.83 – 2.75 (m, 2H, H-11a/H-12b), 2.49 – 2.42 (m, 1H, H-8a), 2.29 – 2.19 (m, 2H, H-8b/H-11b). **^{13}C{^{1}H}-NMR** (126 MHz, $CDCl_3$, 300 K): *δ* (ppm) = 176.8 (C-13), 138.6 (C-5), 134.3 (C-4), 128.8 (C-3), 128.5 (C-2), 127.3 (C-1), 126.0 (C-6), 87.5 (C-10), 64.4 (C-9), 32.2 (C-11), 29.9 (C-12), 29.9 (C-8), 28.4 (C-7). **HRMS (ESI)**: *m/z* = 259.0496 calcd. for $C_{13}H_{13}ClNaO_2^+$ [M+Na$^+$]; found: 259.0533. **Crystal structure**: see page S45.

***rac-syn*-7a**:

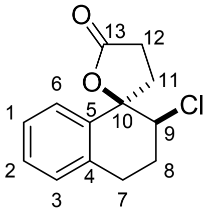

IR (neat): 2941*br*, 1770*s*, 1454*w*, 1189*s*, 1051*m*, 997*w*, 960*w*, 907*w*, 762*m*. **^{1}H-NMR** (500 MHz, $CDCl_3$, 299 K): *δ* (ppm) = 7.34 – 7.28 (m, 1H, H-6), 7.30 – 7.24 (m, 2H, H-1/H-2), 7.17 – 7.12 (m, 1H, H-3), 4.31 (dd, $^3J_{H,H}$ = 9.8 Hz, $^3J_{H,H}$ =3.1 Hz, 1H, H-9), 3.09 (dt, $^3J_{H,H}$ = 17.3 Hz, $^3J_{H,H}$ = 5.6 Hz, 1H, H-7a), 2.93 (ddd, $^2J_{H,H}$ = 17.4 Hz, $^3J_{H,H}$ = 8.9 Hz, $^3J_{H,H}$ =6.6 Hz, 1H, H-7a), 2.90 – 2.79 (m, 2H, H-12), 2.65 (ddd, $^2J_{H,H}$ = 13.8 Hz, $^3J_{H,H}$ = 9.7 Hz, $^3J_{H,H}$ = 7.8 Hz, 1H, H-11a), 2.56 (ddd, $^2J_{H,H}$ = 13.8 Hz, $^3J_{H,H}$ = 10.3 Hz, $^3J_{H,H}$ = 7.6 Hz, 1H, H-11b), 2.46 – 2.37 (m, 1H, H-8a), 2.32 (dtd, $^2J_{H,H}$ =14.0 Hz, $^3J_{H,H}$ = 5.8 Hz, $^3J_{H,H}$ = 3.1 Hz, 1H, H-8b). **^{13}C{^{1}H}-NMR** (126 MHz, $CDCl_3$, 300 K): *δ* (ppm) = 176.1 (C-13), 136.5 (C-5), 135.4 (C-4), 129.2 (C-3), 128.9 (C-2), 127.2 (C-1), 126.9 (C-6), 85.3 (C-10), 65.2 (C-9), 33.7 (C-11), 29.6 (C-12), 28.8 (C-8), 27.7 (C-7). **HRMS (ESI)**: *m/z* = 259.0496 calcd. for $C_{13}H_{13}ClNaO_2^+$ [M+Na$^+$]; found: 259.0528.

***rac-anti*-7b**:

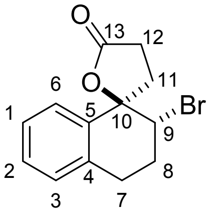

MP: 111 °C. **IR** (neat): 2955*br*, 1771*s*, 1454*m*, 1231*m*, 1182*s*, 1156*m*, 1040*m*, 910*m*, 763*m*. **^{1}H-NMR** (500 MHz, $CDCl_3$, 299 K): *δ* (ppm) = 7.29 – 7.26 (m, 1H, H-6), 7.26 – 7.22 (m, 2H, H-1/H-2), 7.13 – 7.09 (m, 1H, H-3), 4.60 (dd, $^3J_{H,H}$ = 12.1 Hz, $^3J_{H,H}$ = 3.5 Hz, 1H, H-9), 3.06 – 2.91 (m, 2H, H-7), 2.90 – 2.83 (m, 1H, H-12a), 2.83 – 2.74 (m, 2H, H-11a/H-12b), 2.59 – 2.53 (m, 1H, H-8a), 2.40 – 2.32 (m, 1H, H-8b), 2.32 – 2.25 (m, 1H, H-11b). **^{13}C{^{1}H}-NMR** (126 MHz, $CDCl_3$, 300 K): *δ* (ppm) = 176.5 (C-13), 138.3 (C-5), 134.1 (C-4), 128.8 (C-3), 128.5 (C-2), 127.2 (C-1), 125.7 (C-6), 86.8 (C-10), 57.6 (C-9), 33.2 (C-11), 30.4 (C-8), 29.8 (C-12), 29.1 (C-7). **HRMS (ESI)**: *m/z* = 302.9991 calcd. for $C_{13}H_{13}BrNaO_2^+$ [M+Na$^+$]; found: 302.9980. **Crystal structure**: see page S46.

***rac-syn*-7b**:

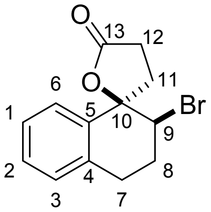

IR (neat): 2934*br*, 1774*s*, 1453*w*, 1182*m*, 1043*m*, 763*m*. **^{1}H-NMR** (500 MHz, $CDCl_3$, 299 K): *δ* (ppm) = 7.31 – 7.25 (m, 3H, H-1/H-2/H-6), 7.16 – 7.13 (m, 1H, H-3), 4.45 (dd, $^3J_{H,H}$ = 9.3 Hz, $^3J_{H,H}$ = 3.1 Hz, 1H, H-9), 3.09 (dt, $^2J_{H,H}$ = 17.3 Hz, $^3J_{H,H}$ = 5.9 Hz, 1H, H-7a), 2.94 (ddd, $^2J_{H,H}$ = 17.3 Hz, $^3J_{H,H}$ = 8.0 Hz, $^3J_{H,H}$ = 6.2 Hz, 1H, H-7b), 2.87 (ddd, $^3J_{H,H}$ = 9.7 Hz, $^3J_{H,H}$ = 7.3 Hz, $^3J_{H,H}$ = 5.1 Hz, 2H, H-12), 2.65 (ddd, $^2J_{H,H}$ = 13.7 Hz, $^3J_{H,H}$ = 9.8 Hz, $^3J_{H,H}$ = 7.2 Hz, 1H, H-11a), 2.59 – 2.52 (m, 1H, H-11b), 2.52 – 2.48 (m, 1H, H-8a), 2.47 – 2.40 (m, 1H, H-8b). **^{13}C{^{1}H}-NMR** (126 MHz, $CDCl_3$, 300 K): *δ* (ppm) = 175.9 (C-13), 136.6 (C-5), 135.1 (C-4), 129.2 (C-3), 128.9 (C-2/C-6), 127.2 (C-1), 126.8 (C-2/C-6), 85.2 (C-10), 58.1 (C-9), 34.8 (C-11), 29.7 (C-8/C-12), 29.6 (C-8/C-12), 28.4 (C-7). **HRMS (ESI)**: *m/z* = 302.9991 calcd. for $C_{13}H_{13}BrNaO_2^+$ [M+Na$^+$]; found: 303.0004.

***rac-anti*-8a**:

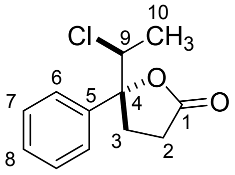

IR (neat): 2986*br*, 1775*s*, 1448*w*, 1194*m*, 1114*m*, 1022*w* 968*w*, 767*w*, 704*m*. **^{1}H-NMR** (500 MHz, $CDCl_3$, 298 K): δ (ppm) =7.41 – 7.38 (m, 2H, H-7), 7.37 – 7.35 (m, 2H, H-6), 7.35 – 7.31 (m, 1H, H-8), 4.35 (q, $^3J_{H,H}$ = 6.8 Hz, 1H, H-9), 2.98 – 2.92 (m, 1H, H-3a), 2.86 – 2.79 (m, 1H, H-2a), 2.56 – 2.48 (m, 1H, H-2b), 2.48 – 2.42 (m, 1H, H-3b), 1.33 (d, $^3J_{H,H}$ = 6.7 Hz, 3H, H-10). **^{13}C{^{1}H}-NMR** (126 MHz, $CDCl_3$, 298 K): δ (ppm) = 176.3 (C-1), 141.6 (C-5), 128.9 (C-7), 128.4 (C-8), 124.9 (C-6), 89.6 (C-4), 65.0 (C-9), 33.7 (C-3), 28.9 (C-2), 19.7 (C-10). **HRMS (ESI)**: *m/z* = 247.0496 calcd. for $C_{12}H_{13}ClNaO_2^+$ [M+Na$^+$]; found: 247.0511.

***rac-anti*-8b**:

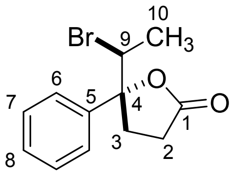

IR (neat): 2980*br*, 1780*s*, 1448*w*, 1217*m*, 1175*m*, 1110*m*, 1016*w*, 963*w*, 765*w*, 703*w*. **^{1}H-NMR** (500 MHz, $CDCl_3$, 298 K): δ (ppm) = 7.41 – 7.37 (m, 2H, H-7), 7.37 – 7.35 (m, 2H, H-6), 7.34 – 7.30 (m, 1H, H-8), 4.46 (q, $^3J_{H,H}$ = 6.9 Hz, 1H), 2.98 – 2.87 (m, 1H, H-3a), 2.87 – 2.79 (m, 1H, H-2a), 2.55 – 2.45 (m, 2H, H-3b; H-2b), 1.50 (d, $^3J_{H,H}$ = 6.9 Hz, 3H, H-10). **^{13}C{^{1}H}-NMR** (126 MHz, $CDCl_3$, 298 K): δ (ppm) = 176.1 (C-1), 141.1 (C-5), 128.9 (C-7), 128.4 (C-8), 125.0 (C-6), 89.4 (C-4), 57.8 (C-9), 34.9 (C-3), 28.9 (C-2), 21.1 (C-10). **HRMS (ESI)**: *m/z* = 290.9991 calcd. for $C_{12}H_{13}BrNaO_2^+$ [M+Na$^+$]; found: 291.0020.

2.4. Crystallographic data

X-Ray diffraction: Data sets for compounds ***anti*-6a**, ***syn*-6a**, ***anti*-6b**, ***anti*-7a** and ***anti*-7b** were collected with a Bruker D8 Venture CMOS diffractometer. For compound ***syn*-6b** data sets were collected with a Bruker APEX II CCD diffractometer. Programs used: data collection: APEX3 V2016.1-0 [16] (Bruker AXS Inc., **2016**); cell refinement: SAINT V8.37A (Bruker AXS Inc., **2015**); data reduction: SAINT V8.37A (Bruker AXS Inc., **2015**); absorption correction, SADABS V2014/7 (Bruker AXS Inc., **2014**); structure solution *SHELXT-2015* [17] (Sheldrick, G. M. *Acta Cryst.,* **2015**, *A71*, 3-8); structure refinement *SHELXL-2015* [18] (Sheldrick, G. M. *Acta Cryst.,* **2015**, *C71* (1), 3-8) and graphics, *XP* [19] (Version 5.1, Bruker AXS Inc., Madison, Wisconsin, USA, **1998**). *R*-values are given for observed reflections, and wR^2 values are given for all reflections.

X-ray crystal structure analysis of *anti*-6a: A colourless prism-like specimen of $C_{12}H_{11}ClO_2$, approximate dimensions 0.043 mm x 0.081 mm x 0.109 mm, was used for the X-ray crystallographic analysis. The X-ray intensity data were measured. A total of 1032 frames were collected. The total exposure time was 8.60 hours. The frames were integrated with the Bruker SAINT software package using a narrow-frame algorithm. The integration of the data using a triclinic unit cell yielded a total of 9720 reflections to a maximum θ angle of 26.73° (0.79 Å resolution), of which 2161 were independent (average redundancy 4.498, completeness = 98.0%, R_{int} = 3.50%, R_{sig} = 2.71%) and 1950 (90.24%) were greater than 2σ(F^2). The final cell constants of a = 8.1989(4) Å, b = 8.4173(4) Å, c = 8.8635(4) Å, α = 68.933(2)°, β = 76.834(2)°, γ = 65.982(2)°, volume = 519.00(4) $Å^3$, are based upon the refinement of the XYZ-centroids of 4281 reflections above 20 σ(I) with 5.464° < 2θ < 55.71°. Data were corrected for absorption effects using the multi-scan method (SADABS). The ratio of minimum to maximum apparent transmission was 0.932. The calculated minimum and maximum transmission coefficients (based on crystal size) are 0.9640 and 0.9850. The structure was solved and refined using the Bruker SHELXTL Software Package, using the space group *P*-1, with Z = 2 for the formula unit, $C_{12}H_{11}ClO_2$. The final anisotropic full-matrix least-squares refinement on F^2 with 136 variables converged at R1 = 3.90%, for the observed data and wR2 = 8.38% for all data. The goodness-of-fit was 1.140. The largest peak in the final difference electron density synthesis was 0.348 $e^-/Å^3$ and the largest hole was -0.209 $e^-/Å^3$ with an RMS deviation of 0.052 $e^-/Å^3$. On the basis of the final model, the calculated density was 1.425 g/cm^3 and F(000), 232 e^-. CCDC Nr.: 2056209.

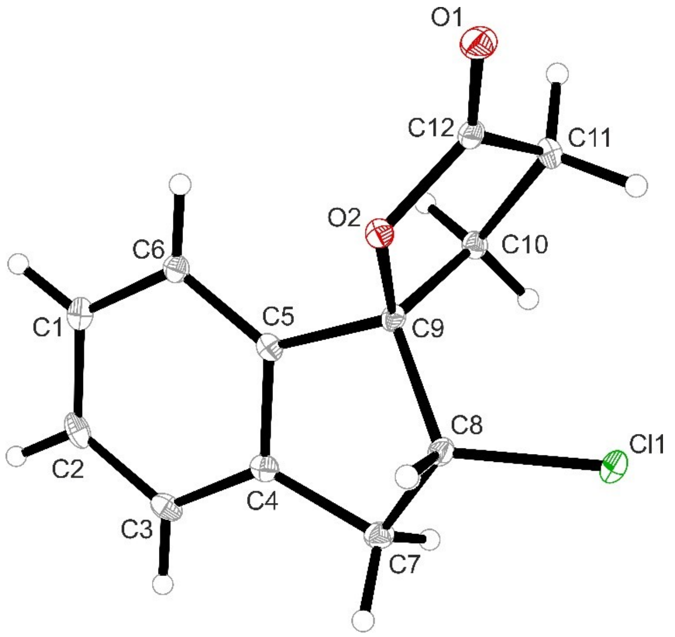

*Figure S3: Crystal structure of compound **anti-6a**. Thermal ellipsoids are shown at 30% probability.*

X-ray crystal structure analysis of *syn*-6a: A colourless prism-like specimen of $C_{12}H_{11}ClO_2$, approximate dimensions 0.098 mm x 0.188 mm x 0.194 mm, was used for the X-ray crystallographic analysis. The X-ray intensity data were measured. A total of 1149 frames were collected. The total exposure time was 7.98 hours. The frames were integrated with the Bruker SAINT software package using a narrow-frame algorithm. The integration of the data using a monoclinic unit cell yielded a total of 23308 reflections to a maximum θ angle of 27.49° (0.77 Å resolution), of which 2335 were independent (average redundancy 9.982, completeness = 98.8%, R_{int} = 2.44%, R_{sig} = 1.10%) and 2257 (96.66%) were greater than 2σ(F^2). The final cell constants of a = 5.75830(10) Å, b = 9.7431(2) Å, c = 18.3135(5) Å, β = 94.0580(10)°, volume = 1024.88(4) Å^3, are based upon the refinement of the XYZ-centroids of 9930 reflections above 20 σ(I) with 8.237° < 2θ < 54.94°. Data were corrected for absorption effects using the multi-scan method (SADABS). The ratio of minimum to maximum apparent transmission was 0.952. The calculated minimum and maximum transmission coefficients (based on crystal size) are 0.9360 and 0.9670. The structure was solved and refined using the Bruker SHELXTL Software Package, using the space group $P2_1/n$, with Z = 4 for the formula unit, $C_{12}H_{11}ClO_2$. The final anisotropic full-matrix least-squares refinement on F^2 with 136 variables converged at R1 = 2.76%, for the observed data and wR2 = 6.80% for all data. The goodness-of-fit was 1.043. The largest peak in the final difference electron density synthesis was 0.383 e$^-$/Å^3 and the largest hole was -0.188 e$^-$/Å^3 with an RMS deviation of 0.042 e$^-$/Å^3. On the basis of the final model, the calculated density was 1.443 g/cm^3 and F(000), 464 e$^-$. CCDC Nr.: 2056208.

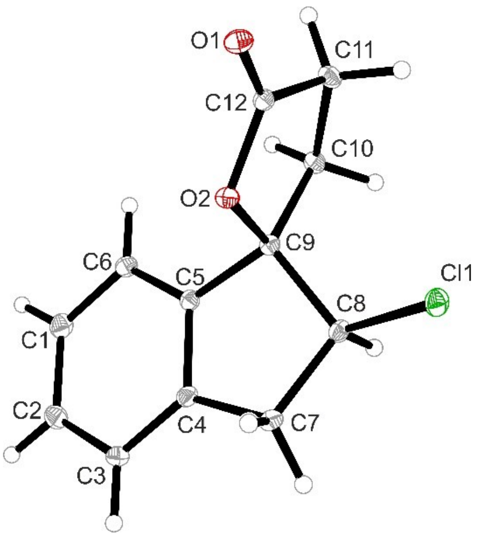

*Figure S2: Crystal structure of compound **syn-6a**. Thermal ellipsoids are set at 30% probability.*

X-ray crystal structure analysis of *anti*-6b: A colourless prism-like specimen of $C_{12}H_{11}BrO_2$, approximate dimensions 0.165 mm x 0.180 mm x 0.278 mm, was used for the X-ray crystallographic analysis. The X-ray intensity data were measured. A total of 588 frames were collected. The total exposure time was 3.27 hours. The frames were integrated with the Bruker SAINT software package using a narrow-frame algorithm. The integration of the data using a triclinic unit cell yielded a total of 9320 reflections to a maximum θ angle of 27.51° (0.77 Å resolution), of which 2413 were independent (average redundancy 3.862, completeness = 98.8%, R_{int} = 1.58%, R_{sig} = 1.40%) and 2333 (96.68%) were greater than $2\sigma(F^2)$. The final cell constants of a = 8.3537(2) Å, b = 8.5198(2) Å, c = 8.8355(3) Å, α = 69.1250(10)°, β = 76.7640(10)°, γ = 65.2120(10)°, volume = 531.08(3) $Å^3$, are based upon the refinement of the XYZ-centroids of 7218 reflections above 20 σ(I) with 4.956° < 2θ < 55.02°. Data were corrected for absorption effects using the multi-scan method (SADABS). The ratio of minimum to maximum apparent transmission was 0.922. The calculated minimum and maximum transmission coefficients (based on crystal size) are 0.4150 and 0.5700. The structure was solved and refined using the Bruker SHELXTL Software Package, using the space group *P*-1, with Z = 2 for the formula unit, $C_{12}H_{11}BrO_2$. The final anisotropic full-matrix least-squares refinement on F^2 with 136 variables converged at R1 = 1.56%, for the observed data and wR2 = 3.76% for all data. The goodness-of-fit was 1.050. The largest peak in the final difference electron density synthesis was 0.360 $e^-/Å^3$ and the largest hole was -0.223 $e^-/Å^3$ with an RMS deviation of 0.046 $e^-/Å^3$. On the basis of the final model, the calculated density was 1.670 g/cm^3 and F(000), 268 e^-. CCDC Nr.: 2056211.

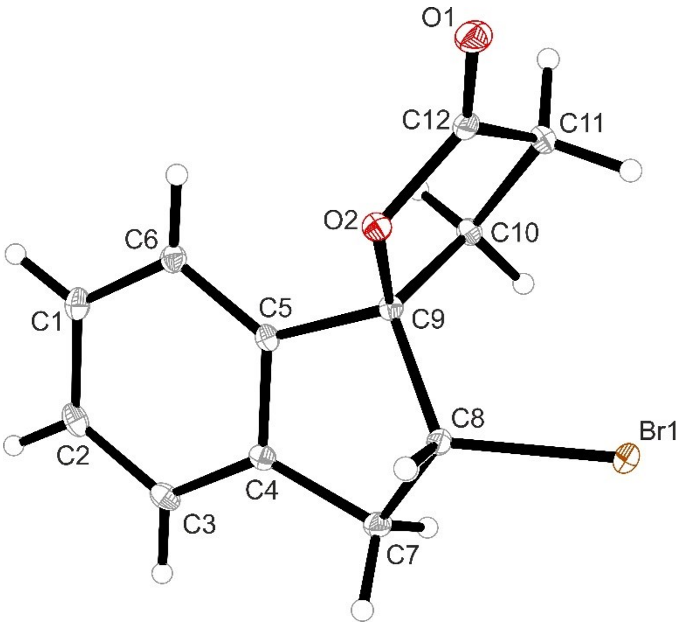

*Figure S5: Crystal structure of compound **anti-6b**. Thermal ellipsoids are shown at 30% probability.*

X-ray crystal structure analysis of *syn*-6b: A colourless prism-like specimen of $C_{12}H_{11}BrO_2$, approximate dimensions 0.120 mm x 0.200 mm x 0.280 mm, was used for the X-ray crystallographic analysis. The X-ray intensity data were measured. A total of 958 frames were collected. The total exposure time was 8.19 hours. The frames were integrated with the Bruker SAINT software package using a wide-frame algorithm. The integration of the data using a monoclinic unit cell yielded a total of 8488 reflections to a maximum θ angle of 66.66° (0.84 Å resolution), of which 1813 were independent (average redundancy 4.682, completeness = 99.3%, R_{int} = 3.23%, R_{sig} = 2.53%) and 1746 (96.30%) were greater than 2σ(F^2). The final cell constants of a = 5.83760(10) Å, b = 9.7639(2) Å, c = 18.2223(4) Å, β = 94.1490(10)°, volume = 1035.91(4) $Å^3$, are based upon the refinement of the XYZ-centroids of 5606 reflections above 20 σ(I) with 9.733° < 2θ < 133.2°. Data were corrected for absorption effects using the multi-scan method (SADABS). The ratio of minimum to maximum apparent transmission was 0.732. The calculated minimum and maximum transmission coefficients (based on crystal size) are 0.3230 and 0.5740. The structure was solved and refined using the Bruker SHELXTL Software Package, using the space group $P2_1/n$, with Z = 4 for the formula unit, $C_{12}H_{11}BrO_2$. The final anisotropic full-matrix least-squares refinement on F^2 with 136 variables converged at R1 = 2.42%, for the observed data and wR2 = 5.89% for all data. The goodness-of-fit was 1.126. The largest peak in the final difference electron density synthesis was 0.365 $e^-/Å^3$ and the largest hole was -0.421 $e^-/Å^3$ with an RMS deviation of 0.076 $e^-/Å^3$. On the basis of the final model, the calculated density was 1.713 g/cm^3 and F(000), 536 e^-. CCDC Nr.: 2056210.

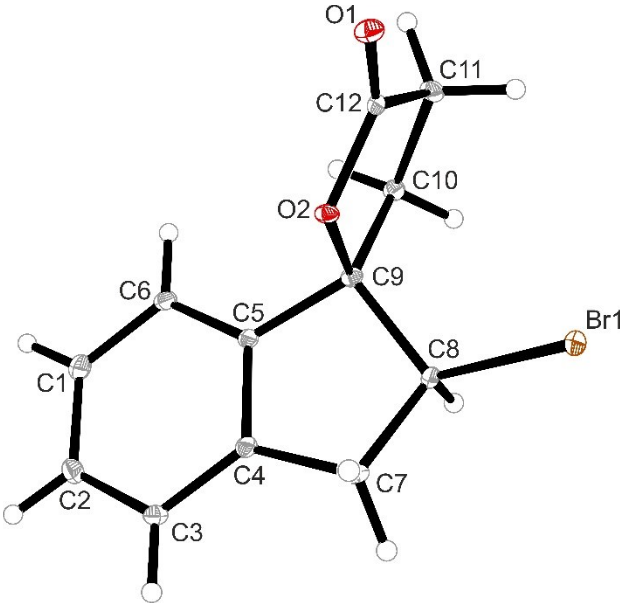

*Figure S4: Crystal structure of compound **syn-6b**. Thermal ellipsoids are shown at 30% probability.*

X-ray crystal structure analysis of *anti*-7a: A colourless plate-like specimen of $C_{13}H_{13}ClO_2$, approximate dimensions 0.057 mm x 0.121 mm x 0.143 mm, was used for the X-ray crystallographic analysis. The X-ray intensity data were measured on a Bruker D8 Venture PHOTON III Diffractometer system equipped with a micro focus tube Cu Ims (CuK_{α}, λ = 1.54178 Å) and a MX mirror monochromator. A total of 1158 frames were collected. The total exposure time was 13.88 hours. The frames were integrated with the Bruker SAINT software package using a wide-frame algorithm. The integration of the data using a triclinic unit cell yielded a total of 7594 reflections to a maximum θ angle of 66.92° (0.84 Å resolution), of which 1910 were independent (average redundancy 3.976, completeness = 98.5%, R_{int} = 2.76%, R_{sig} = 2.70%) and 1828 (95.71%) were greater than 2σ(F^2). The final cell constants of a = 8.5302(3) Å, b = 8.5648(3) Å, c = 8.8272(3) Å, α = 76.7530(10)°, β = 69.1750(10)°, γ = 65.8030(10)°, volume = 547.18(3) $Å^3$, are based upon the refinement of the XYZ-centroids of 5764 reflections above 20 σ(I) with 11.90° < 2θ < 133.8°. Data were corrected for absorption effects using the Multi-Scan method (SADABS). The ratio of minimum to maximum apparent transmission was 0.885. The calculated minimum and maximum transmission coefficients (based on crystal size) are 0.6790 and 0.8510. The structure was solved and refined using the Bruker SHELXTL Software Package, using the space group *P*-1, with Z = 2 for the formula unit, $C_{13}H_{13}ClO_2$. The final anisotropic full-matrix least-squares refinement on F^2 with 145 variables converged at R1 = 2.71%, for the observed data and wR2 = 7.11% for all data. The goodness-of-fit was 1.070. The largest peak in the final difference electron density synthesis was 0.294 $e^-/Å^3$ and the largest hole was -0.224 $e^-/Å^3$ with an RMS deviation of 0.042 $e^-/Å^3$. On the basis of the final model, the calculated density was 1.437 g/cm^3 and F(000), 248 e^-. CCDC Nr.: 2056212.

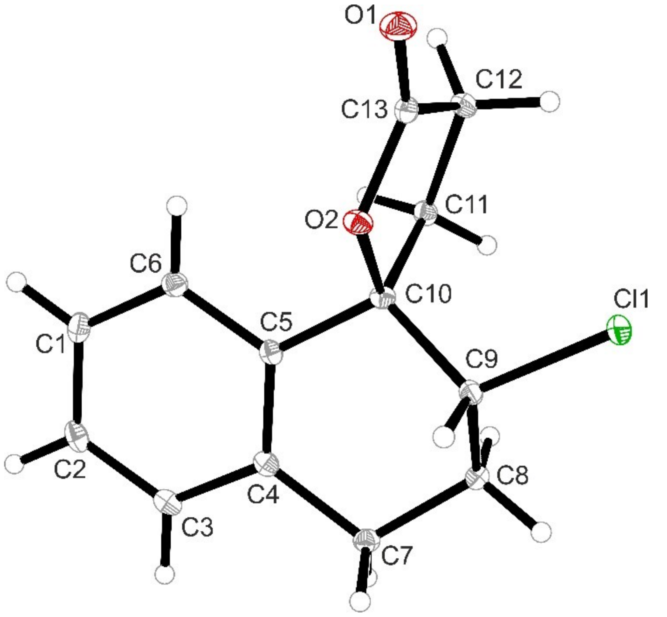

*Figure S6: Crystal structure of compound **anti-7a**. Thermal ellipsoids are shown at 30% probability.*

X-ray crystal structure analysis of *anti*-7b: A colourless prism-like specimen of $C_{13}H_{13}BrO_2$, approximate dimensions 0.057 mm x 0.090 mm x 0.168 mm, was used for the X-ray crystallographic analysis. The X-ray intensity data were measured on a Bruker D8 Venture PHOTON III Diffractometer system equipped with a micro focus tube Mo Ims (MoK_{α}, λ = 0.71073 Å) and a MX mirror monochromator. A total of 408 frames were collected. The total exposure time was 1.13 hours. The frames were integrated with the Bruker SAINT software package using a narrow-frame algorithm. The integration of the data using a triclinic unit cell yielded a total of 9047 reflections to a maximum θ angle of 26.77° (0.79 Å resolution), of which 2386 were independent (average redundancy 3.792, completeness = 99.6%, R_{int} = 3.71%, R_{sig} = 3.12%) and 2207 (92.50%) were greater than $2\sigma(F^2)$. The final cell constants of a = 8.5352(2) Å, b = 8.6441(2) Å, c = 8.8394(2) Å, α = 77.5970(10)°, β = 69.8180(10)°, γ = 67.0810(10)°, volume = 561.35(2) Å^3, are based upon the refinement of the XYZ-centroids of 4344 reflections above 20 σ(I) with 4.931° < 2θ < 53.51°. Data were corrected for absorption effects using the Multi-Scan method (SADABS). The ratio of minimum to maximum apparent transmission was 0.931. The calculated minimum and maximum transmission coefficients (based on crystal size) are 0.5800 and 0.8190. The structure was solved and refined using the Bruker SHELXTL Software Package, using the space group *P*-1, with Z = 2 for the formula unit, $C_{13}H_{13}BrO_2$. The final anisotropic full-matrix least-squares refinement on F^2 with 145 variables converged at R1 = 2.22%, for the observed data and wR2 = 5.03% for all data. The goodness-of-fit was 1.046. The largest peak in the final difference electron density synthesis was 0.466 $e^-/Å^3$ and the largest hole was -0.228 $e^-/Å^3$ with an RMS deviation of 0.065 $e^-/Å^3$. On the basis of the final model, the calculated density was 1.663 g/cm^3 and F(000), 284 e^-. CCDC Nr.: 2056213.

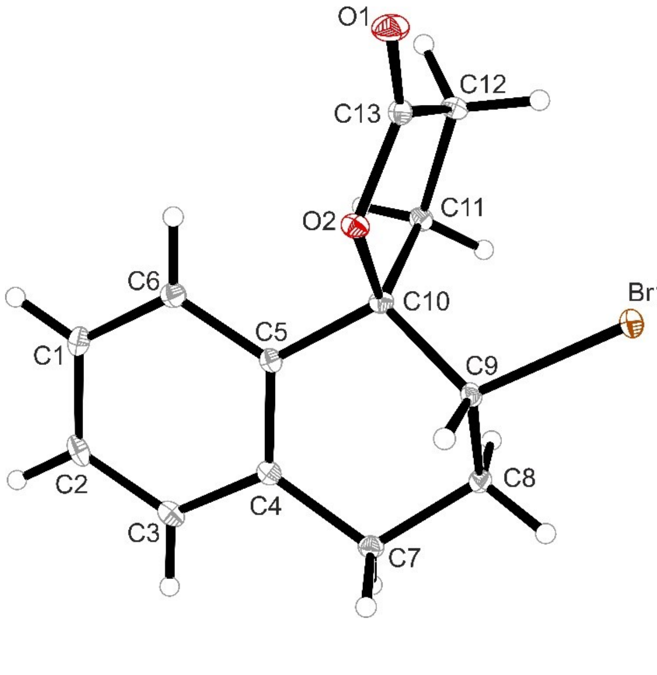

*Figure S7: Crystal structure of compound **anti-7b**. Thermal ellipsoids are shown at 30% probability.*

2.5. ^{1}H- and $^{13}C\{^{1}H\}$-NMR-spectra

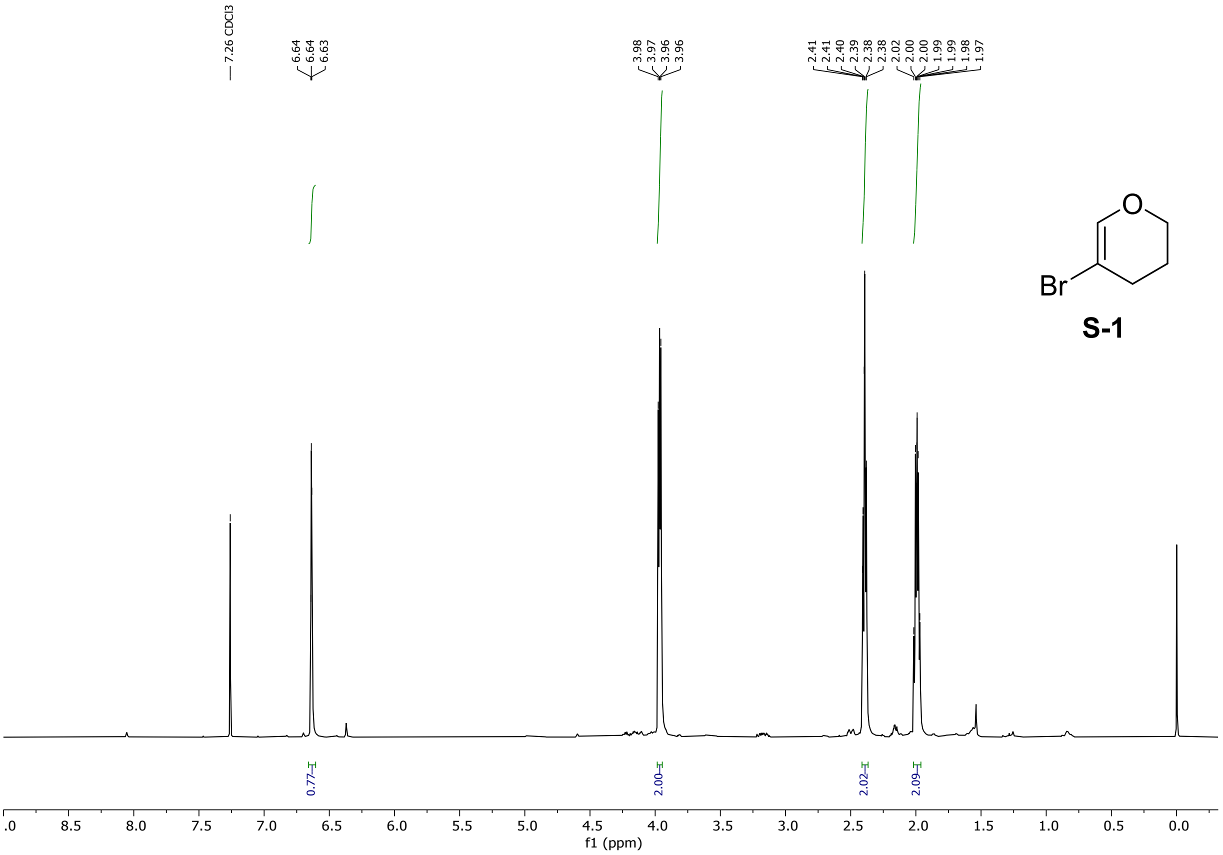

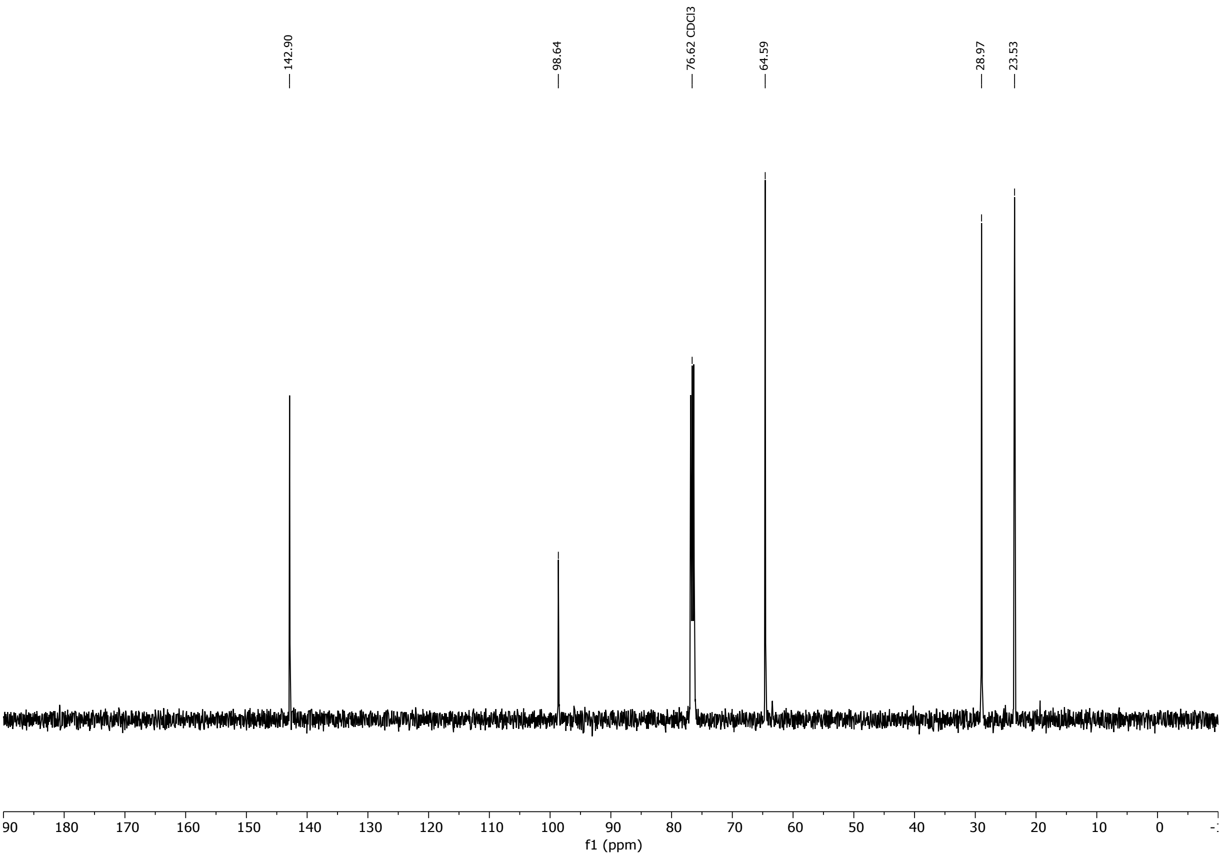

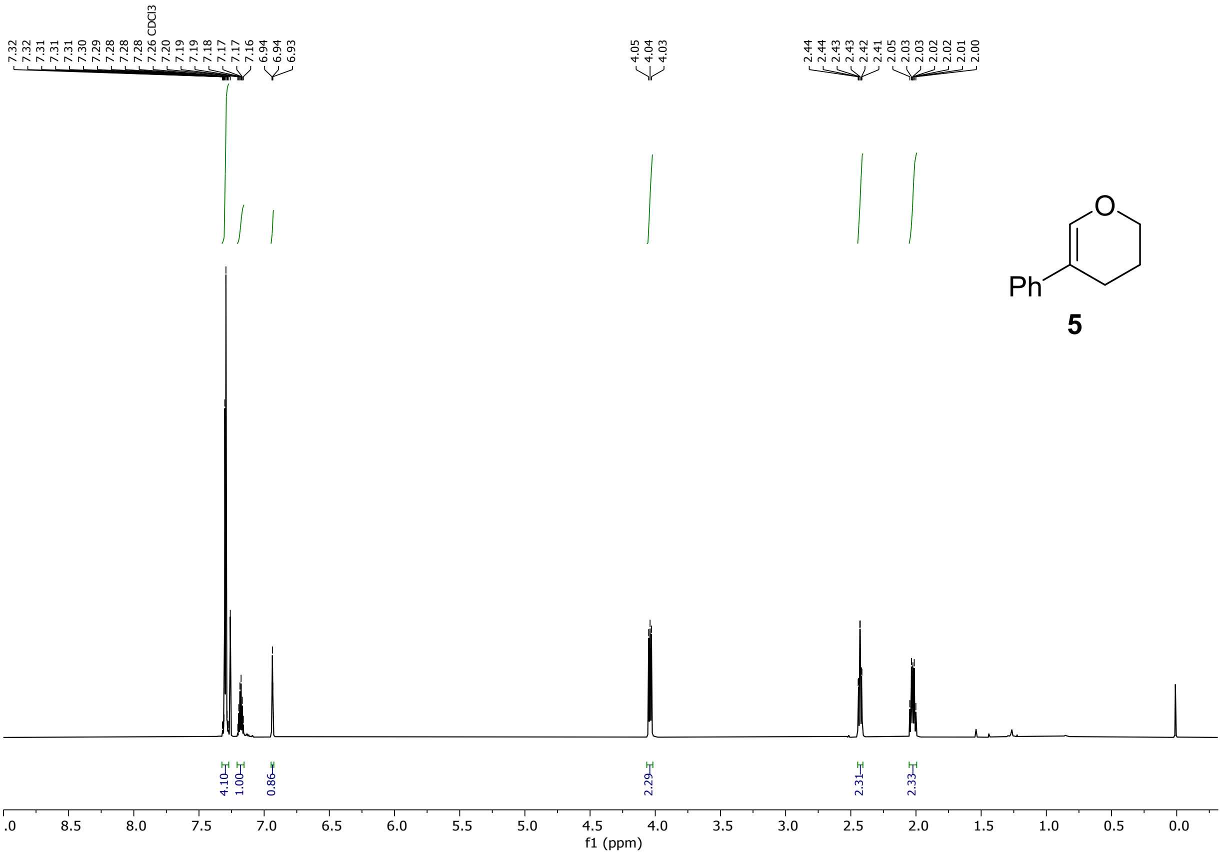

7.32 7.32 7.31 7.31 7.31 7.30 7.29 7.28 7.28 7.28 7.26 CDCl3 7.20 7.19 7.19 7.18 7.17 7.17 7.16 6.94 6.94 6.93
4.05 4.04 4.03
2.44 2.44 2.43 2.43 2.42 2.41 2.05 2.03 2.03 2.02 2.02 2.01 2.00
Ph
5
4.10 1.00 0.86 2.29 2.31 2.33
f1 (ppm)

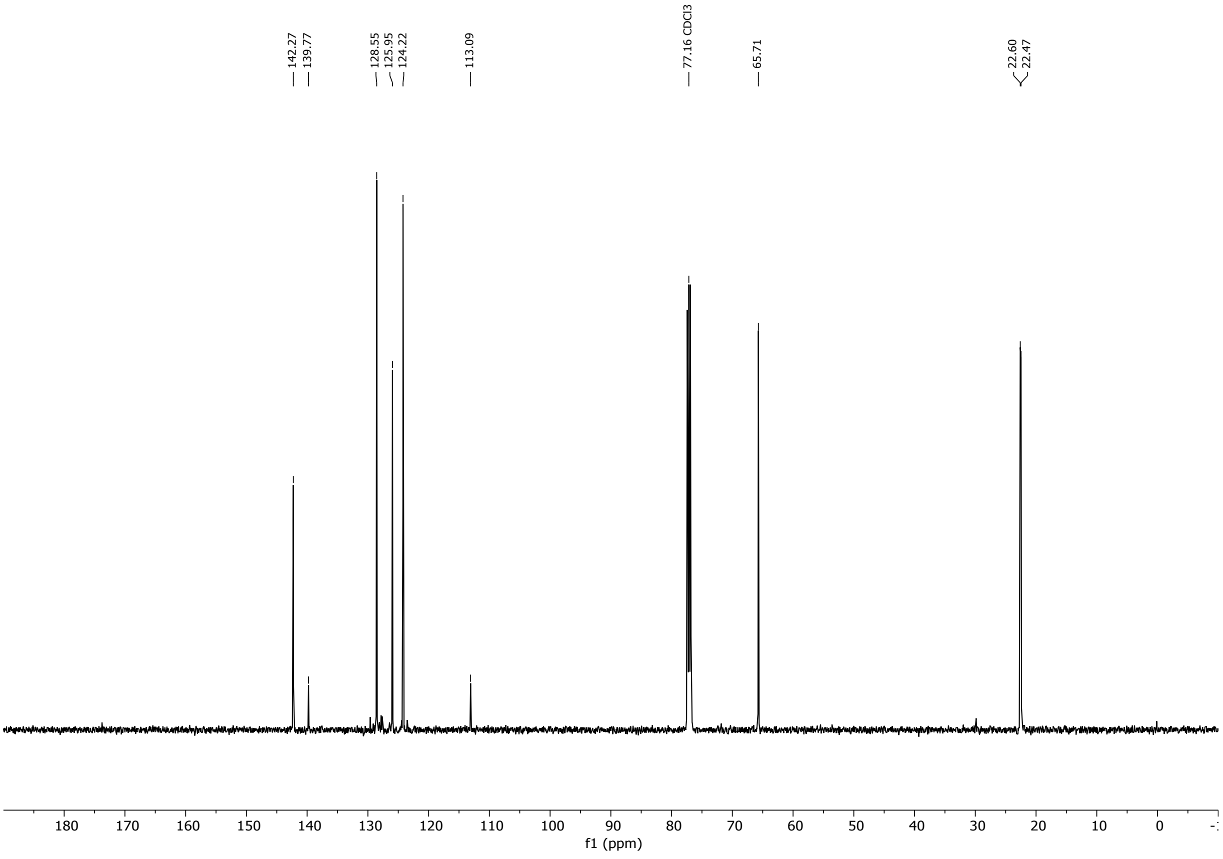

142.27 139.77 128.55 125.95 124.22 113.09 77.16 CDCl3 65.71 22.60 22.47
f1 (ppm)

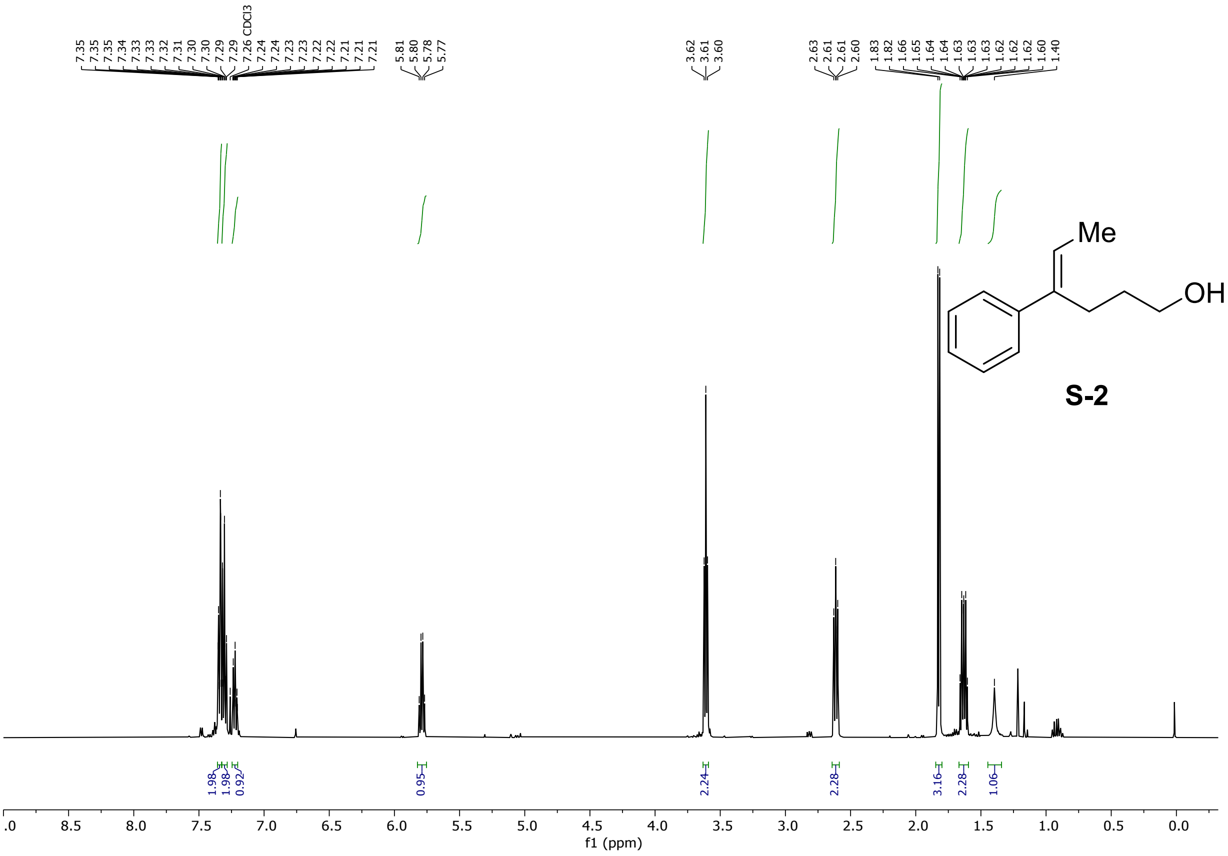
7.35 7.35 7.34 7.33 7.33 7.32 7.31 7.30 7.30 7.29 7.29 7.26 CDCl3 7.24 7.24 7.24 7.23 7.23 7.22 7.22 7.21 7.21 7.21
5.81 5.80 5.78 5.77
3.62 3.61 3.60
2.63 2.61 2.61 2.60
1.83 1.82 1.66 1.65 1.64 1.64 1.63 1.63 1.63 1.62 1.62 1.62 1.60 1.40
Me
OH
S-2
1.98 1.98 0.92 0.95 2.24 2.28 3.16 2.28 1.06
f1 (ppm)

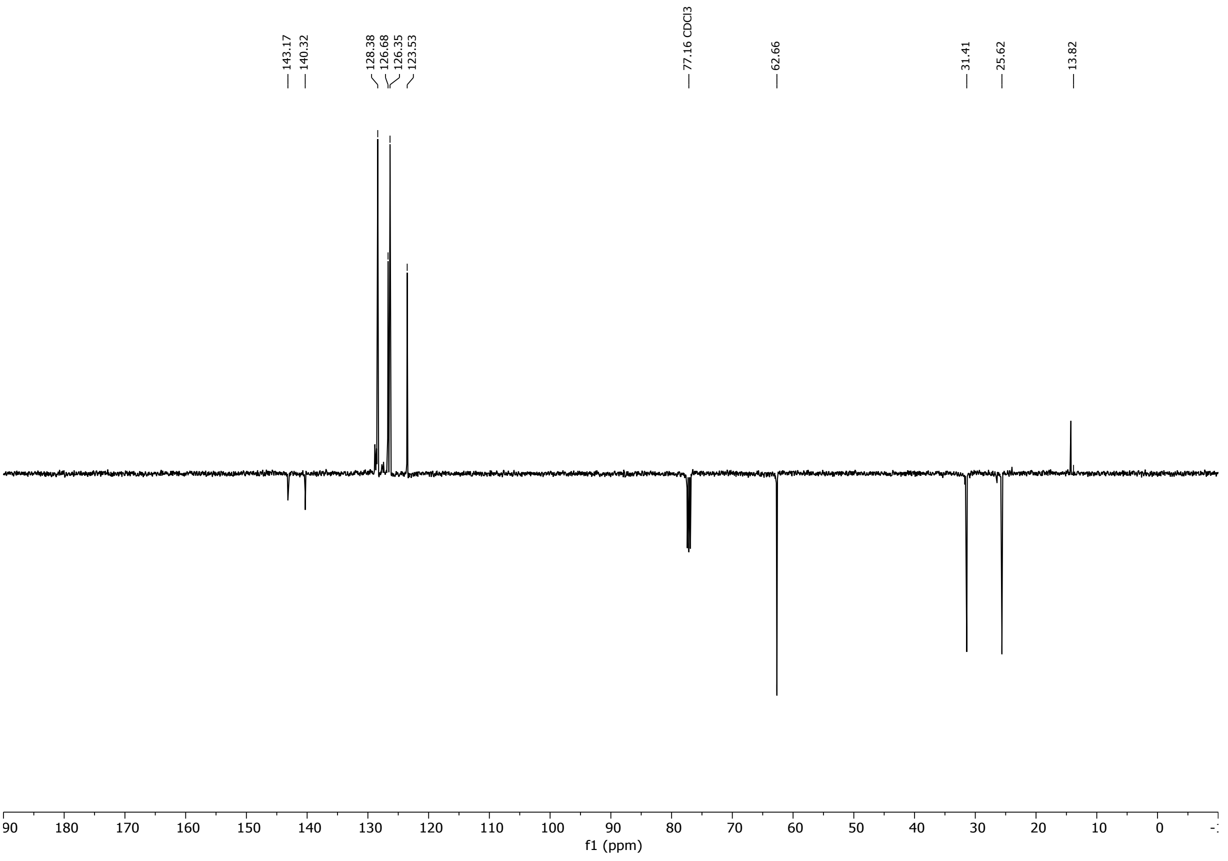
143.17 140.32
128.38 126.68 126.35 123.53
77.16 CDCl3
62.66
31.41 25.62
13.82
f1 (ppm)

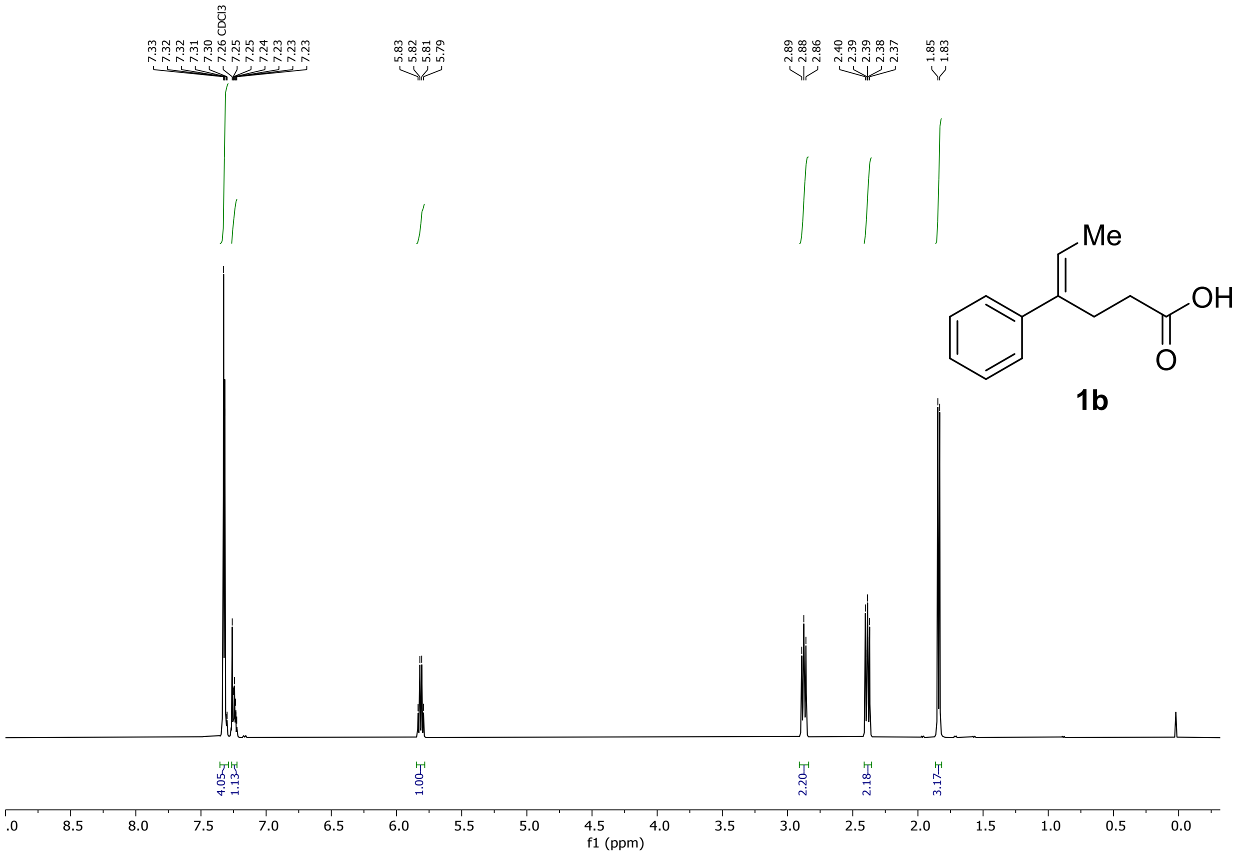
7.33 7.32 7.32 7.31 7.30 7.26 CDCl3 7.25 7.25 7.24 7.23 7.23 7.23
5.83 5.82 5.81 5.79
2.89 2.88 2.86
2.40 2.39 2.39 2.38 2.37
1.85 1.83
Me
OH
O
1b
4.05
1.13
1.00
2.20
2.18
3.17
f1 (ppm)

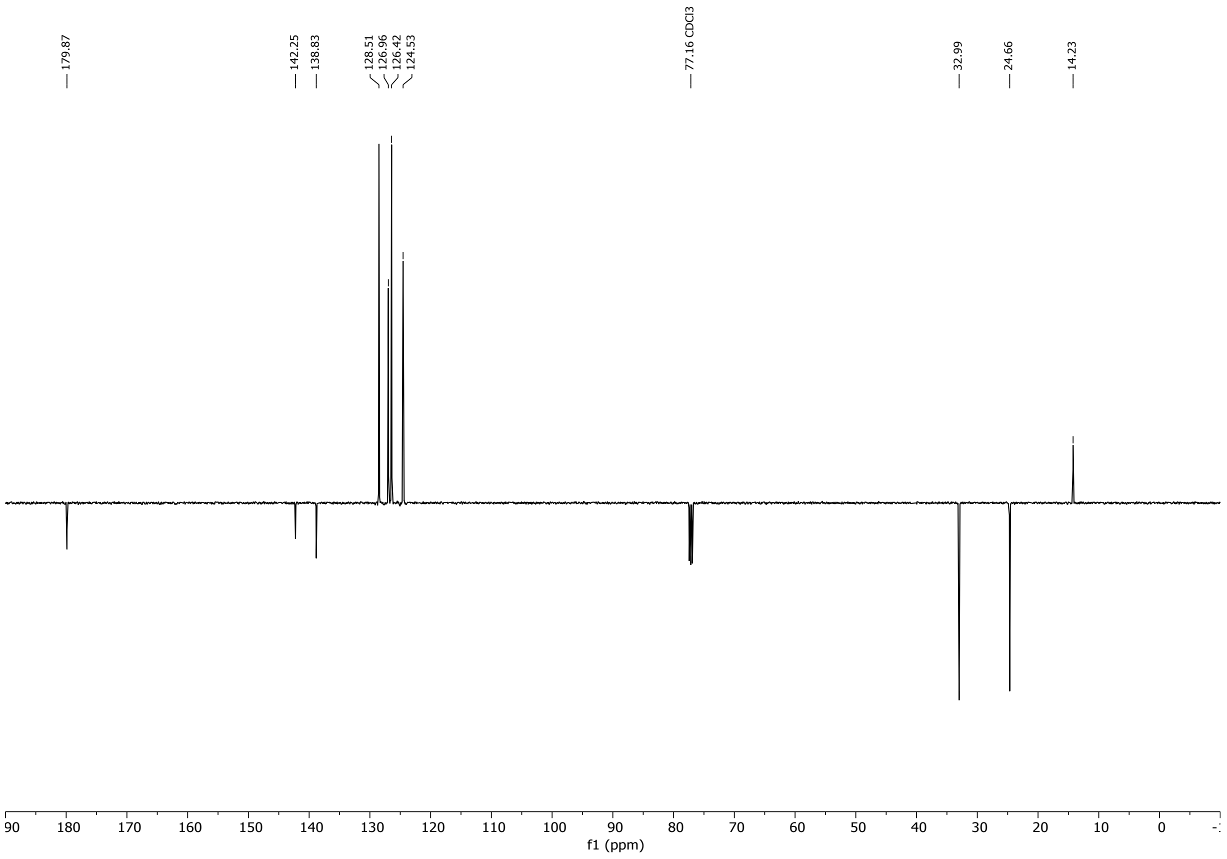
179.87
142.25
138.83
128.51 126.96 126.42 124.53
77.16 CDCl3
32.99
24.66
14.23
f1 (ppm)

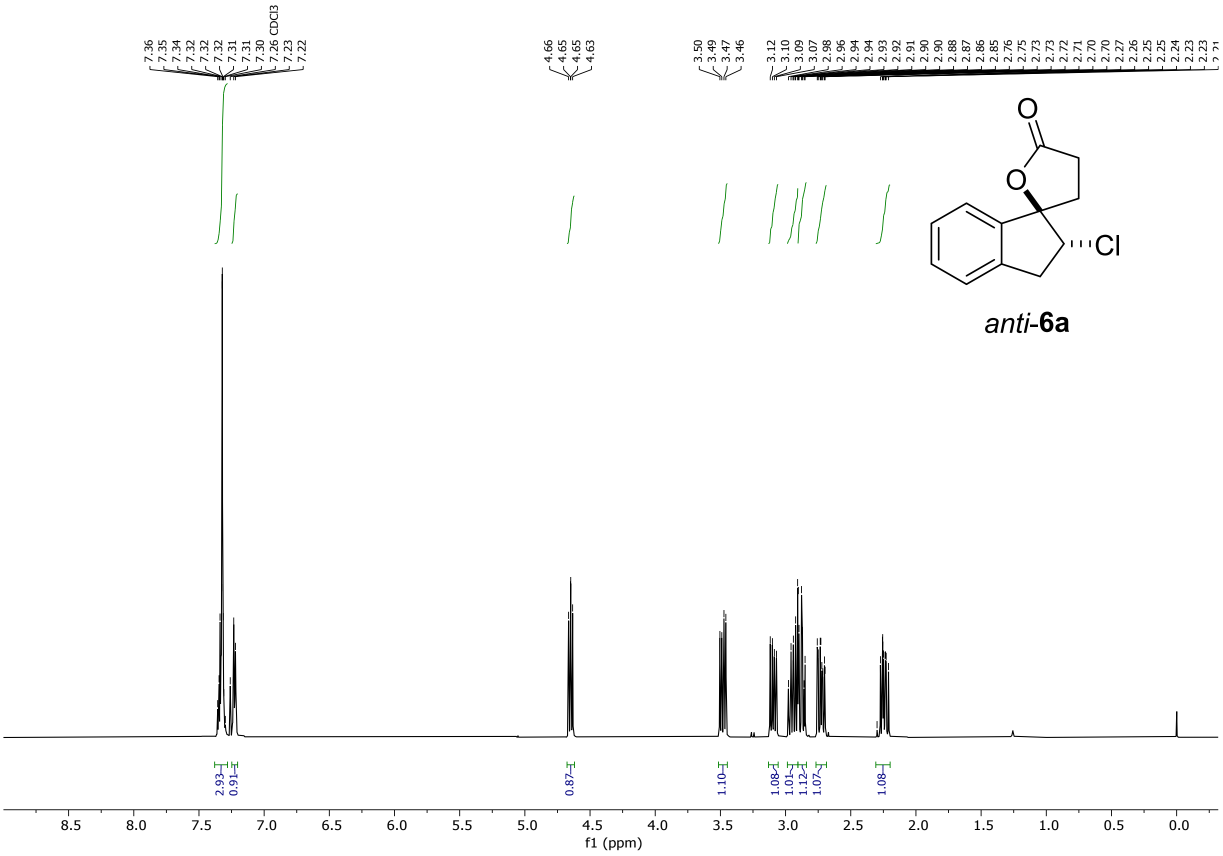
7.36
7.35
7.34
7.32
7.32
7.32
7.31
7.31
7.30
7.26 CDCl3
7.23
7.22
4.66
4.65
4.65
4.63
3.50
3.49
3.47
3.46
3.12
3.10
3.09
3.07
2.98
2.96
2.94
2.94
2.93
2.92
2.91
2.90
2.90
2.88
2.87
2.86
2.85
2.76
2.75
2.73
2.73
2.72
2.71
2.70
2.70
2.27
2.26
2.25
2.25
2.24
2.23
2.23
O
O
Cl
anti-**6a**
2.93
0.91
0.87
1.10
1.08
1.01
1.12
1.07
1.08
8.5
8.0
7.5
7.0
6.5
6.0
5.5
5.0
4.5
4.0
3.5
3.0
2.5
2.0
1.5
1.0
0.5
0.0
f1 (ppm)

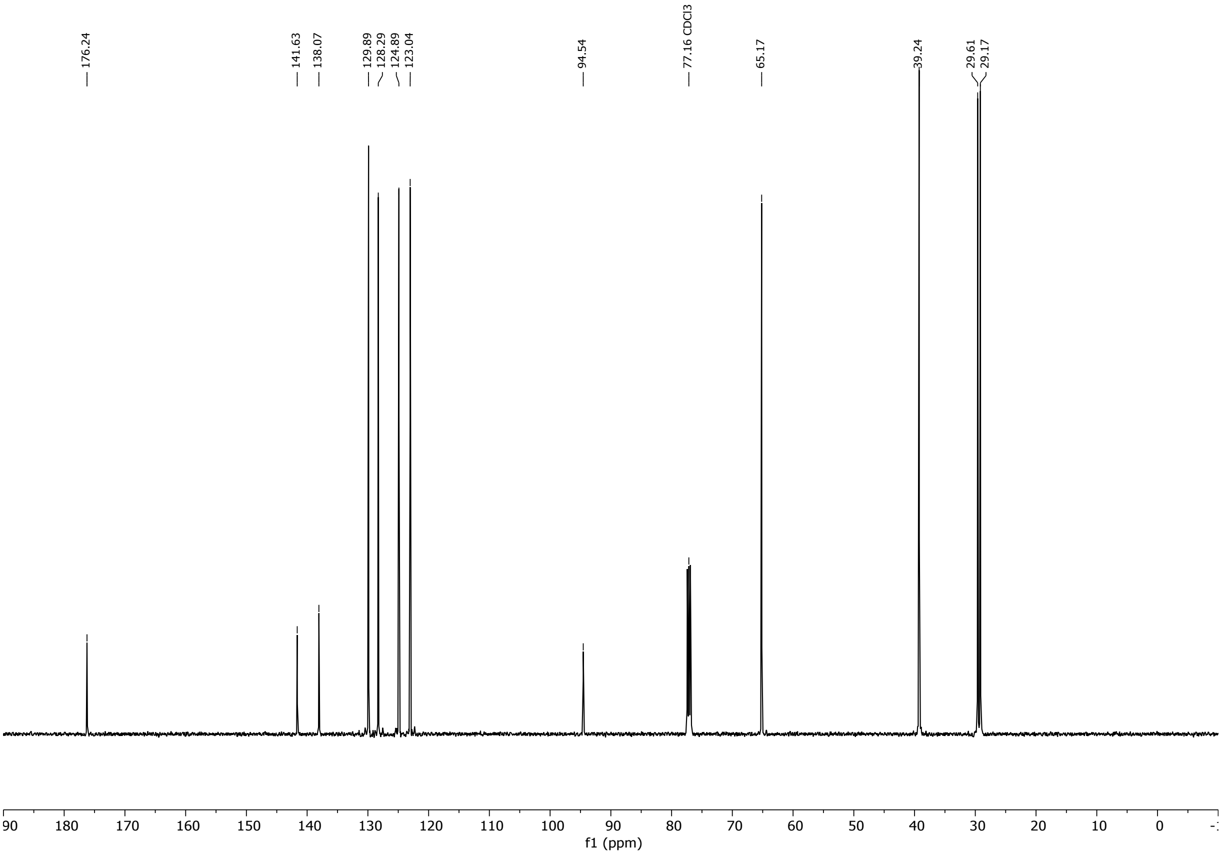
176.24
141.63
138.07
129.89
128.29
124.89
123.04
94.54
77.16 CDCl3
65.17
39.24
29.61
29.17
f1 (ppm)

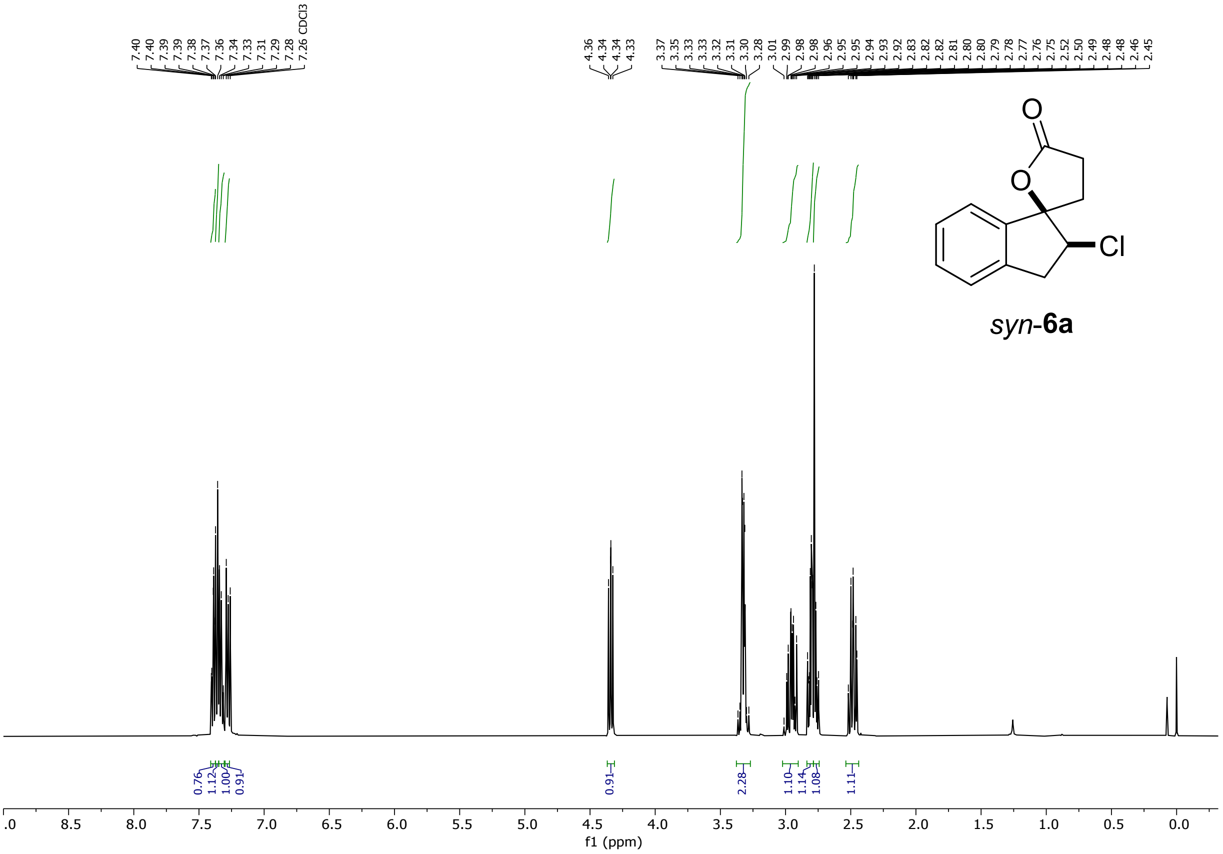
7.40
7.40
7.39
7.39
7.38
7.37
7.36
7.34
7.33
7.31
7.29
7.28
7.26 CDCl3
4.36
4.34
4.34
4.33
3.37
3.35
3.33
3.33
3.32
3.31
3.30
3.28
3.01
2.99
2.98
2.98
2.96
2.95
2.95
2.94
2.93
2.92
2.83
2.82
2.82
2.81
2.80
2.80
2.79
2.78
2.77
2.76
2.75
2.52
2.50
2.49
2.48
2.48
2.46
2.45
0.76
1.12
1.00
0.91
0.91
2.28
1.10
1.14
1.08
1.11
O
Cl
f1 (ppm)

syn-**6a**

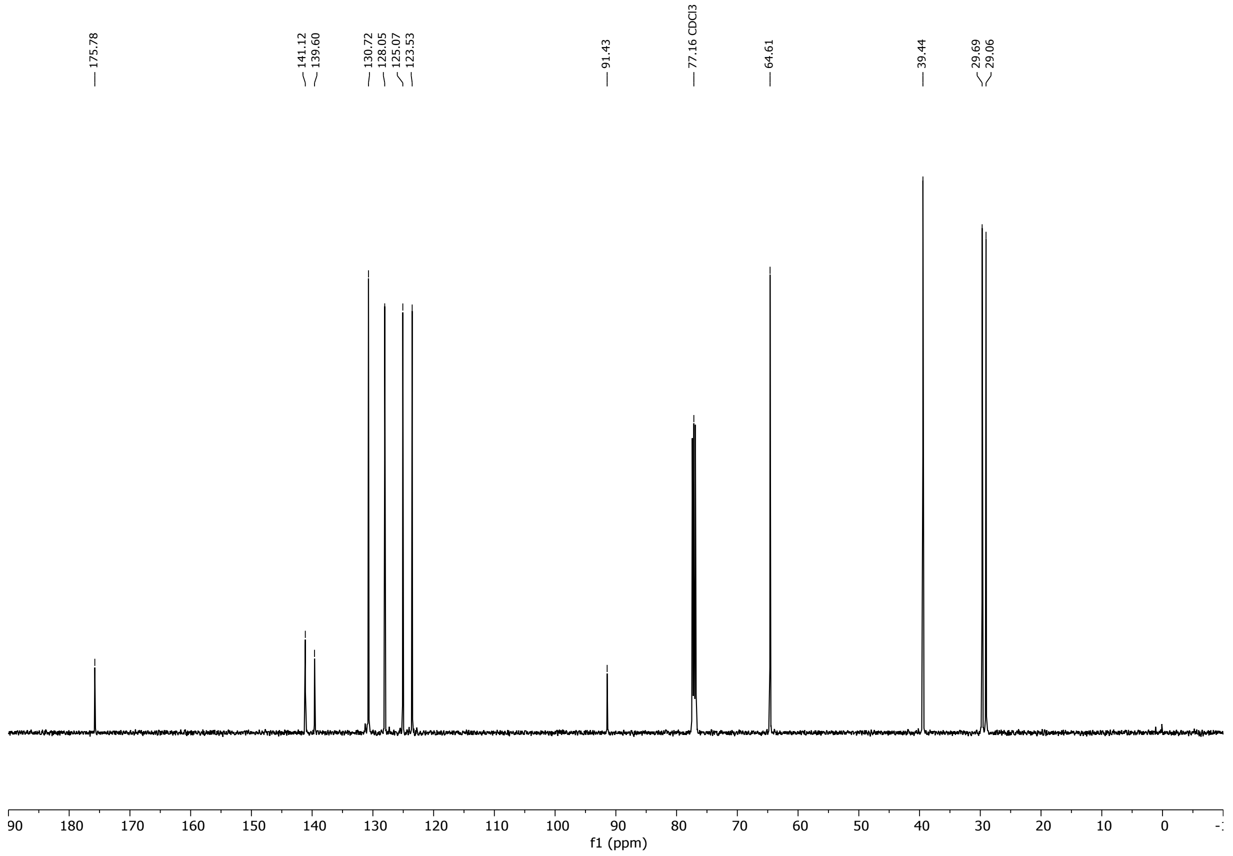
175.78
141.12
139.60
130.72
128.05
125.07
123.53
91.43
77.16 CDCl3
64.61
39.44
29.69
29.06
f1 (ppm)

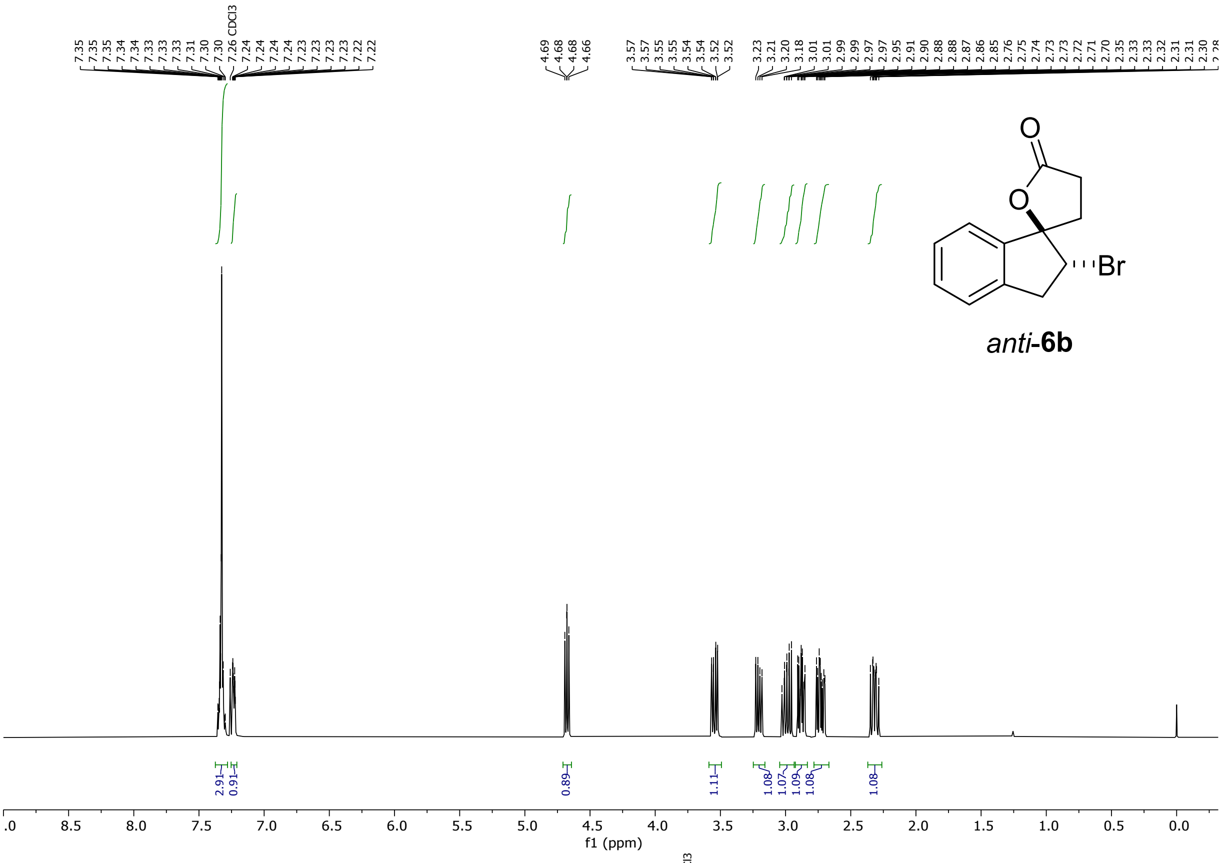
7.35 7.35 7.35 7.34 7.34 7.33 7.33 7.33 7.31 7.30 7.30 7.26 CDCl3 7.24 7.24 7.24 7.24 7.24 7.23 7.23 7.23 7.23 7.22 7.22
4.69 4.68 4.68 4.66
3.57 3.57 3.55 3.55 3.54 3.54 3.52 3.52
3.23 3.21 3.20 3.18 3.01 3.01 2.99 2.99 2.97 2.97 2.95 2.91 2.90 2.88 2.88 2.87 2.86 2.85 2.76 2.75 2.74 2.73 2.73 2.72 2.71 2.70 2.35 2.33 2.33 2.32 2.31 2.31 2.30
O
O
Br
anti-6b
2.91
0.91
0.89
1.11
1.08
1.07
1.09
1.08
1.08
8.5 8.0 7.5 7.0 6.5 6.0 5.5 5.0 4.5 4.0 3.5 3.0 2.5 2.0 1.5 1.0 0.5 0.0
f1 (ppm)

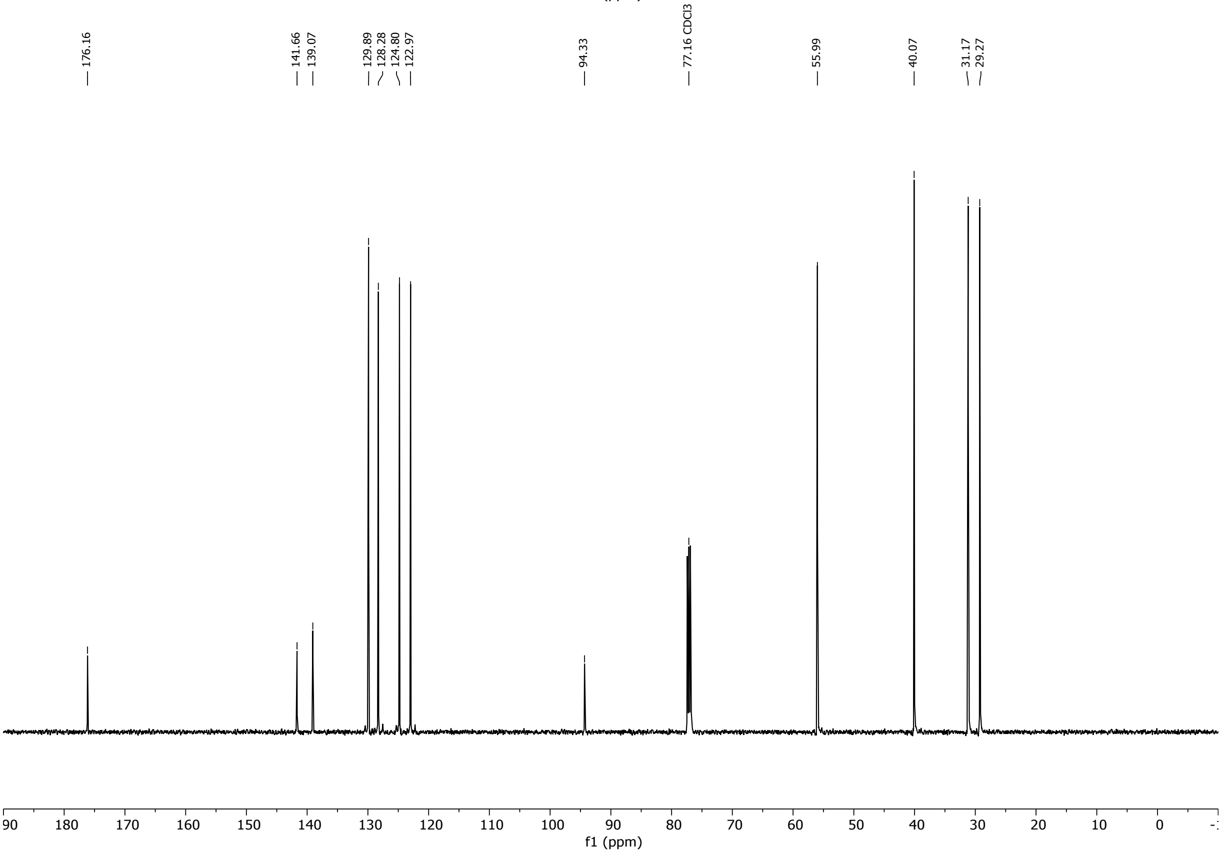
176.16
141.66 139.07
129.89 128.28 124.80 122.97
94.33
77.16 CDCl3
55.99
40.07
31.17 29.27
180 170 160 150 140 130 120 110 100 90 80 70 60 50 40 30 20 10 0
f1 (ppm)

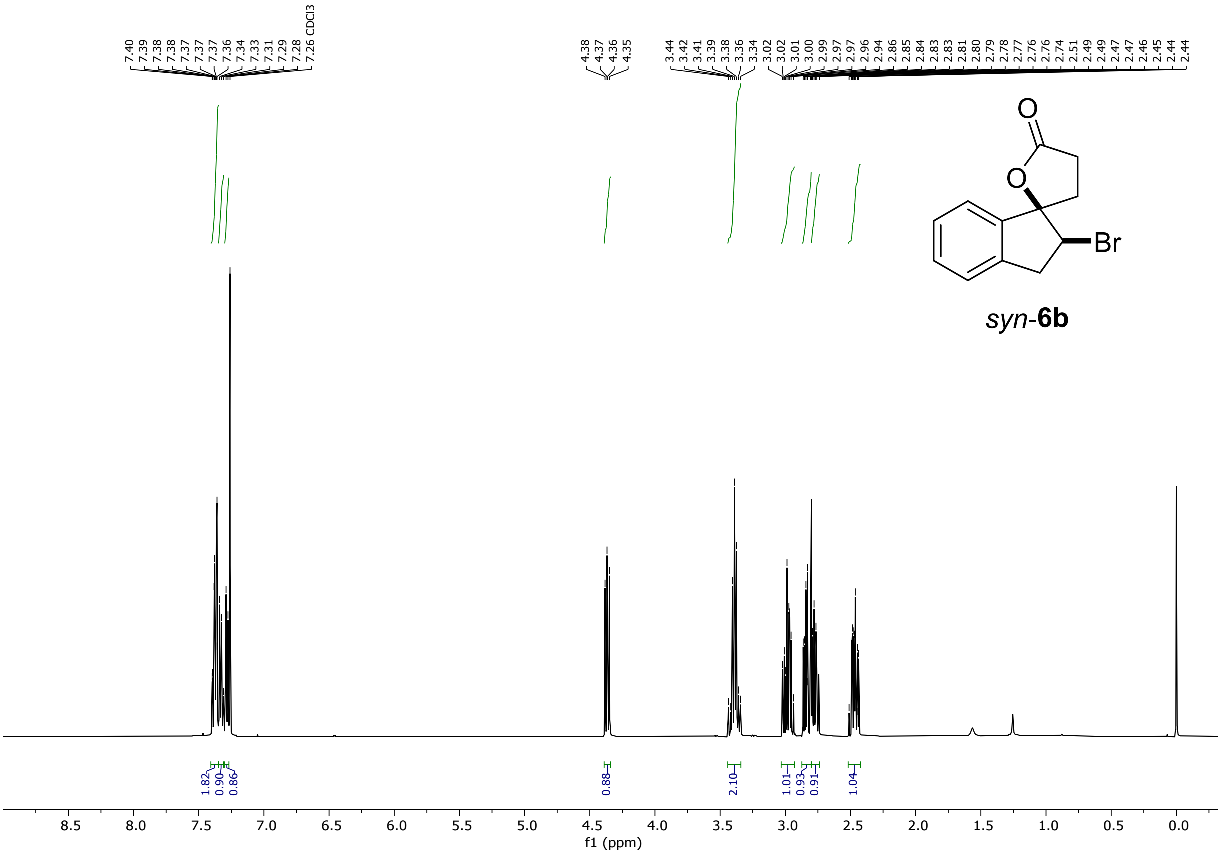
7.40
7.39
7.38
7.38
7.37
7.37
7.37
7.36
7.34
7.33
7.31
7.29
7.28
7.26 CDCl3
4.38
4.37
4.36
4.35
3.44
3.42
3.41
3.39
3.38
3.36
3.34
3.02
3.01
3.00
2.99
2.97
2.97
2.96
2.94
2.86
2.85
2.84
2.83
2.83
2.81
2.80
2.79
2.78
2.77
2.76
2.76
2.74
2.51
2.49
2.49
2.47
2.47
2.46
2.45
2.44
2.44
O
O
Br
syn-**6b**
1.82
0.90
0.86
0.88
2.10
1.01
0.93
0.91
1.04
8.5
8.0
7.5
7.0
6.5
6.0
5.5
5.0
4.5
4.0
3.5
3.0
2.5
2.0
1.5
1.0
0.5
0.0
f1 (ppm)

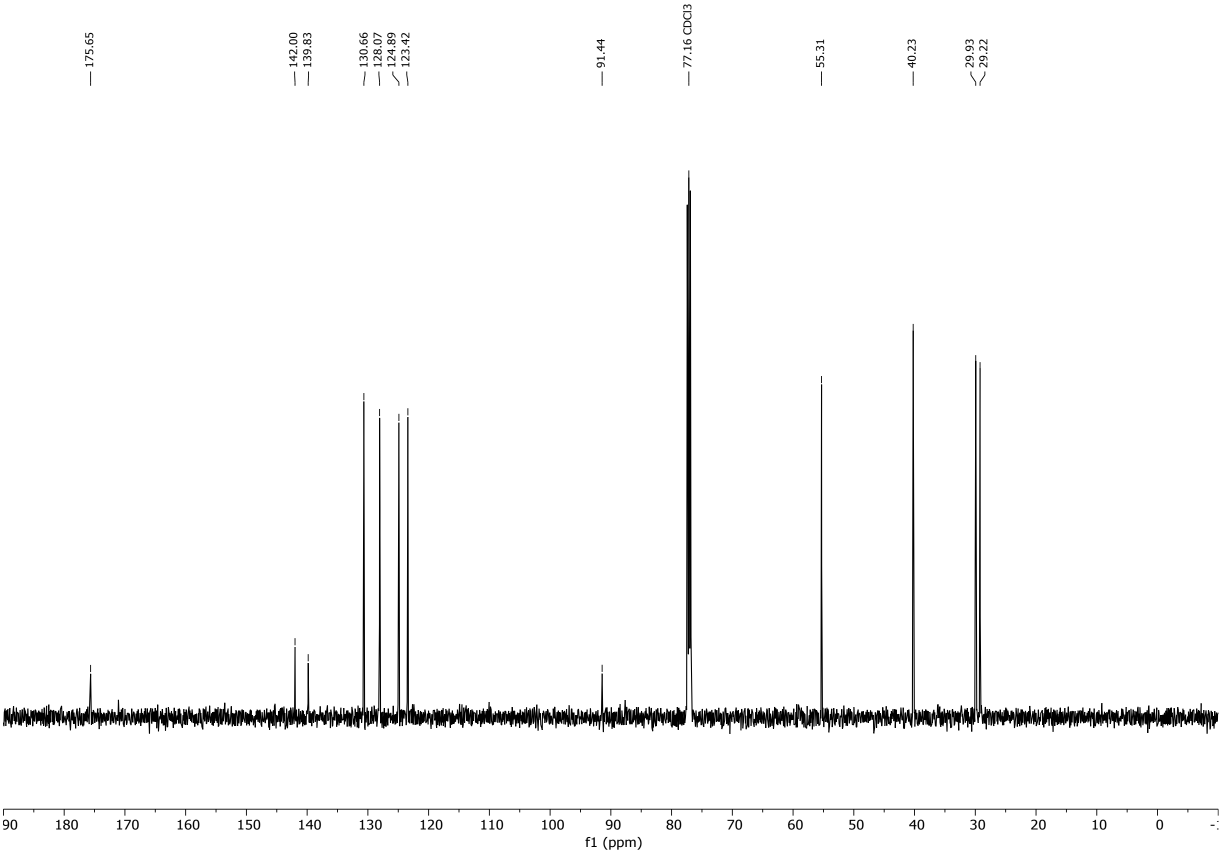
175.65
142.00
139.83
130.66
128.07
124.89
123.42
91.44
77.16 CDCl3
55.31
40.23
29.93
29.22
90
180
170
160
150
140
130
120
110
100
90
80
70
60
50
40
30
20
10
0
f1 (ppm)

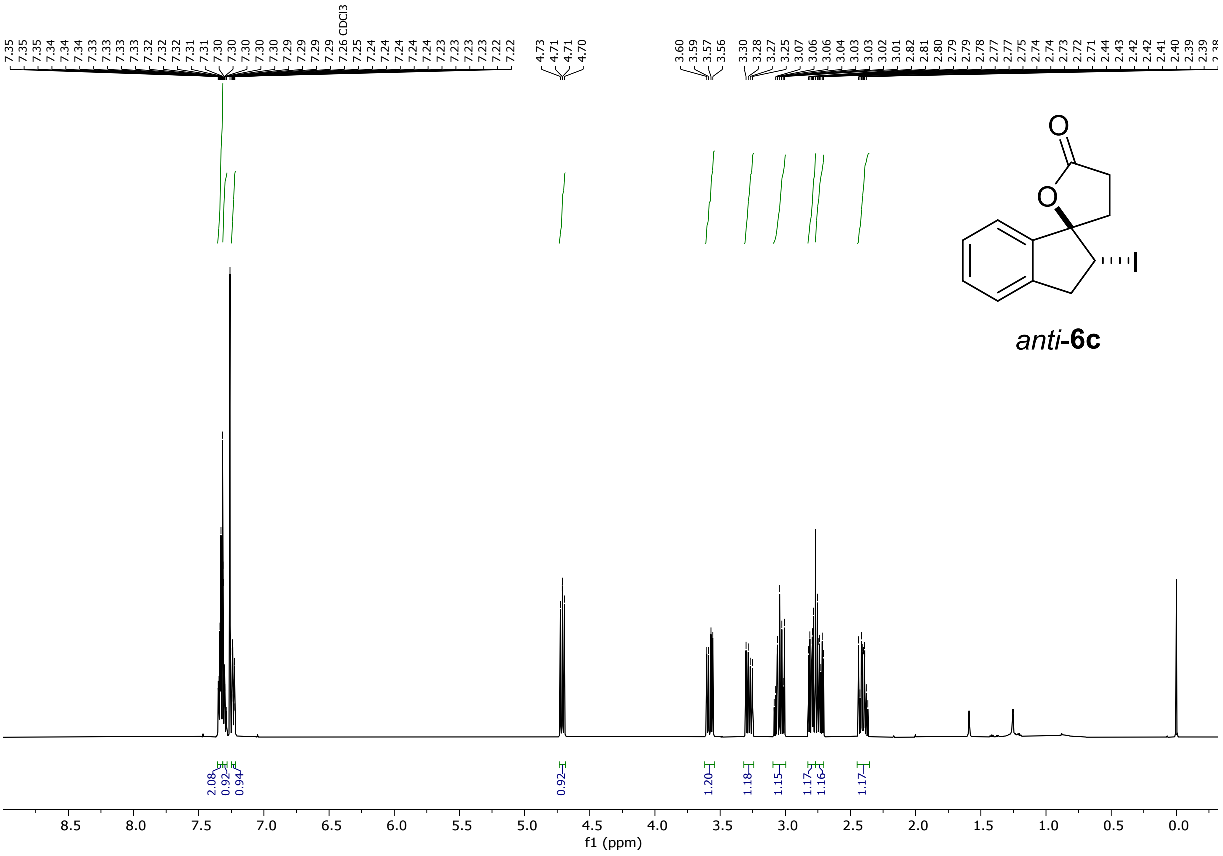
7.35 7.35 7.35 7.34 7.34 7.34 7.33 7.33 7.33 7.33 7.32 7.32 7.31 7.31 7.30 7.30 7.30 7.30 7.30 7.29 7.29 7.29 7.29 7.26 CDCl3 7.25 7.24 7.24 7.24 7.24 7.24 7.23 7.23 7.23 7.23 7.22 7.22
4.73 4.71 4.71 4.70
3.60 3.59 3.57 3.56 3.30 3.28 3.27 3.25 3.07 3.06 3.06 3.04 3.03 3.03 3.02 3.01 2.82 2.81 2.80 2.79 2.79 2.78 2.77 2.77 2.75 2.74 2.74 2.73 2.72 2.71 2.44 2.43 2.42 2.41 2.40 2.39 2.39
anti-6c
2.08 0.92 0.94 0.92 1.20 1.18 1.15 1.17 1.16 1.17
8.5 8.0 7.5 7.0 6.5 6.0 5.5 5.0 4.5 4.0 3.5 3.0 2.5 2.0 1.5 1.0 0.5 0.0
f1 (ppm)

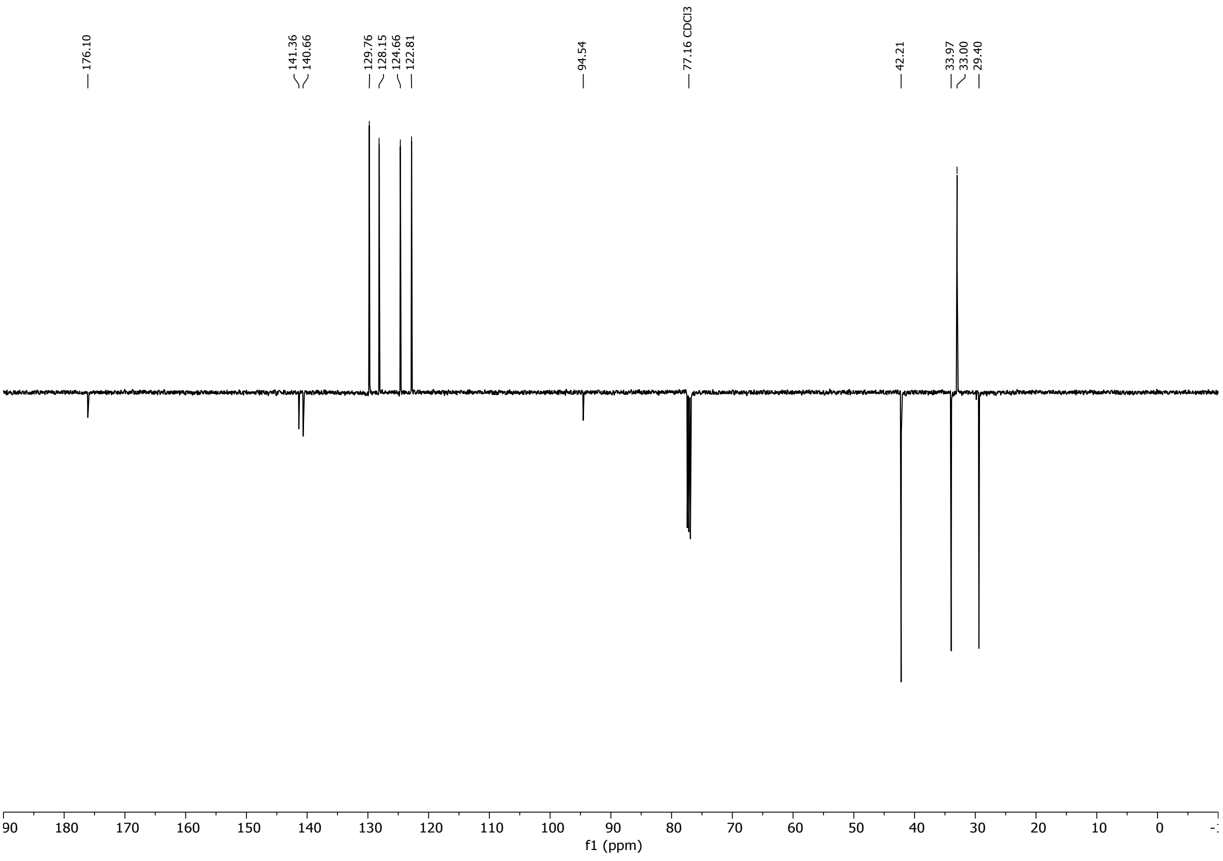
176.10
141.36 140.66
129.76 128.15 124.66 122.81
94.54
77.16 CDCl3
42.21
33.97 33.00 29.40
90 180 170 160 150 140 130 120 110 100 90 80 70 60 50 40 30 20 10 0
f1 (ppm)

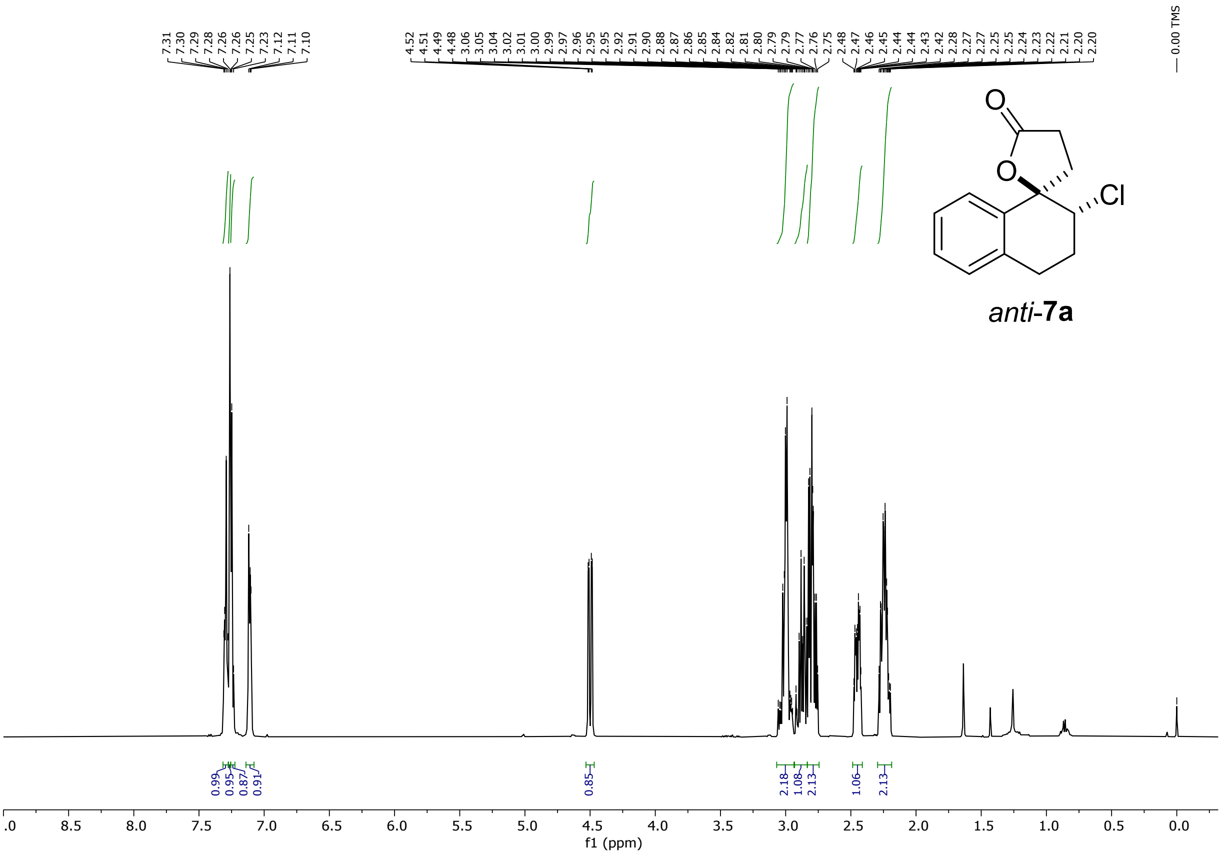
7.31 7.30 7.29 7.28 7.26 7.26 7.25 7.23 7.12 7.11 7.10
4.52 4.51 4.49 4.48 3.06 3.05 3.04 3.02 3.01 3.00 2.99 2.97 2.96 2.95 2.95 2.92 2.91 2.90 2.88 2.87 2.86 2.85 2.84 2.82 2.81 2.80 2.79 2.79 2.77 2.76 2.75 2.48 2.47 2.46 2.45 2.44 2.44 2.43 2.42 2.28 2.27 2.27 2.25 2.25 2.24 2.23 2.22 2.21 2.20 2.20
0.00 TMS
O
O
Cl
anti-7a
0.99 0.95 0.87 0.91
0.85
2.18 1.08 2.13
1.06
2.13
.0 8.5 8.0 7.5 7.0 6.5 6.0 5.5 5.0 4.5 4.0 3.5 3.0 2.5 2.0 1.5 1.0 0.5 0.0
f1 (ppm)

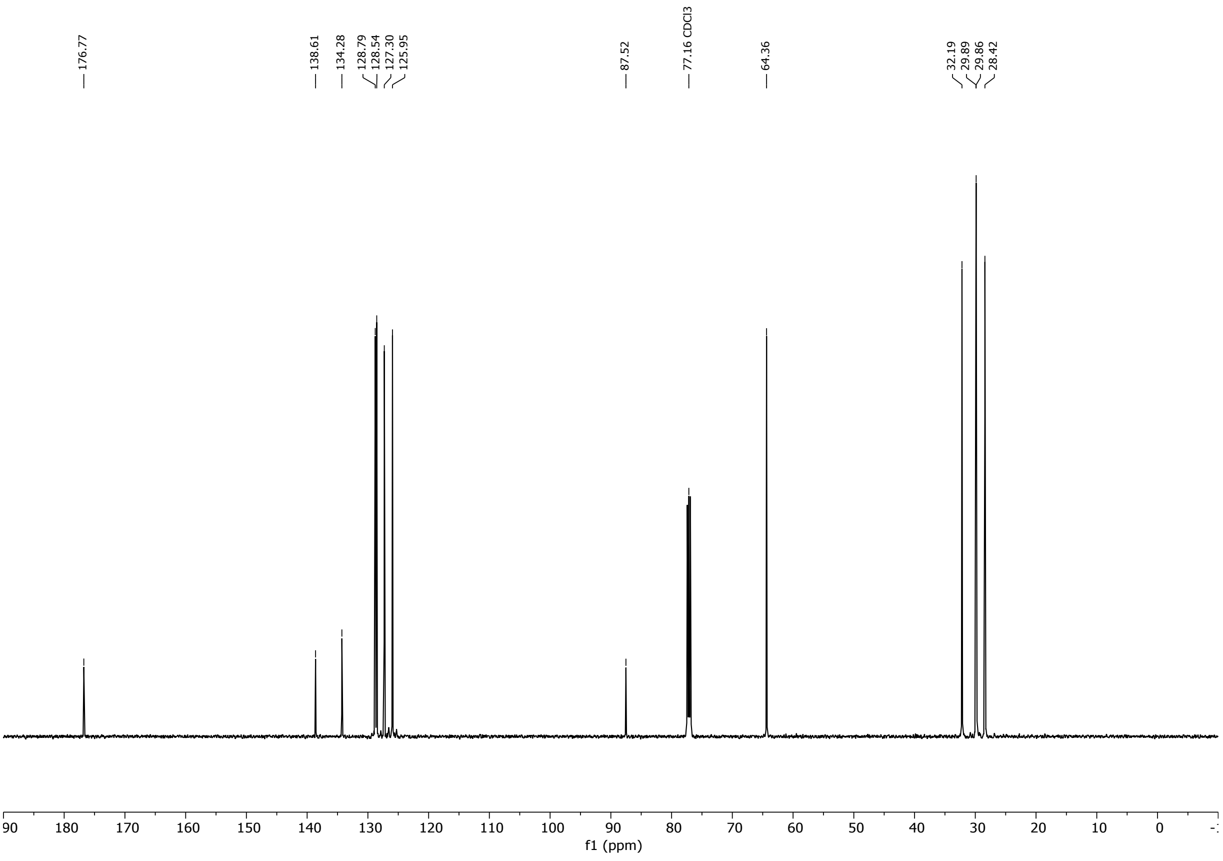
176.77
138.61 134.28 128.79 128.54 127.30 125.95
87.52
77.16 CDCl3
64.36
32.19 29.89 29.86 28.42
90 180 170 160 150 140 130 120 110 100 90 80 70 60 50 40 30 20 10 0
f1 (ppm)

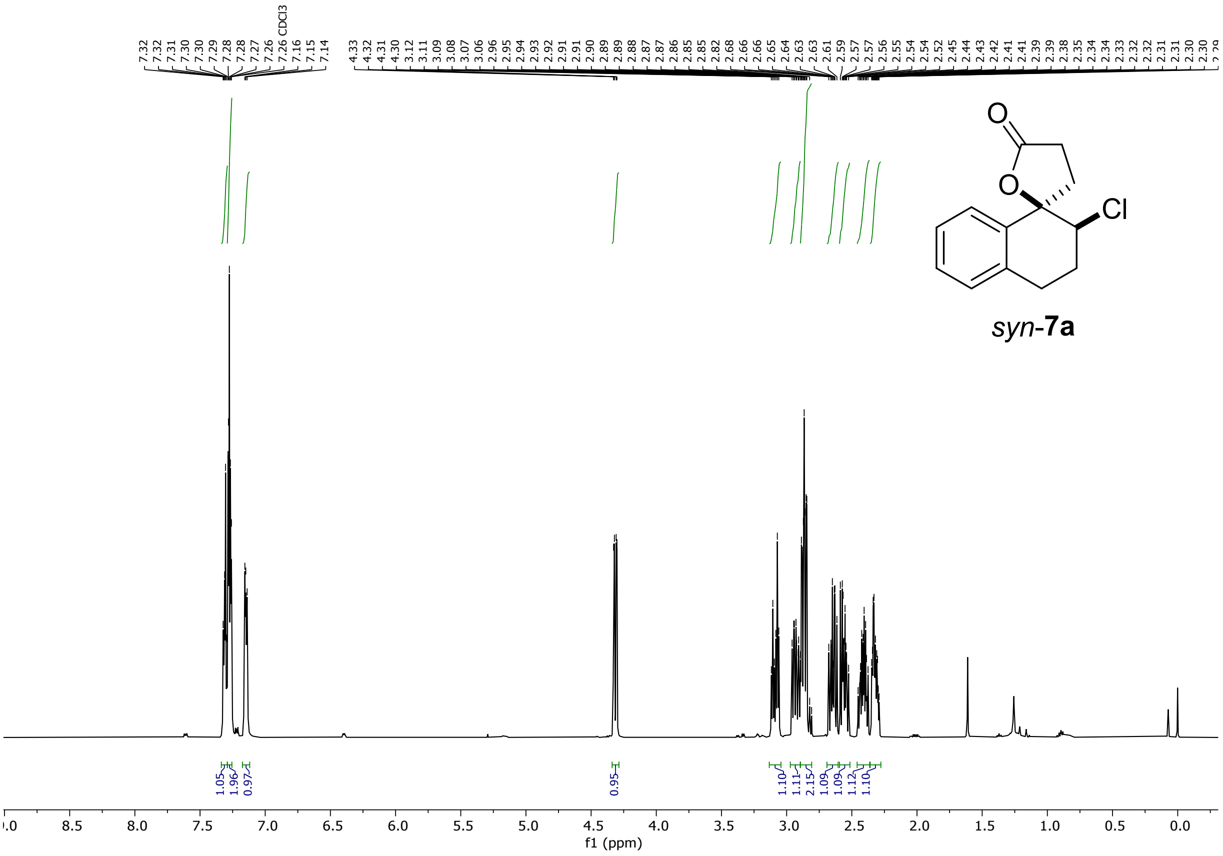
syn-7a
f1 (ppm)

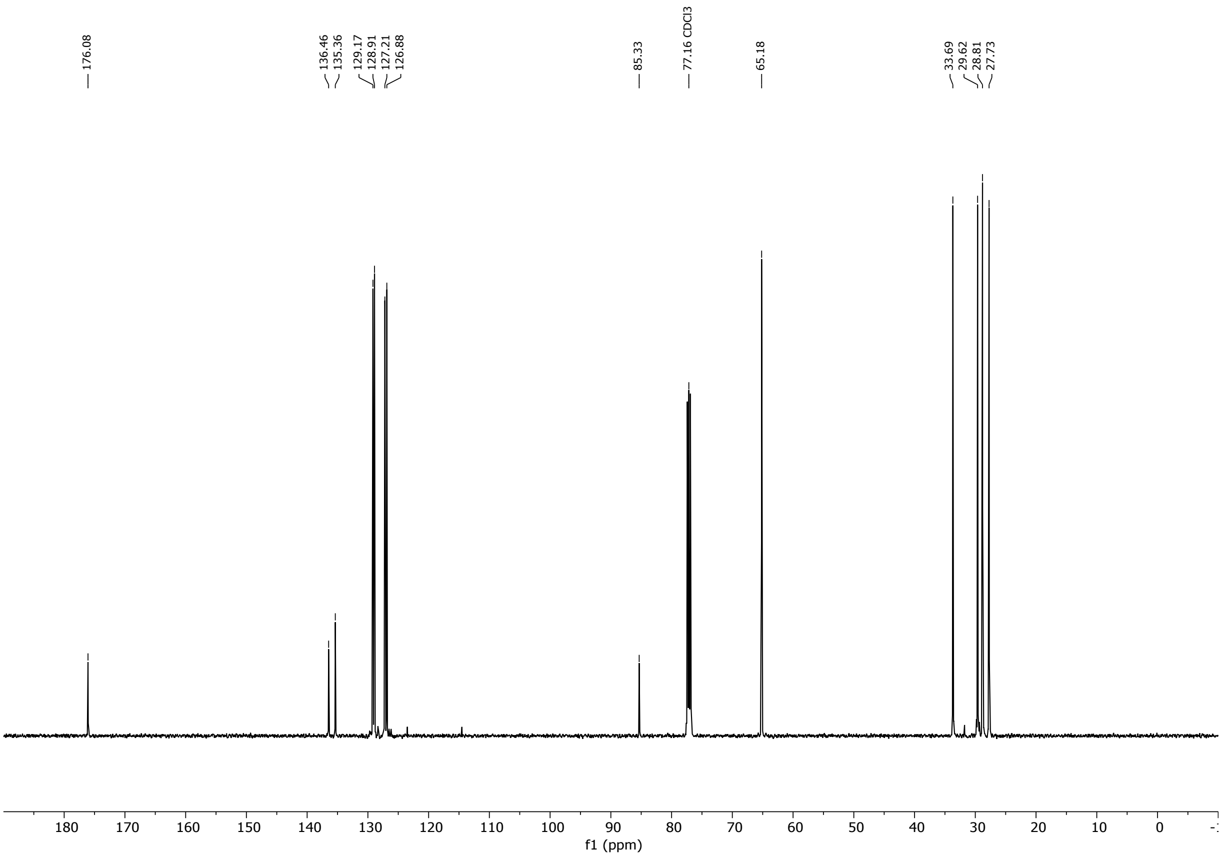
176.08
136.46
135.36
129.17
128.91
127.21
126.88
85.33
77.16 CDCl3
65.18
33.69
29.62
28.81
27.73
f1 (ppm)

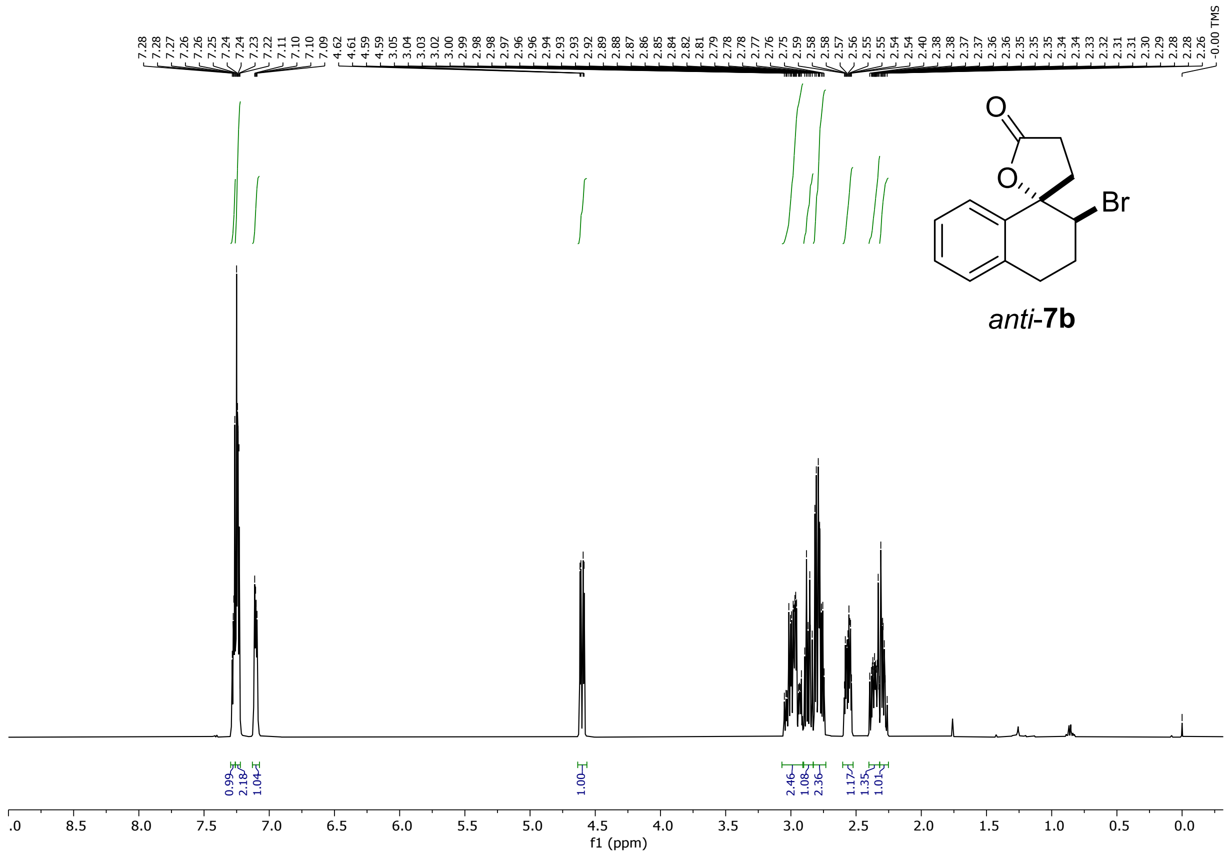
7.28 7.28 7.27 7.26 7.26 7.25 7.24 7.24 7.23 7.22 7.11 7.10 7.10 7.09
4.62 4.61 4.59 4.59 3.05 3.04 3.03 3.02 3.00 2.99 2.98 2.98 2.97 2.96 2.96 2.94 2.93 2.93 2.92 2.89 2.88 2.87 2.86 2.85 2.84 2.82 2.81 2.79 2.78 2.78 2.77 2.76 2.75 2.59 2.59 2.58 2.58 2.57 2.56 2.55 2.55 2.54 2.54 2.40 2.38 2.38 2.37 2.37 2.36 2.36 2.35 2.35 2.34 2.34 2.33 2.32 2.31 2.31 2.30 2.29 2.28 2.28 2.26
-0.00 TMS
O
O
Br
anti-7b
0.99 2.18 1.04 1.00 2.46 1.08 2.36 1.17 1.35 1.01
.0 8.5 8.0 7.5 7.0 6.5 6.0 5.5 5.0 4.5 4.0 3.5 3.0 2.5 2.0 1.5 1.0 0.5 0.0
f1 (ppm)

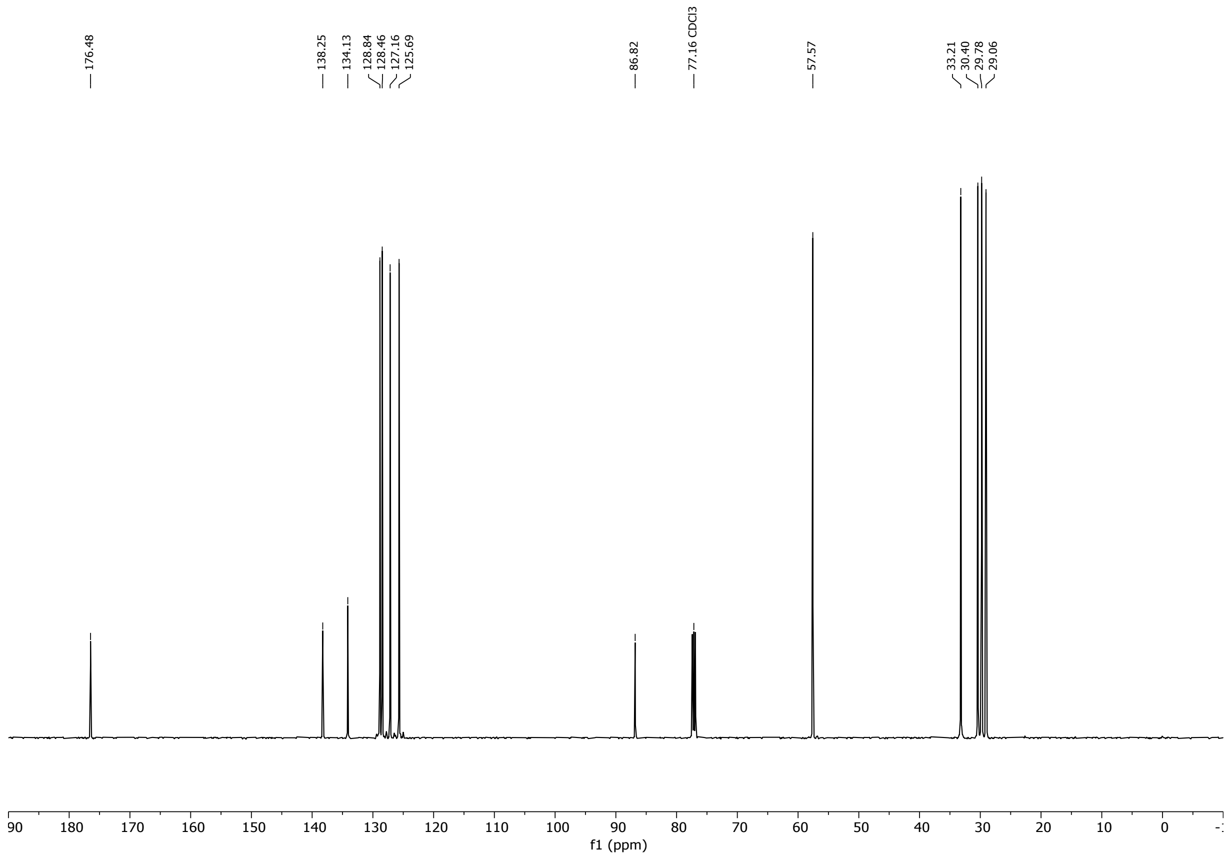
176.48
138.25 134.13 128.84 128.46 127.16 125.69
86.82
77.16 CDCl3
57.57
33.21 30.40 29.78 29.06
90 180 170 160 150 140 130 120 110 100 90 80 70 60 50 40 30 20 10 0
f1 (ppm)

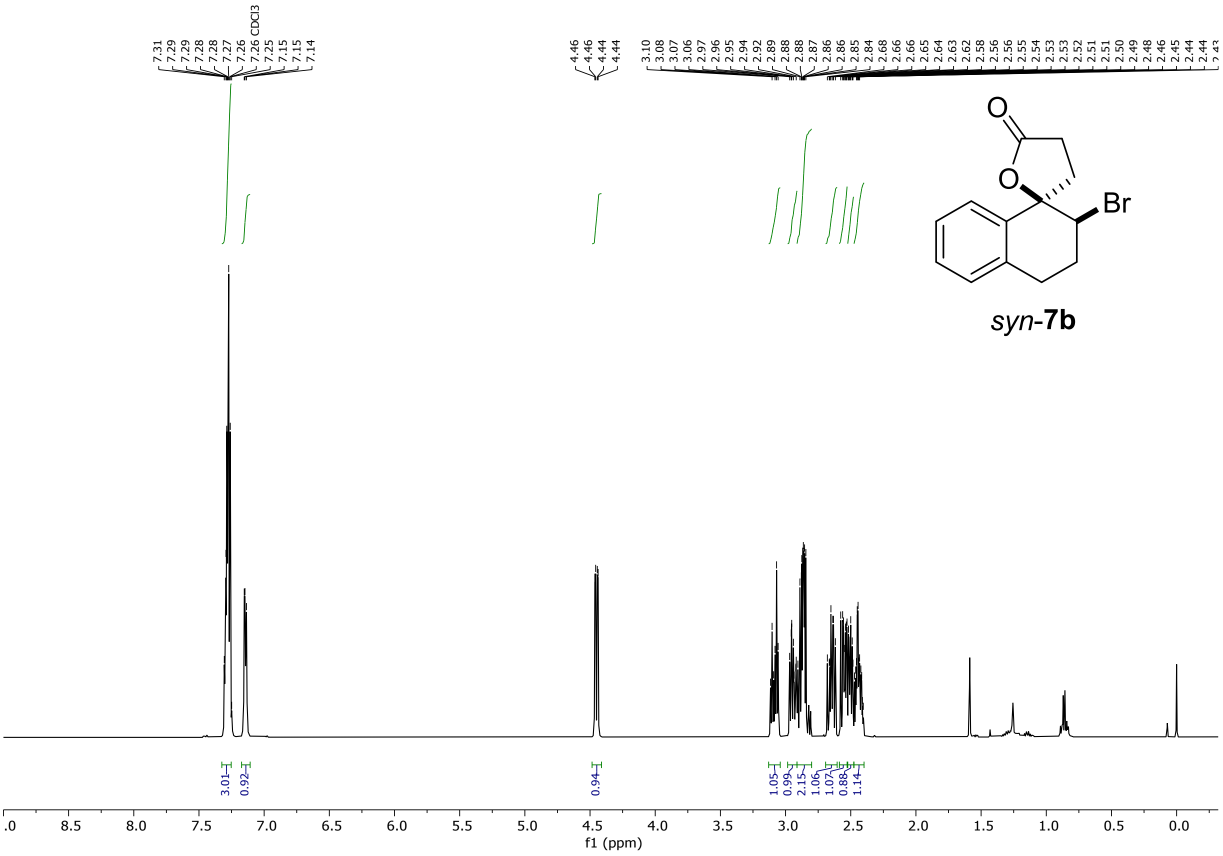
7.31 7.29 7.29 7.28 7.28 7.27 7.26 CDCl3 7.26 7.25 7.15 7.15 7.14
4.46 4.46 4.44 4.44
3.10 3.08 3.07 3.06 2.97 2.96 2.95 2.94 2.92 2.89 2.88 2.88 2.87 2.86 2.86 2.85 2.84 2.68 2.66 2.66 2.65 2.64 2.63 2.62 2.58 2.56 2.56 2.55 2.54 2.53 2.53 2.52 2.51 2.51 2.50 2.49 2.48 2.46 2.45 2.44 2.44 2.43
O
O
Br
syn-7b
3.01 0.92 0.94 1.05 0.99 2.15 1.06 1.07 0.88 1.14
f1 (ppm)

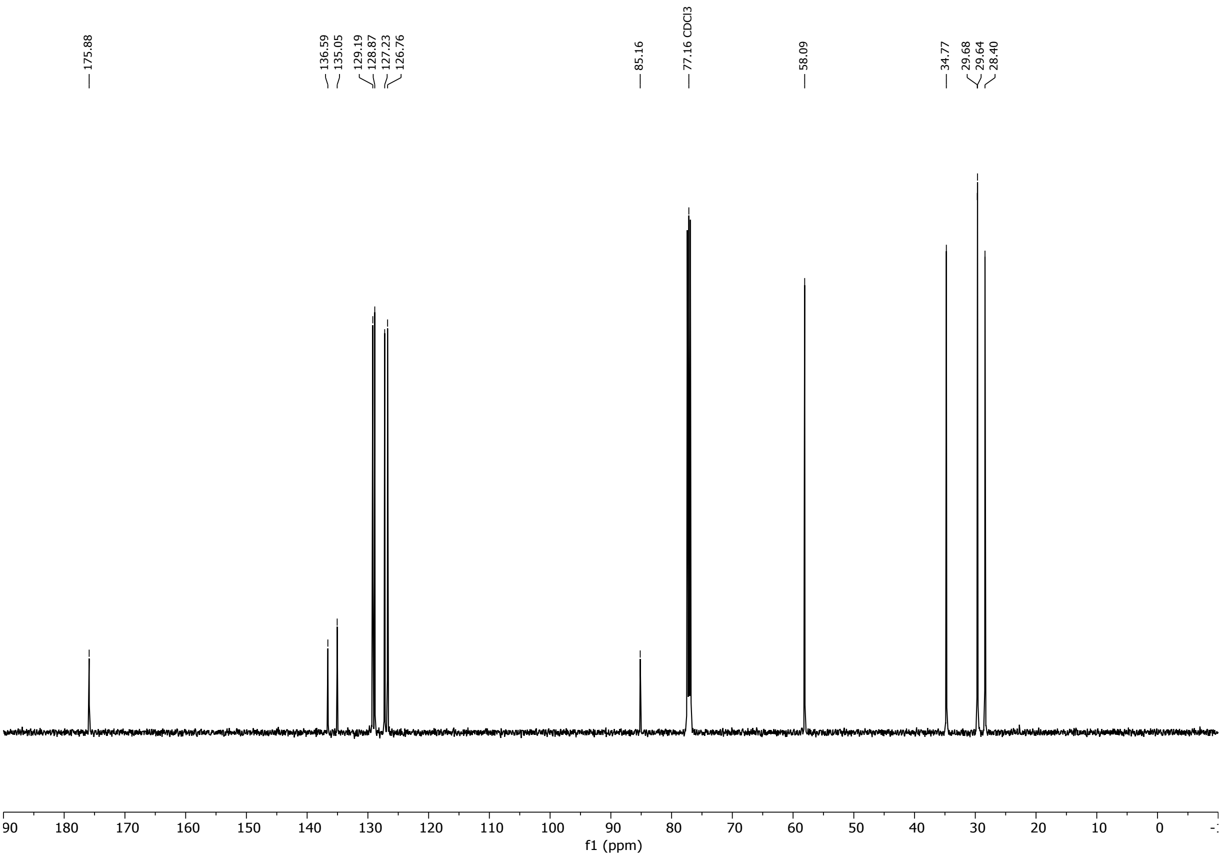
175.88
136.59 135.05 129.19 128.87 127.23 126.76
85.16
77.16 CDCl3
58.09
34.77 29.68 29.64 28.40
f1 (ppm)

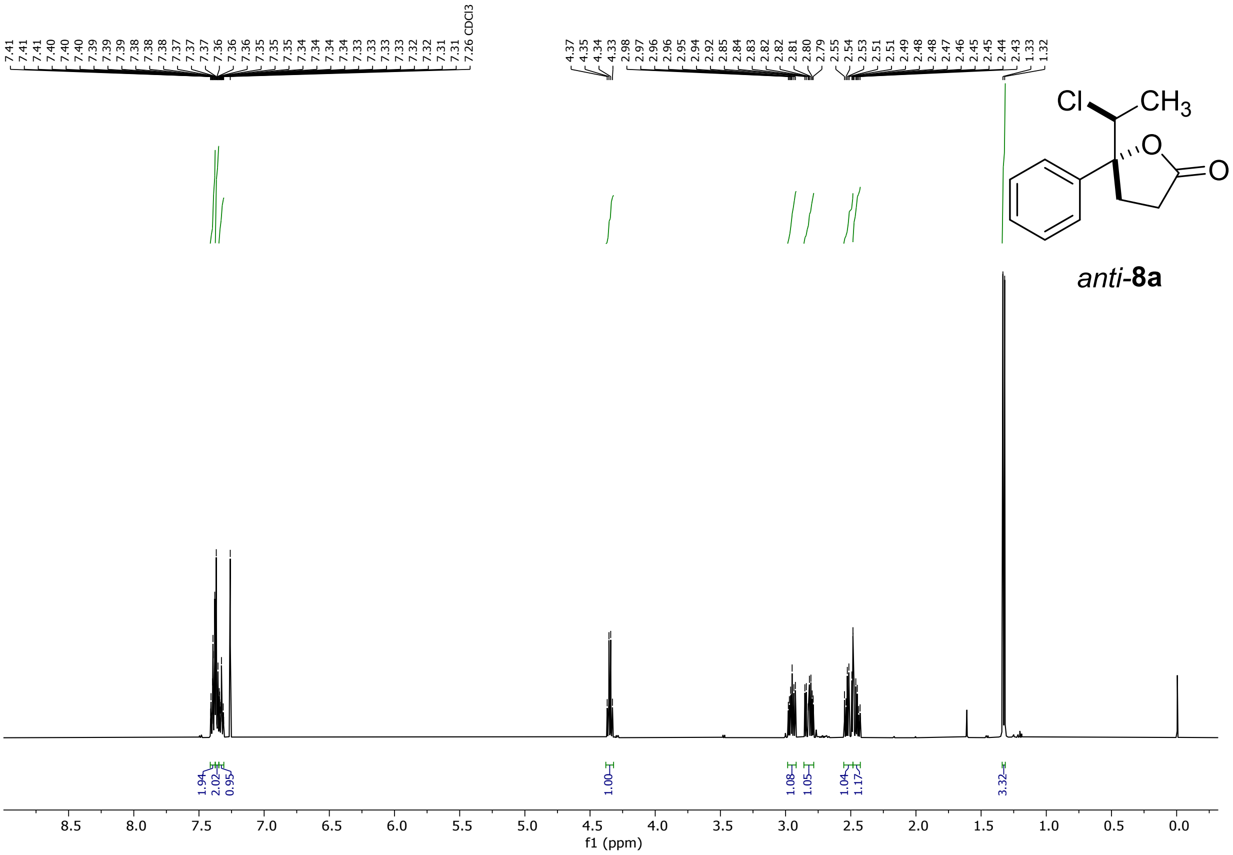

anti-**8a**

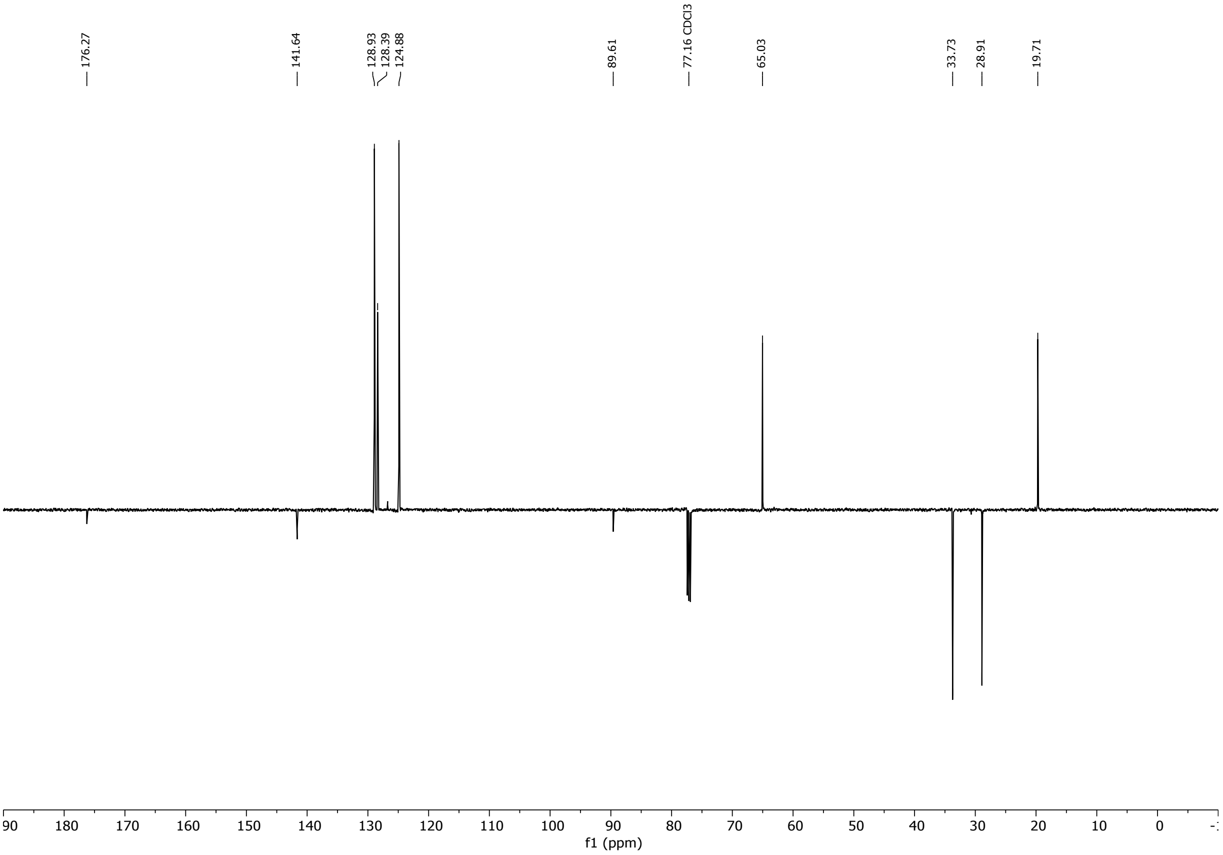

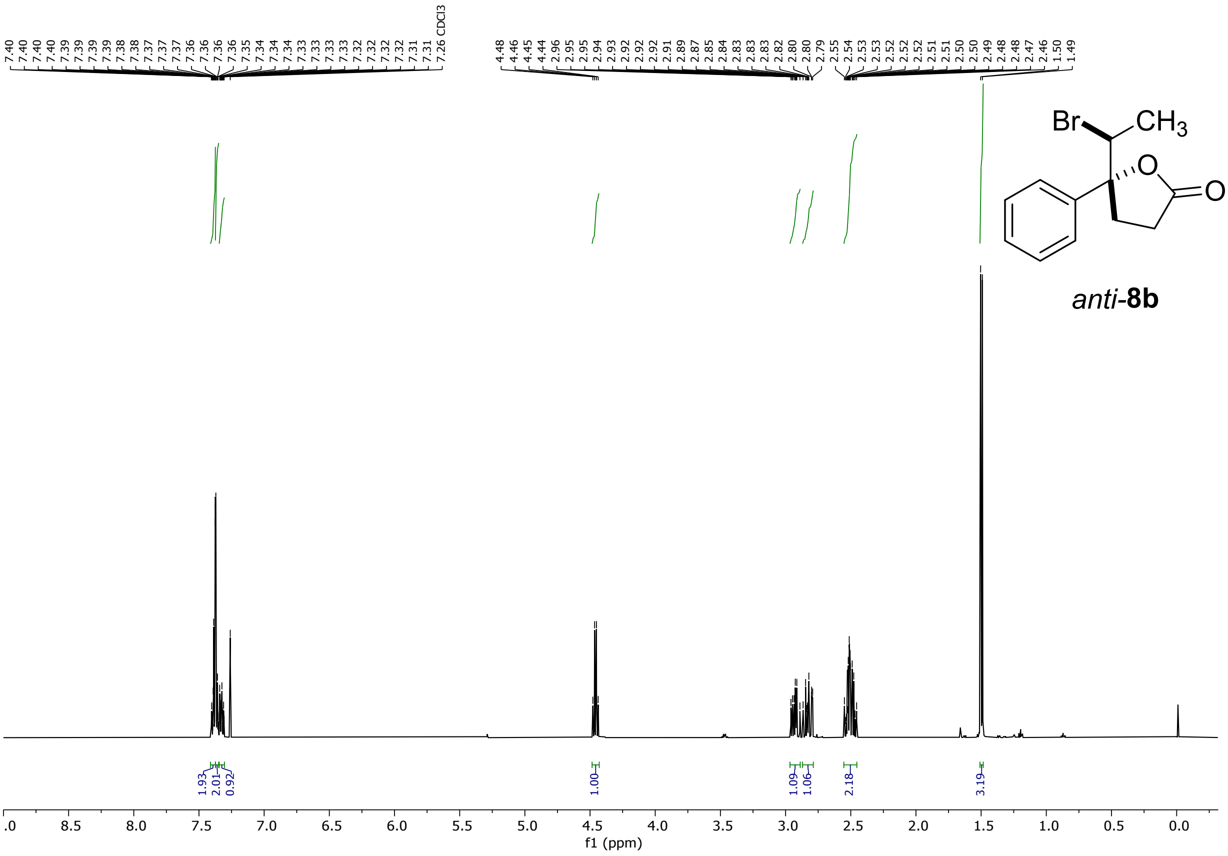
Br
CH3
O
O
7.40
7.40
7.40
7.39
7.39
7.39
7.39
7.38
7.38
7.37
7.37
7.37
7.36
7.36
7.36
7.36
7.35
7.34
7.34
7.34
7.33
7.33
7.33
7.33
7.32
7.32
7.32
7.32
7.31
7.31
7.26 CDCl3
4.48
4.46
4.45
4.44
2.96
2.95
2.95
2.94
2.93
2.92
2.92
2.91
2.89
2.87
2.85
2.84
2.83
2.83
2.82
2.80
2.80
2.79
2.55
2.54
2.53
2.53
2.52
2.52
2.52
2.51
2.50
2.50
2.49
2.48
2.48
2.47
2.46
1.50
1.49
1.93
2.01
0.92
1.00
1.09
1.06
2.18
3.19
f1 (ppm)

anti-**8b**

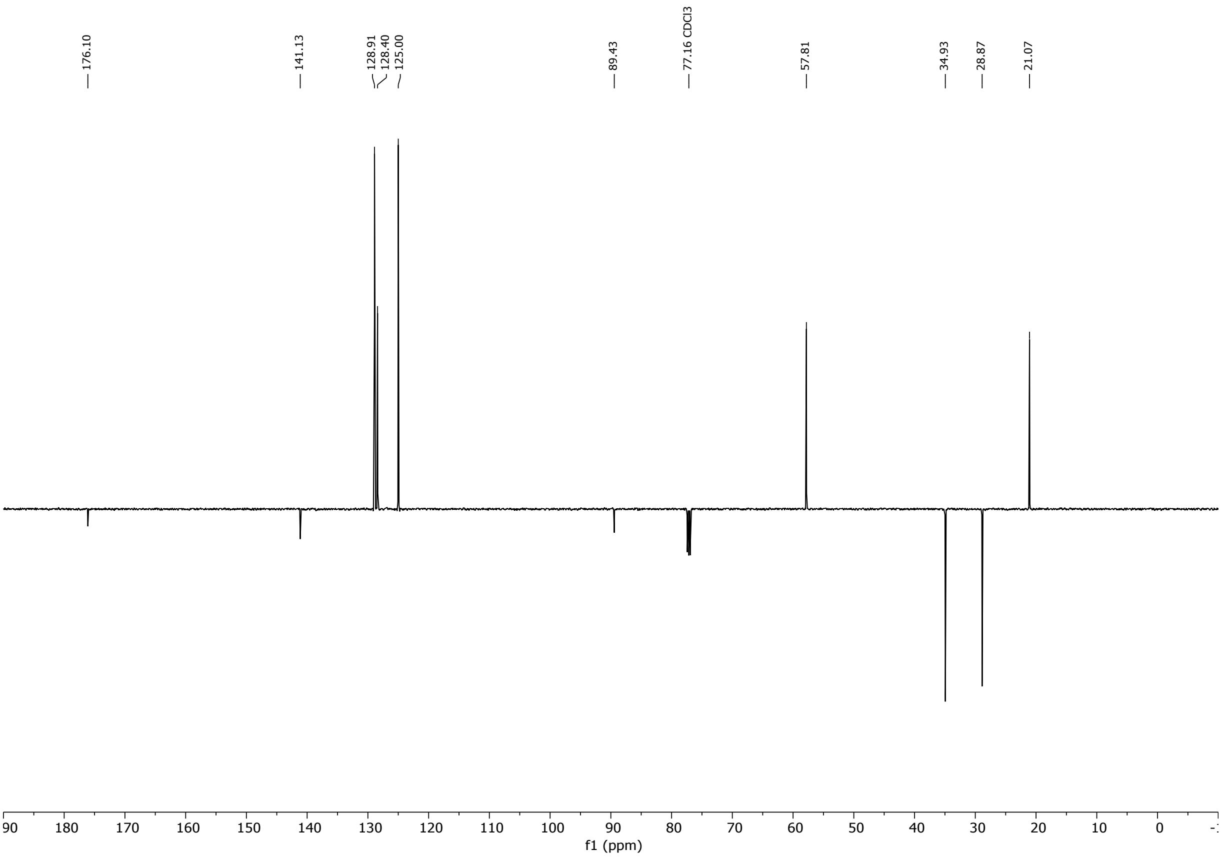
176.10
141.13
128.91
128.40
125.00
89.43
77.16 CDCl3
57.81
34.93
28.87
21.07
f1 (ppm)

3. References

(1) A. D. Becke, *J. Chem. Phys.*, 1993, **98**, 5648-5652.

(2) Y. Zhao and D. G. Truhlar, *Theor. Chem. Acc.*, 2008, **120**, 215-241.

(3) J. D. Chai and M. Head-Gordon, *Phys. Chem. Chem. Phys.*, 2008, **10**, 6615-6620.

(4) A. V. Marenich, C. J. Cramer and D. G. Truhlar, *J. Phys. Chem. B*, 2009, **113**, 6378-6396.

(5) Gaussian 16, Revision A.03, M. J. Frisch, G. W. Trucks, H. B. Schlegel, G. E. Scuseria, M. A. Robb, J. R. Cheeseman, G. Scalmani, V. Barone, G. A. Petersson, H. Nakatsuji, X. Li, M. Caricato, A. V. Marenich, J. Bloino, B. G. Janesko, R. Gomperts, B. Mennucci, H. P. Hratchian, J. V. Ortiz, A. F. Izmaylov, J. L. Sonnenberg, Williams, F. Ding, F. Lipparini, F. Egidi, J. Goings, B. Peng, A. Petrone, T. Henderson, D. Ranasinghe, V. G. Zakrzewski, J. Gao, N. Rega, G. Zheng, W. Liang, M. Hada, M. Ehara, K. Toyota, R. Fukuda, J. Hasegawa, M. Ishida, T. Nakajima, Y. Honda, O. Kitao, H. Nakai, T. Vreven, K. Throssell, J. A. Montgomery Jr., J. E. Peralta, F. Ogliaro, M. J. Bearpark, J. J. Heyd, E. N. Brothers, K. N. Kudin, V. N. Staroverov, T. A. Keith, R. Kobayashi, J. Normand, K. Raghavachari, A. P. Rendell, J. C. Burant, S. S. Iyengar, J. Tomasi, M. Cossi, J. M. Millam, M. Klene, C. Adamo, R. Cammi, J. W. Ochterski, R. L. Martin, K. Morokuma, O. Farkas, J. B. Foresman and D. J. Fox, Gaussian, Inc., Wallingford CT, 2016.

(6) S. Grimme, J. Antony, S. Ehrlich and H. Krieg, *J. Chem. Phys.*, 2010, **132(15)**, 154104.

(7) Harris, R. K.; Becker, E. D.; Cabral De Menezes, S. M.; Goodfellow, R.; Granger, P.; *Pure Appl. Chem.* 2001, **73**, 1795–1818.

(8) Wichmann, J.; Adam, G.; Röver, S.; Hennig, M.; Scalone, M.; Cesura, A. M.; Dautzenberg, F. M.; Jenck, F.; *Eur. J. Med. Chem.* 2000, **35**, 839–851.

(9) a) Cromwell, N. H. and Capps, D. B.; *J. Am. Chem. Soc.* 1952, **74**, 4448–4449. b) Finze, M.; Reybuck, S. E.; Waymouth, R. M.; *Macromolecules* 2003, **36**, 9325–9334.

(10) Keegstra, M. A.; *Tetrahedron* 1992, **48**, 2681–2690.

(11) Phipps, R. J.; McMurray, L.; Ritter, S.; Duong, H. A.; Gaunt, M. J.; *J. Am. Chem. Soc.* 2012, **134**, 10773–10776.

(12) Cornella, J.; Martin, R.; *Org. Lett.* 2013, **15**, 6298–6301.

(13) Alves, T. M. F.; Costa, M. O.; Bispo, B. A. D.; Pedrosa, F. L.; Ferreira, M. A. B.; *Tetrahedron Lett.* 2016, **57**, 3334–3338.

(14) Krishna, P. R.; Srinivas, P.; *Tetrahedron Asymmetry* 2012, **23**, 769–774.

(15) Nishikawa, Y.; Hamamoto, Y.; Satoh, R.; Akada, N.; Kajita, S.; Nomoto, M.; Miyata, M.; Nakamura, M.; Matsubara, C.; Hara, O.; *Chem. Eur. J.* 2018, **24**, 18880–18885.

(16) *APEX3* (**2016**), *SAINT* (**2015**) and *SADABS* (**2015**), Bruker AXS Inc., Madison, Wisconsin, USA.

(17) Sheldrick, G. M., *SHELXT – Integrated space-group and crystal-structure determination*, *Acta Cryst.,* 2015, **A71**, 3-8.

(18) Sheldrick, G.M., *Crystal structure refinement with SHELXL*, *Acta Cryst.*, 2015, **C71 (1)**, 3-8.

(19) *XP – Interactive molecular graphics, Version 5.1,* Bruker AXS Inc., Madison, Wisconsin, USA, 1998.
